# Fibroblasts‐specific *p16^INK4a^* exacerbates inflammageing‐mediated post‐infarction ventricular remodelling through interacting with STAT3 to regulate *NLRP3* transcription

**DOI:** 10.1002/ctm2.70344

**Published:** 2025-06-03

**Authors:** Xin Gu, Yingqiang Du, Jin'ge Zhang, Jiyu Li, Haiyun Chen, Yujie Lin, Yue Wang, Chunli Zhang, Shiyu Lin, Nannan Hao, Chengyi Peng, Jiacheng Ge, Jin Liu, Yan Liang, Yongjie Zhang, Xiaoyan Wang, Fang Wang, Jianliang Jin

**Affiliations:** ^1^ Department of Human Anatomy, School of Basic Medical Sciences, Key Laboratory for Aging & Disease, School of Biomedical Engineering and Informatics Nanjing Medical University Nanjing Jiangsu China; ^2^ Department of Cardiology The First Affiliated Hospital of Nanjing Medical University Nanjing Jiangsu China; ^3^ Department of Cardiology The Affiliated Hospital of Jiangnan University Wuxi Jiangsu China; ^4^ Department of Cardiology The Affiliated Suzhou Hospital of Nanjing Medical University, Suzhou Municipal Hospital Suzhou Jiangsu China; ^5^ Gusu School Nanjing Medical University Suzhou Jiangsu China; ^6^ The Research Center for Aging Affiliated Friendship Plastic Surgery Hospital of Nanjing Medical University Nanjing China; ^7^ Department of Rheumatology The First Affiliated Hospital of Nanjing Medical University Nanjing Jiangsu China; ^8^ Department of Geriatrics The Second Affiliated Hospital of Nanjing Medical University Nanjing Jiangsu China; ^9^ Department of Oral and Maxillofacial Surgery Affiliated Stomatological Hospital of Nanjing Medical University Nanjing Jiangsu China

**Keywords:** nanotherapy, NLRP3 inflammasome, p16^INK4a^, post‐infarction ventricular remodelling, STAT3

## Abstract

**Background and Aims:**

Inflammageing represents both a critical pathophysiological hallmark and independent risk factor for myocardial infarction (MI), with age‐related increases observed in MI incidence and severity of post‐MI ventricular remodelling. Novel therapeutic strategies targeting inflammageing‐driven mechanisms are urgently required to attenuate adverse ventricular remodelling following MI. This investigation was designed to elucidate the impact of fibroblast‐specific *p16* *^INK4a^* on inflammageing‐associated ventricular remodelling after MI and to develop a targeted nanotherapy to mitigate this process.

**Methods and Results:**

We found that p16‐mediated inflammageing positively correlated with the severity of post‐infarction ventricular remodelling in patients. *POSTN*‐driven *p16^INK4a^* knockout improved cardiac function, and reduced ventricular remodelling, myocardial inflammation and NLRP3 signalling activation following MI through downregulating STAT3‐mediated NLRP3 inflammasome and upregulating glutathione metabolism pathway in fibroblasts. *P16^INK4a^* overexpression induced NLRP3 signalling activation through upregulating *NLRP3* transcribed by STAT3 in fibroblasts. In terms of mechanisms, p16^INK4a^ interacted with STAT3, which depended on the SH2 domain of STAT3; *P16^INK4a^* promoted the interaction of EZH2 and STAT3, increased the di‐methylation on K49 and phosphorylation on Y705 of STAT3 by EZH2, and promoted *NLRP3* transcription through regulating histone modification in the *NLRP3* promoter by interfering the formation of Bmi‐1‐EZH2 or Bmi‐1‐BCL6 complex in fibroblasts. Injection of p16^INK4a^‐accumulated ageing cardiac fibroblasts, or *p16^INK4a^* overexpression adenovirus aggravated profibrosis and proinflammation in MI area. However, a novel FH peptide ‘FHKHKSPALSPV’‐neutrophil membrane proteins (NMPs)‐artificial lipid (Li) membranes‐mesoporous silica nanoparticle (MSN) core (FNLM)‐nanocaged *p16^INK4a^*‐siRNA, as a newly constructed nanomaterial drug, could prevent post‐infarction ventricular remodelling through inhibiting *NLRP3* transcription in targeted cardiac fibroblasts and ameliorating proinflammation and profibrosis.

**Conclusions:**

P16^INK4a^ drives inflammageing‐mediated post‐MI ventricular remodeling by activating STAT3/NLRP3 signaling in fibroblasts. Targeting *p16^INK4a^* via FNLM‐siRNA nanotherapy represents a novel strategy to ameliorate adverse cardiac remodelling, offering translational potential for clinical intervention.

**Key points:**

- Mechanistic Insight: P16^INK4a^ activates *NLRP3* transcription via STAT3‐EZH2 crosstalk, disrupting epigenetic complexes (Bmi‐1‐EZH2/BCL6) to exacerbate post‐MI remodelling.
- Therapeutic Innovation: A fibroblast‐targeted FNLM nanoparticle delivering *p16* ^*INK4a*^‐siRNA effectively silences *NLRP3*, reducing post‐MI inflammageing.
- Translational Impact: This study identifies p16^INK4a^‐STAT3 as a druggable axis and proposes FNLM‐*p16* ^*INK4a*^‐siRNA as a promising nanotherapy for clinical post‐MI care.

## Graphical abstract

- In ageing fibroblasts, p16^INK4a^ concurrently enhanced STAT3 di‐methylation mediated by EZH2 and suppressed the assembly of Bmi‐1‐EZH2/BCL‐6 complexes at the *NLRP3* promoter region.
- These dual regulatory actions synergistically upregulated STAT3‐dependent *NLRP3* transcription, thereby exacerbating inflammageing‐driven ventricular remodelling following myocardial infarction.
- FNLM‐nanocaged *p16^INK4a^*‐siRNA prevents post‐infarction ventricular remodelling through inhibiting *NLRP3* transcription in targeted cardiac fibroblasts.

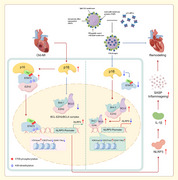

## 1 INTRODUCTION

Inflammageing drives age‐related progression of myocardial infarction (MI) incidence and post‐infarction adverse remodelling, with nearly 50% of patients developing cardiomyocyte hypertrophy, fibrosis and heart failure.^1^, ^2^, ^3^ Maladaptive inflammatory cascades exacerbate myocardial deterioration and mortality through pathological ventricular modifications.^4^, ^5^ Targeting these mechanisms is critical for developing novel anti‐inflammageing therapies to improve cardiac outcomes.

P16^INK4a^ (hereafter p16) serves as a well‐established biomarker of cell senescence.^6^, ^7^ Our recent findings demonstrate that p16 accumulation promotes high fat diet‐induced pulmonary fibrosis by enhancing inflammasome activation and metabolic dysregulation, mediated through inhibition of NEDD4L‐dependent K48‐linked SGK1 ubiquitination and degradation.^8^ Classical senescence of cardiomyocytes or cardiac stromal cells, defined by p16 upregulation, elicits a non‐canonical/classical senescence‐associated secretory phenotype (SASP) characterised by profibrotic and hypertrophic paracrine signalling.^9^ Targeted elimination of p16‐expressing senescent cells mitigates age‐associated cardiac dysfunction in murine models, specifically myocardial hypertrophy and interstitial fibrosis.^9^ P16‐positive senescent cardiomyocytes drive post‐infarction myocardial remodelling and functional impairment following MI.^10^ However, the therapeutic potential of fibroblast‐specific *p16* ablation in mitigating post‐infarction ventricular remodelling and functional impairment remains undetermined.

Inflammageing manifests as chronic, low‐grade elevation of SASP‐derived cytokines (IL‐1β, IL‐6, TNF‐α), perpetuating systemic inflammation and multi‐organ pathology.^11^, ^12^ SASP components orchestrate non‐myocyte activation within the cardiac microenvironment, driving maladaptive remodelling and contractile dysfunction.^13^, ^14^ However, whether p16 accumulation in senescent cardiac fibroblasts potentiates inflammageing‐driven post‐infarction ventricular remodelling remains unexplored.

NLRP3 inflammasome activation by pathogen‐associated and damage‐associated molecular patterns (DAMPs) orchestrates Caspase‐1‐dependent activation of IL‐1β and IL‐18 and promotes proinflammatory/profibrotic phenotype in cardiac fibroblasts.^13^ As a central mediator of inflammatory responses, this pathway also orchestrates inflammageing initiation and progression.^15^ IL‐1β serves as a critical mediator of post‐MI inflammation.^3^ Inhibiting the activation of NLRP3 inflammasome markedly reduces MI volume and attenuates left ventricular systolic dysfunction, irrespective of reperfusion status.^16^ STAT3, a core component of the JAK‐STAT signalling pathway, plays a pivotal role in the pathogenesis and progression of cardiovascular diseases.^17^, ^18^ *P16* overexpression could activate the JAK‐STAT signal pathway and promote the tyrosine phosphorylation of STAT3 at position 705 (Y705).^19^ NLRP3 is a downstream molecule of JAK‐STAT signalling pathway that mediates inflammation.^20^ Beyond canonical cell cycle regulation, p16 engages JNK1 (MAPK8)/JNK3 (MAPK10) and ERK1/2 to disrupt their kinase activity.^21^, ^22^ However, the interplay between p16 and STAT3 in regulating NLRP3‐mediated proinflammatory/fibrotic responses during cardiac fibroblast senescence remains mechanistically undefined.

This methylation modification finely tunes STAT3 functionality through modulating its nuclear translocation dynamics, altering DNA‐binding specificity and orchestrating interactions with transcriptional coactivators/corepressors.^17^, ^23^, ^24^, ^25^ Histone methyltransferase EZH2‐catalysed methylation of STAT3 employs dual regulatory mechanisms: (1) augmenting transcriptional activity through enhanced nuclear translocation and chromatin‐binding capacity; (2) stabilising the protein scaffold via di‐methylation, which primes STAT3 for Y705 phosphorylation and sustains proinflammatory signalling in pathological contexts.^17^, ^23^, ^26^ However, the potential role of p16 in exacerbating NLRP3 inflammasome‐driven cardiac inflammageing via modulation of EZH2‐catalysed STAT3 di‐methylation in fibroblasts remains undefined.

Nanoparticle‐mediated delivery of pharmacotherapeutics or oligonucleotides emerges as a targeted strategy to suppress post‐infarction hyperinflammation and enhance adaptive myocardial remodelling, thereby arresting the progression to heart failure.^27^, ^28^ Recent advances highlight the utility of non‐viral biomimetic platforms for direct cardiac fibroblast reprogramming, offering a novel in situ regenerative strategy to combat myocardial damage induced by ischaemia–reperfusion injury.^29^ The FH‐NMP‐LiMSNs (FNLM) system comprises a hybrid membrane shell consisting of FH peptide ‘FHKHKSPALSPV’‐functionalised artificial lipid (Li) membranes fused with neutrophil membrane proteins (NMPs), and a microRNA‐loaded mesoporous silica nanoparticle (MSN) core.^29^ However, whether FNLM‐nanocaged *p16*‐siRNA mitigates inflammageing‐driven post‐MI ventricular remodelling via fibroblast‐specific targeting remains unclear.

This study demonstrated that p16‐mediated inflammageing occurs in post‐infarction ventricular remodelling in patients. Fibroblast‐specific *Periostin* (*POSTN*)‐mediated *p16* deletion attenuated cardiac dysfunction and pathological remodelling in a murine MI model. In ageing fibroblasts, p16 promoted EZH2‐mediated STAT3 di‐methylation and disrupted Polycomb repressive complex (PRC)–BCL6 complex assembly at the *NLRP3* promoter, thereby enhancing STAT3‐dependent *NLRP3* transcription. This epigenetic reprogramming exacerbated post‐MI pathological remodelling through inflammageing mechanisms. Notably, FNLM‐nanocaged *p16*‐siRNA delivery attenuated inflammageing‐driven post‐MI ventricular remodelling via *NLRP3* transcriptional repression.

## 2 MATERIALS AND METHODS

Please see Complete Materials and Methods in Supporting Information 5.

### 2.1 Mice and genotyping

C57BL/6Smoc‐*Postn^em(2A‐Cre)1Smoc^* inducible Cre (hereafter *POSTN‐iCre*) mice (No. NM‐KI‐225034) with C57BL/6 background used in this study were obtained from Shanghai Model Organisms (China). *POSTN‐iCre* genotyping employed primers 5′‐GGAGCAATGGTCACTTTTGACA‐3′ (forward) and 5′‐AGGTTCTGCGGGAAACCATT‐3′ (reverse).

The *p16* (*Cdkn2a*) gene was modified using CRISPR/Cas9 technology. Briefly, the CRISPR/Cas9 system and donor template were delivered via microinjection into fertilised C57BL/6JGpt mouse oocytes. Successfully edited F0 founders were screened via polymerase chain reaction (PCR) and Sanger sequencing, followed by breeding with wild‐type C57BL/6JGpt counterparts to establish a stable F1 generation knockout model. The *p16* conditional knockout (*p16^f/f^*) murine model with C57BL/6J genetic background was generated by GemPharmatech Co., Ltd in Nanjing of China. *Loxp1* allele was detected via forward primer 5′‐CTCAGGGATGACCTGTGTTATCC‐3′ and reverse primer 5′‐TGGACTACCAGAATACGCTGGAG‐3′; *Loxp2* allele was detected using primer below: Forward: 5′‐CAGCTCTTGCGTAAGCAGATTTG‐3′ and Reverse: 5′‐CACAACGGGTTCTTCTGTTAGTCC‐3′. The *p16^f/f^POSTN‐iCre* mice were generated through sequential mating of *p16^f/f^* mice with *POSTN‐iCre* transgenic mice.

One week prior to MI induction, the *p16^f/f^POSTN‐iCre* mice received tamoxifen (50 mg/kg) dissolved in corn oil via daily intraperitoneal (i.p.) injection for 5 consecutive days to trigger genetic recombination. Twelve‐month‐old *p16* homozygous (*p16*‐KO) male mice and their wild‐type (WT) male littermates were generated and genotyped as our described previously.^8^, ^21^

All animal procedures strictly complied with National Institutes of Health (NIH) guidelines (Publication No. 8, revised 2011) and were approved by Institutional Animal Care and Use Committee of Nanjing Medical University (IACUC‐1706001).

Prior to MI model establishment, surgical anaesthesia was induced in mice via i.p. delivery of pentobarbital sodium (50 mg/kg body weight). For cardiac function assessment, anaesthesia was maintained using isoflurane (4% induction, 1.5% maintenance) delivered in oxygen, and Color Doppler echocardiography was performed. A humane endpoint was achieved through i.p. treatment of pentobarbital sodium (100 mg/kg).

### 2.2 Patient enrolment, blood procurement and peripheral blood mononuclear cells isolation

Isolation for blood sampling was approved by the Ethics Committee of The Affiliated Hospital of Jiangnan University (Approval No. K2022031K01), ensuring all procedures strictly adhered to ethical principles outlined in Declaration of Helsinki. All participants had signed informed consent. Peripheral blood mononuclear cells (PBMCs) were isolated from collected blood samples following manufacturer‐provided protocols. MI patients were stratified into two cohorts in accordance with p16 expression levels detected in PBMCs, with coronary artery disease patients serving as controls. Following standardised pharmacological intervention, participants underwent 1‐year follow‐up evaluations encompassing cardiac function assessments, haematological analyses and magnetic resonance imaging (MRI) examinations. Detailed baseline demographics of coronary artery disease and MI cohorts are presented in Supporting Information 6.

Additionally, we recruited patients at 6–12 months post‐MI and age‐matched healthy controls. Participants were stratified into two age cohorts: 20–25 years (young group) and 55–65 years (older group, including older MI patients). Following collection of peripheral blood samples, serum and PBMCs were isolated according to standardised protocols. All prior enrolled participant had signed informed consent. Detailed baseline characteristics of age‐stratified MI patients are presented in Supporting Information 7.

### 2.3 Human myocardial tissue samples

Human myocardial tissue samples were obtained from 25 autopsy donors through Department of Human Anatomy of Nanjing Medical University. The study was performed in strict adherence to ethical guidelines established by the 1975 Declaration of Helsinki. Both anatomical procedures and experimental protocols received approval from the Nanjing Medical University Ethics Committee (Approval No. 2019–902). Donors were enrolled, aged 39–94 years, without documented history of neoplasms, congenital heart defects, valvular abnormalities, autoimmune disorders, chronic infections, inflammatory conditions, tuberculosis, syphilis, acquired immunodeficiency syndrome or other significant comorbidities prior to demise.

### 2.4 Statistical analysis

Quantitative data are reported as mean ± standard error of the mean (SEM) from ≥3 independent experiments. Statistical analyses were conducted using SPSS v22.0 and GraphPad Prism v7.0. Intergroup comparisons utilised unpaired Student's *t*‐tests, while one‐way analysis of variance (ANOVA) was employed for multiple group analyses. Statistical significance was defined as *p* < .05 (two‐sided). Correlation analyses applied Pearson's coefficient for normal distributions and Spearman's for non‐normal ones, as previously described.^21^

## 3 RESULTS

### 3.1 P16‐mediated inflammageing correlated positively with post‐infarction ventricular remodelling severity in patients

**FIGURE 1:**
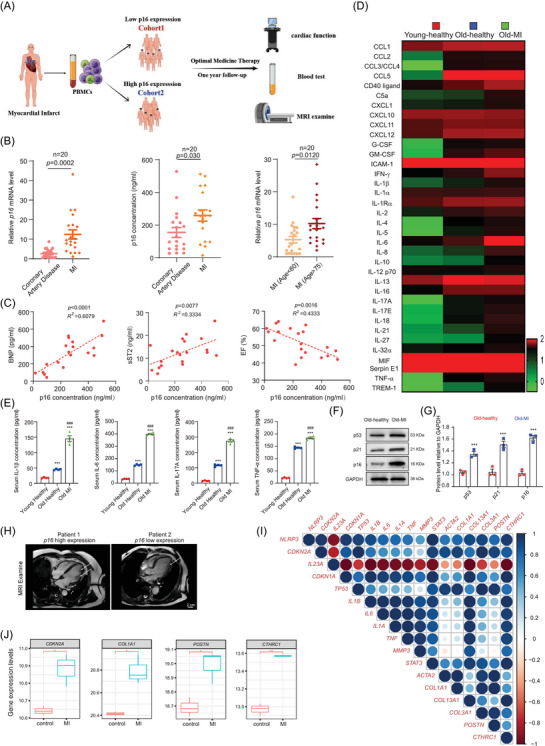
P16‐mediated inflammageing correlates positively with post‐infarction ventricular remodelling severity in patients. Peripheral blood samples were collected from patients diagnosed with coronary artery disease (CAD) or post‐myocardial infarction (MI) ventricular remodelling. (A) Diagram of peripheral blood mononuclear cells (PBMCs) from MI were collected and were divided into two cohorts based on p16 expression. Both cohorts were treated with optimally medicine and evaluated 1 year follow‐up by cardiac function, blood test and MRI examine. (B) *P16* mRNA level detected with real‐time quantitative polymerase chain reaction (RT‐qPCR) and p16 protein level (ng/mL) detected with ELISA assay. Twenty samples per group were used for experiments. A two‐sided value of *p* < .05 was considered statistically significant, unpaired Student's *t*‐test. (C) Correlation of p16 concentration (ng/mL) with BNP (pg/mL), sST2 (ng/mL) and ejection fraction (EF; %) by principal component analysis. Twenty samples per group were used for experiments. Gaussian distributed data were analysed by Pearson's *r* and non‐Gaussian distributed data were analysed by Spearman's *r*. *P* values were two‐sided and values less than .05 was considered statistically significant. Serum samples from young (aged 20–25)‐healthy people, and old (aged 55–65)‐healthy people or old patients with ventricular remodelling following MI were collected, and detected the protein levels of inflammation‐related factors with solid‐phase microarray and ELISA assay. (D) Cluster map of serum inflammatory factor with solid‐phase microarray. (E) ELISA assay for IL‐1β, IL‐6, IL‐17A and TNF‐α. Four biological replicates were used per experiment. ^***^ *p* < .001 compared to young‐healthy group; ^###^ *p* < .001 compared to old‐healthy group, one‐way analysis of variance (ANOVA) test. (F) Western blots of PBMCs extracts showing p53, p21 and p16; GAPDH was used as the loading control. (G) Protein levels relative to GAPDH were assessed by densitometric analysis. Three biological replicates were used per experiment, ^***^ *p* < .001 compared with old‐healthy group. (H) Differences in magnetic resonance imaging (MRI) examine between *p16* high expression and *p16* low expression in MI patients. (I) RNA‐seq data (GSE115031) analysis of the human organoid MI model. Correlation analysis of gene expression by corrplot package in RStudio. (J) The mRNA levels of indicated genes including *p16* (*CDKN2A*), *COL1α1*, *POSTN* and *CTHRC1*. Three biological replicates were used per experiment. ^*^ *p* < .05, ^**^ *p* < .01 compared with control group, unpaired Student's *t*‐test.

The patients with MI were divided into cohort 1 and cohort 2 by *p16* expression of PBMCs. They were treated with optimal medication therapy and followed up 1 year later. The prognosis was assessed by cardiac function, blood test and MRI examination (Figure 1A). Compared to patients with coronary artery disease, the expressions of *p16* mRNA and protein were increased in MI patients. Meantime, p16 was higher in the MI patients (Age > 75) than MI patients (Age < 60; Figure 1B). When they were treated with optimal medication therapy and followed up 1 year later, a significant positive correlation between p16 protein expression and circulating levels of brain natriuretic peptide (BNP) and sST2, while exhibiting an inverse relationship with left ventricular ejection fraction (LVEF; Figure 1C). To observe the proinflammatory factors of serum in young (aged 20–25)‐healthy people, old (aged 55–65)‐healthy people and old patients with ventricular remodelling following MI, proteome profiler human cytokine array and ELISA assay were processed and showed that compared with old‐healthy people, a significant reduction in circulating levels of proinflammatory cytokines, notably IL‐1β, IL‐6, IL‐17A and TNF‐α, was observed in peripheral blood samples collected from young‐healthy adults, however, a significant increase of them was shown in old patients with ventricular remodelling following MI (Figure 1D,E). Compared with old‐healthy people, protein levels of p53, p21 and p16 were obviously increased in PBMCs from old patients with ventricular remodelling following MI (Figure 1F,G). In MRI results, fibrosis area was decreased in post‐MI patients with low *p16* expression compared to those with high *p16* expression (Figure 1H). Our findings demonstrate elevated proinflammatory status and accelerated ageing indices in MI patients with ventricular remodelling, exhibiting characteristic features of inflammageing.

We then analysed the RNA sequencing (RNA‐seq; GSE115031) of the human organoid MI model and found that *NLRP3* mRNA levels are positively correlated with ageing markers *CDKN2A* (*p16*), *CDKN1A* (*p21*) and *TP53* (*p53*), cytokines *IL‐1β*, *IL‐6*, *TNF* and *IL‐1α*, and key ventricular remodelling genes^30^ *ACTA2 (α‐smooth muscle actin*, *α‐SMA)*, *COL1α1*, *COL3α1*, *POSTN* and *CTHRC1* (Figure 1I). Additionally, this RNA‐seq analysis revealed a significant upregulation of *p16*, *COL1α1*, *POSTN* and *CTHRC1* mRNA transcripts in infarcted cardiac organoids compared to sham‐operated controls (Figure 1J). These results suggested that senescent fibroblast might contribute to inflammageing‐driven post‐infarction ventricular remodelling.

### 3.2 *POSTN*‐mediated *p16* deletion enhances cardiac function and alleviates ventricular remodelling, myocardial inflammation and NLRP3 inflammasome activation in MI mice

**FIGURE 2:**
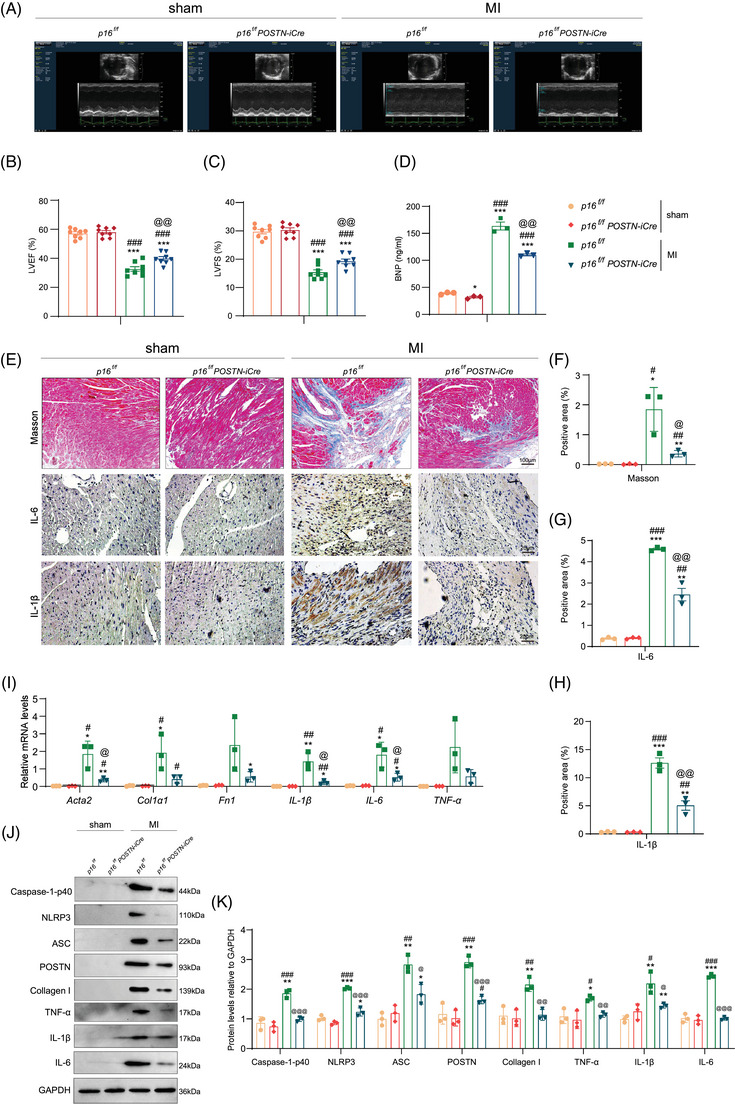
*POSTN*‐mediated *p16* deletion enhances cardiac function and alleviates ventricular remodelling, myocardial inflammation and NLRP3 inflammasome activation in myocardial infarction (MI) mice. Twelve‐month‐old *p16^f/f^POSTN‐iCre* mice and *p16^f/f^* littermates were anaesthetised via intraperitoneal injection of pentobarbital sodium (50 mg/kg), followed by induction of acute MI. After a 4‐week observation period, comprehensive evaluations were performed on these animals. One week prior to MI induction, the *p16^f/f^POSTN‐iCre* mice received tamoxifen (50 mg/kg) dissolved in corn oil via daily intraperitoneal injection for 5 consecutive days to trigger genetic recombination. (A) Mice were anaesthetised by inhaling isoflurane at a 1:1 concentration with oxygen (4% induction concentration, 1.5% maintenance concentration) and detected by Color Doppler echocardiography. (B, C) Left ventricular ejection fraction (LVEF) and left ventricular shortening fraction (LVFS). The experiments were performed with eight mice per group. (D) The myocardial tissue in the infarcted area was examined for BNP protein levels (ng/mL) by ELISA assay. (E) Representative micrographs of paraffin‐embedded heart ventricular wall sections of mice stained for Masson's trichrome (Masson) staining and stained immunohistochemically for IL‐6 and IL‐1β. (F–H) The percentage of cells or areas positive for Masson's trichrome‐labelled interstitial fibres, IL‐6 and IL‐1β relative to total cells or areas. (I) Real‐time quantitative polymerase chain reaction (RT‐qPCR) of cardiac tissue extracts showing *Acta2* (encoded α‐SMA), *Col1α1*, *Fn1* (encoded Fibronectin), *IL‐1β*, *IL‐6*, *TNF‐α* and *Gapdh* was the loading control. (J) Western blots of the myocardial tissue extract in the infarcted area showing Caspase‐1‐p40, NLRP3, ASC, POSTN, Collagen I, TNF‐α, IL‐1β, IL‐6 and GAPDH was the loading control. (K) Protein levels relative to *p16^f/f^*‐Sham mice were assessed by densitometric analysis. Three biological replicates were used per experiment. ^*^ *p* < .05, ^**^ *p* < .01, ^***^ *p* < .001 compared to *p16^f/f^*‐Sham mice group; ^#^ *p* < .05, ^##^ *p* < .01, ^###^ *p* < .001 compared to *p16^f/f^POSTN‐iCre* ‐Sham mice group; ^@^ *p* < .05, ^@@^ *p* < .01, ^@@@^ *p* < .001 compared to *p16^f/f^*‐MI mice group, one‐way analysis of variance (ANOVA) test.

To investigate the role of fibroblasts in inflammageing during post‐infarction ventricular remodelling, we generated fibroblast‐specific *p16^f/f^POSTN‐iCre* mice and *p16^f/f^* littermates, which were subjected to MI. Our findings revealed that *POSTN*‐mediated *p16* deletion resulted in a significant elevation of LVEF and left ventricular shortening fraction (LVFS), coupled with a marked reduction in circulating BNP concentrations in the MI murine model (Figure 2A–D). The Masson's trichrome (Masson)‐, IL‐6‐ and IL‐1β‐positive areas in *p16^f/f^POSTN‐iCre* MI mice was significantly lower than that in *p16^f/f^* MI mice (Figure 2E–H). Meanwhile, an obvious decrease was shown in the mRNA levels of *Acta2* (encoded α‐SMA), *IL‐1β* and *IL‐6*, and in the protein levels of Caspase‐1‐p40, NLRP3, ASC, POSTN, Collagen I, TNF‐α, IL‐1β and IL‐6 in *p16^f/f^POSTN‐iCre* MI mice compared to *p16^f/f^* MI mice (Figure 2I–K).

### 3.3 *POSTN*‐mediated *p16* deletion suppresses STAT3‐dependent NLRP3 inflammasome activation while enhancing glutathione metabolic pathway in fibroblasts

**FIGURE 3:**
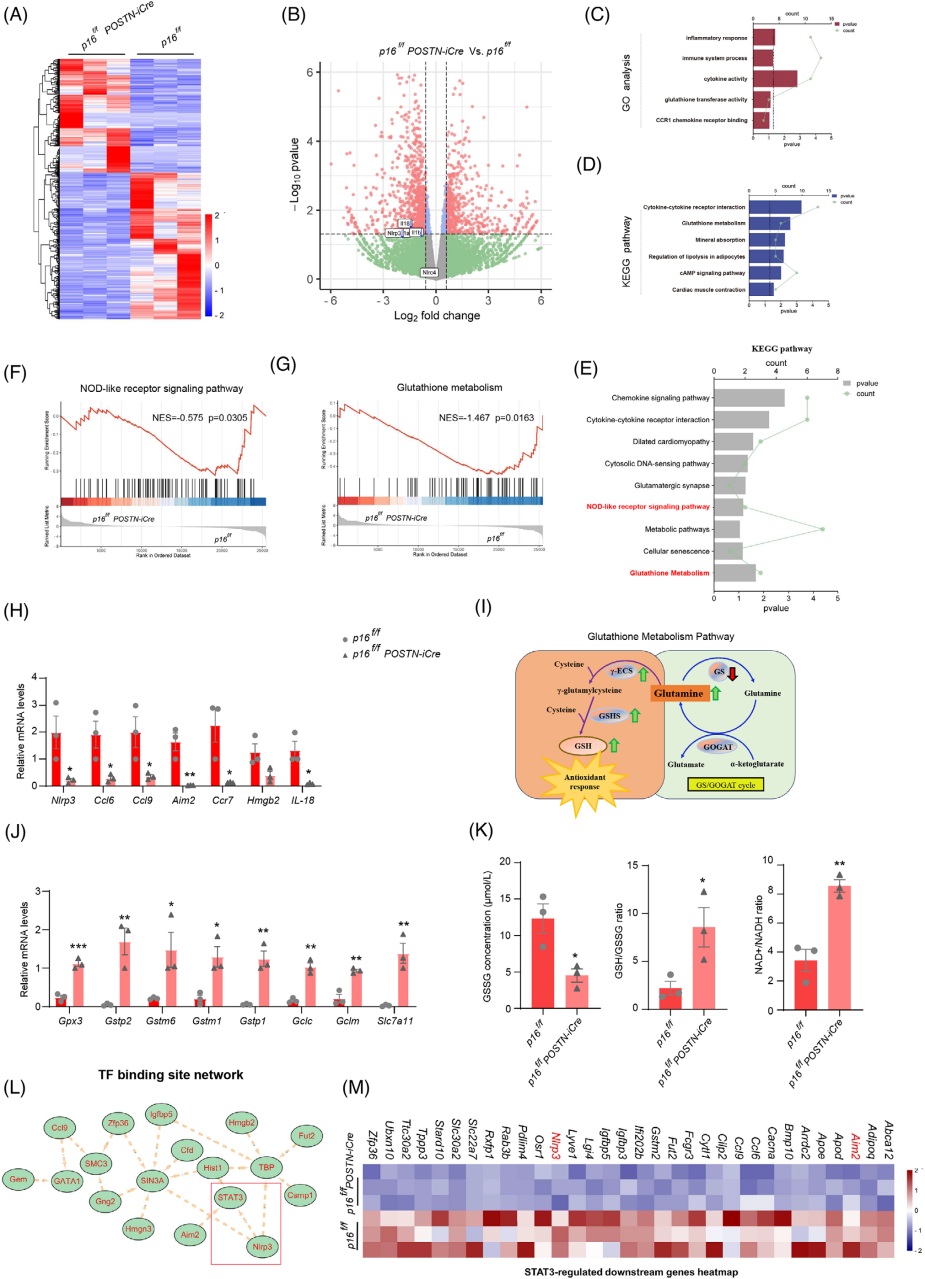
*POSTN*‐mediated *p16* deletion suppresses STAT3‐dependent NLRP3 inflammasome activation while enhancing glutathione metabolic pathway in fibroblasts. Following modelling in accordance with the mouse information and MI modelling method described in the legend of Figure 2 as mentioned previously, 13‐month‐old *p16^f/f^POSTN‐iCre* and *p16^f/f^* mice were humanely euthanised via intraperitoneal administration of pentobarbital sodium (100 mg/kg). RNA sequencing (RNA‐seq) was subsequently performed on primary fibroblasts isolated from the cardiac infarct zones of these animals. (A) Heat map of differentially expressed genes (DEGs). (B) Volcano plot of DEGs. (C, D) The GO and KEGG enrichment analysis were processed on total DEGs. (E) KEGG enrichment analysis was processed on downregulated DEGs in *p16^f/f^POSTN‐iCre* mice compared to *p16^f/f^* mice. (F, G) GSEA analysis showing NOD‐like receptor signalling pathway and glutathione metabolism. (H) Real‐time quantitative polymerase chain reaction (RT‐qPCR) of the above primary fibroblasts showing *Nlrp3*, *Ccl6*, *Ccl9*, *Aim2*, *Ccr7*, *Hmgb2* and *IL‐18*, and *Gapdh* was the loading control. (I) Diagram showing that glutathione metabolism pathway. (J) RT‐qPCR of the above primary fibroblasts showing *Gpx3*, *Gstp2*, *Gstm6*, *Gstm1*, *Gstp1*, *Gclc*, *Gclm*, and *Slc7a11*, and *Gapdh* was the loading control. (K) The above primary fibroblasts were examined for protein levels of GSSG (oxidised glutathione; µmol/L) and GSH (reduced glutathione; µmol/L), nicotinamide adenine dinucleotide including NAD⁺ (oxidised form; nmol/g) and NADH (reduced form; nmol/g) by ELISA assay. (L) Analysis of transcriptional factor binding site network with DEGs. (M) Heat map of STAT3‐regulated downstream genes. Three biological replicates were used per experiment. ^*^ *p* < .05, ^**^ *p* < .01, ^***^ *p* < .001 compared to *p16^f/f^* group, unpaired Student's *t*‐test.

To elucidate the functional consequences of *p16* deletion in fibroblasts within the infarcted myocardium post‐MI, primary cardiac fibroblasts were isolated from the infarcted myocardium of *p16^f/f^POSTN‐iCre* and *p16^f/f^* mice post‐MI and subjected to RNA‐seq analysis (Figure 3A). Our results showed that compared with *p16^f/f^* mice, *NLRP3*, *IL‐1β* and *IL‐18* were downregulated in fibroblasts of *p16^f/f^POSTN‐iCre* mice (Figure 3B). Gene Ontology (GO) and Kyoto Encyclopedia of Genes and Genomes (KEGG) pathway enrichment analyses of the entire set of differentially expressed genes (DEGs) demonstrated statistically significant enrichment in inflammatory responses, immune system processes, cytokine activities, glutathione transferase activities, CCR1 chemokine receptor binding, cytokine–cytokine receptor interactions, glutathione metabolism and cardiac muscle contraction pathways (Figure 3C,D). Further GSEA analysis and KEGG of downregulated genes in *p16^f/f^POSTN‐iCre* mice revealed significant enrichment in nucleotide‐binding oligomerization domain (NOD)‐like receptor signalling pathways and glutathione metabolism (Figure 3E–G). To observe the NOD‐like receptor signalling pathway, the mRNA levels of *Nlrp3*, *Ccl6*, *Ccl9*, *Aim2*, *Ccr7* and *IL‐18* were detected with real‐time quantitative PCR (RT‐qPCR) and found them reduced in fibroblasts of *p16^f/f^POSTN‐iCre* mice compared to *p16^f/f^* mice (Figure 3H). Moreover, to observe glutathione metabolism pathway, the mRNA levels of *Gpx3*, *Gstp2*, *Gstm6*, *Gstm1*, *Gstp1*, *Gclc*, *Gclm* and *Slc7a11* were detected with RT‐qPCR and found them elevated; the GSSG (oxidised glutathione) concentration was decreased, however, GSH (reduced glutathione)/GSSG ratio, and nicotinamide adenine dinucleotide including NAD⁺ (oxidised form)/NADH (reduced form) ratio were increased in fibroblasts of *p16^f/f^POSTN‐iCre* mice compared to *p16^f/f^* mice (Figure 3I–K). We further analysed transcriptional factor binding site network with DEGs and found that STAT3 might regulate *NLRP3* and *Aim2*, and further found that *p16* knockout in fibroblast downregulated STAT3‐mediated *NLRP3* and *Aim2* by STAT3‐regulated downstream genes analysis (Figure 3L,M), suggesting that *p16* knockout in fibroblast might downregulate STAT3‐mediated NLRP3 inflammasome.

### 3.4 *P16* overexpression promotes inflammageing and enhances STAT3‐dependent NLRP3 signalling in fibroblasts

To investigate whether *p16* overexpression induces inflammageing and activates NLRP3 signalling in fibroblasts, human cardiac fibroblasts (HCFBs) and human foetal lung fibroblasts (MRC‐5 cells) were transduced with a recombinant adenovirus overexpressing the *p16*. Our results showed that compared with the negative control (NC) group, the proportions of senescence‐associated‐β‐galactosidase (SA‐β‐gal)‐positive cells/areas; p16, p21, p53, p‐p65(Ser536)/p65, pro‐IL‐1β, IL‐1β, IL‐6, TNF‐α, NLRP3, ASC and Caspase‐1‐p40/‐p20 protein expression were increased significantly in *p16* overexpression group (Figure S1). To determine whether STAT3 mediates *p16* overexpression‐induced upregulation of NLRP3 and SASP genes in fibroblasts, we employed the potent, selective STAT3 inhibitor NSC74859 and *STAT3*‐specific siRNA. Results showed that NSC74859 and *STAT3* siRNA could reduce NLRP3 protein expression, and NSC74859 could reduce *IL‐1β*, *IL‐6*, *TNF‐α*, *IL‐18*, *Cxcl2*, *S100A8* and *S100A9* mRNA levels upregulated by *p16* overexpression in fibroblasts (Figure S2).

### 3.5 P16 interacts with STAT3 via its SH2 domain in fibroblasts

**FIGURE 4:**
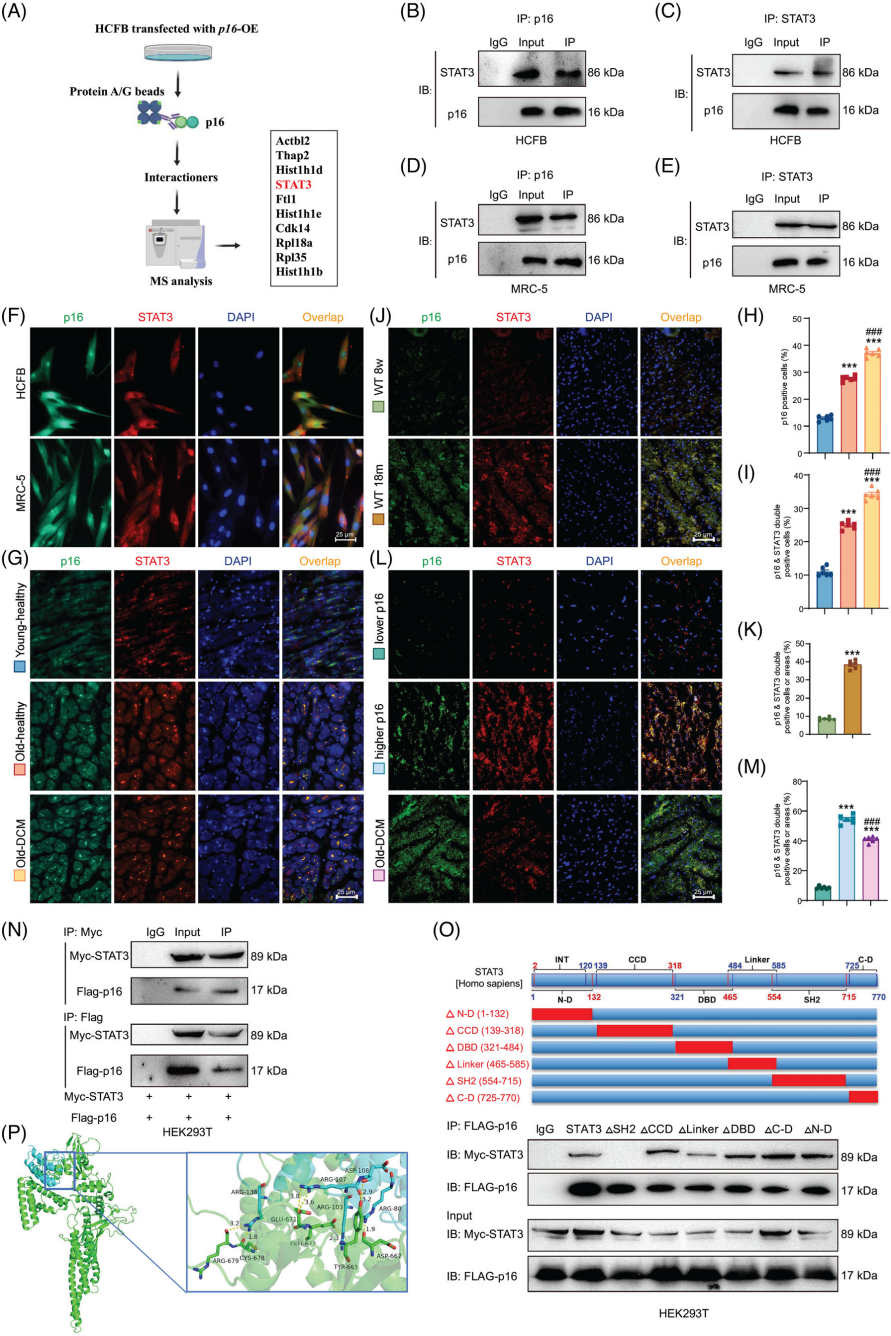
P16 interacts with STAT3 via its SH2 domain in fibroblasts. Human cardiac fibroblasts (HCFB cells) and human foetal lung fibroblasts (MRC‐5 cells) underwent transfection with adenovirus encoding *p16* overexpression constructs (*p16*‐OE). (A) Diagram showing proteins screen for interacting with p16 in *p16*‐OE‐HCFB cells by Mass spectrometry analysis. (B–E) These cells' proteins were extracted for anti‐p16 or anti‐STAT3 immunoprecipitation. Western blots were used for detecting p16 and STAT3. (F) Representative micrographs of HCFB and MRC‐5 cells stained for p16 and STAT3 with immunofluorescence. (G) Representative micrographs of hearts from young‐healthy people, old‐healthy people and old‐dilated cardiomyopathy (DCM) patients stained for p16 and STAT3 with immunofluorescence. (H, I) The percentage of cells or areas positive for p16 or double positive for p16 and STAT3 relative to total cells or areas. Values are mean ± standard error of the mean (SEM) from six determinations per group, ^***^ *p* < .001 compared with young‐healthy group; ^###^ *p* < .001 compared with old‐healthy group, one‐way analysis of variance (ANOVA) test. (J) Representative micrographs of hearts from 8‐week‐old and 18‐month‐old WT mice stained for p16 and STAT3 with immunofluorescence. (K) The percentage of cells or areas double positive for p16 and STAT3 relative to total cells or areas. Values are mean ± SEM from six determinations per group, ^***^ *p* < .001 compared with 8‐week‐old WT group, unpaired Student's *t*‐test. (L) Representative micrographs of hearts from lower‐p16‐expressed people, higher‐p16‐expressed people and DCM patients stained for p16 and STAT3 with immunofluorescence. (M) The percentage of cells or areas double positive for p16 and STAT3 relative to total cells or areas. Values are mean ± SEM from six determinations per group, ^***^ *p* < .001 compared to lower‐p16‐expressed group; ^###^ *p* < .001 compared to higher‐p16‐expressed group, one‐way ANOVA test. (N) HEK293T cells were co‐transfected with *Myc‐STAT3* plasmid and *Flag‐p16* overexpression adenovirus. Cells’ proteins were extracted for anti‐Myc or anti‐Flag immunoprecipitation. Western blots were used for detecting Myc and Flag. (O) HEK293T cells were co‐transfected with *Flag‐p16* (human) overexpression adenovirus and the plasmid of full length, SH2‐mutant (△SH2), CCD‐mutant (△CCD), Linker‐mutant (△Linker), DBD‐mutant (△DBD), C‐D‐mutant (△C‐D) or N‐D‐mutant (△N‐D) for human STAT3. Western blots were used for detecting Myc and Flag. (P) The protein docking domain of p16 and STAT3 was performed via the HDOCK server.

To elucidate the mechanism of *p16* overexpression‐induced NLRP3 upregulation and identify p16‐interacting proteins, HCFB cells were transduced with *p16*‐overexpressing adenovirus, followed by anti‐p16 immunoprecipitation of cell lysates and subsequent mass spectrometry analysis. Results showed that p16 could bind to STAT3 (Figure 4A). Our results further showed that p16 bound STAT3 in both cytoplasm and nuclei of HCFB and MRC‐5 cells (Figure 4B–F).

To assess whether ageing and dilated cardiomyopathy (DCM) potentiate p16‐STAT3 interaction, our comparative analysis revealed that young‐healthy controls exhibited a significantly lower abundance of p16‐positive cells and p16/STAT3 co‐expressing cells compared to old‐healthy individuals, whereas old‐DCM patients showed marked increases in these populations (Figure 4G–I). Compared with 8‐week‐old WT mice, a significant increase of p16 and STAT3 double positive areas or cells was shown in 18‐month‐old WT mice (Figure 4J,K). Compared with higher‐p16‐expressed people, a significant decrease was shown in the p16 and STAT3 double positive areas or cells in lower‐p16‐expressed people, however, a significant increase of p16 and STAT3 double positive areas or cells was shown in DCM patients (Figure 4L,M).

To map the interacting domains between p16 and STAT3, we performed co‐immunoprecipitation assays in HEK293T cells. The cells were transduced with an adenovirus vector overexpressing Flag‐tagged *p16* and co‐transfected with plasmids encoding full‐length human *STAT3* or its domain‐specific deletion mutants: N‐terminal domain‐deleted mutant (ΔN‐D), coiled‐coil domain‐deleted mutant (ΔCCD), DNA‐binding domain‐deleted mutant (ΔDBD), linker region‐deleted mutant (ΔLinker), SH2 domain‐deleted mutant (ΔSH2) and C‐terminal domain‐deleted mutant (ΔC‐D). We found that p16 bound STAT3, and the interaction between STAT3 and p16 disappeared after mutating SH2 domain, while the mutations of other domains had no significant effect on the interaction between p16 and STAT3 in HEK293T cells (Figure 4N,O). These findings demonstrate that the specific interaction between p16 and STAT3 is mediated through the SH2 domain of STAT3. The protein docking result revealed the putative binding amino acid residues within the interaction domains of p16 and STAT3 (Figure 4P).

### 3.6 *P16* overexpression enhances EZH2‐STAT3 interaction, promoting EZH2‐mediated STAT3 K49 di‐methylation and Y705 phosphorylation in fibroblasts

**FIGURE 5:**
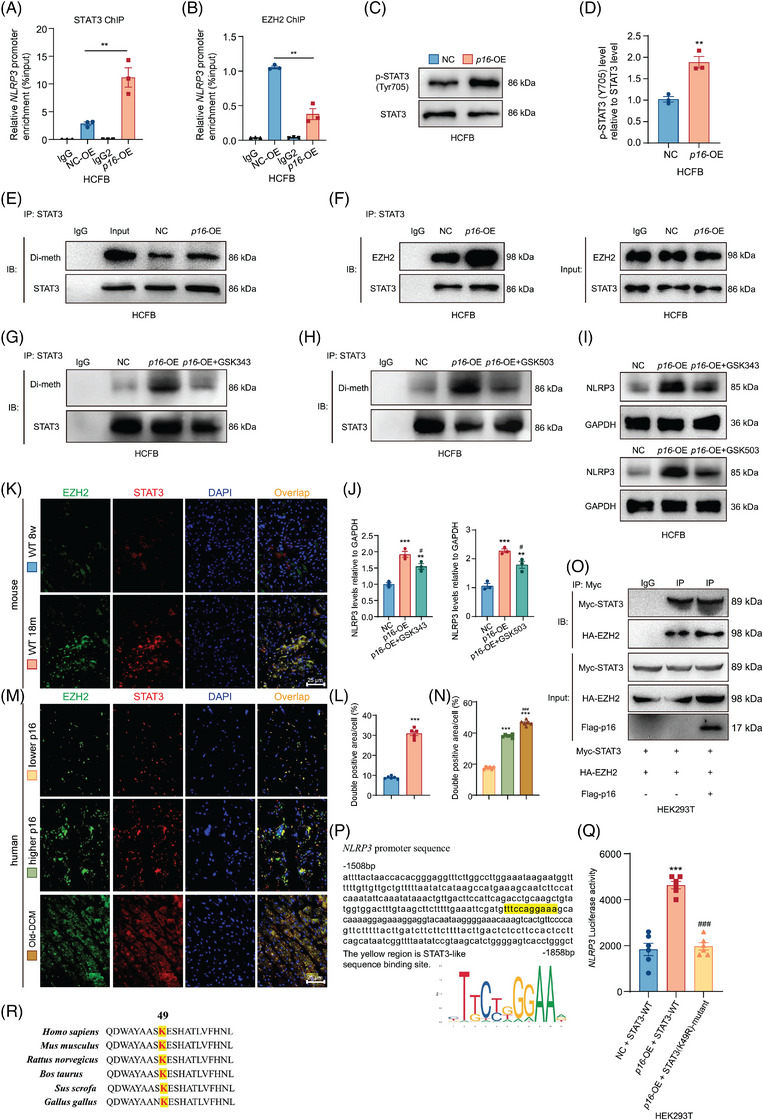
P16 promotes the interaction between EZH2 and STAT3, and increases the di‐methylation on K49 and phosphorylation on Y705 of STAT3 by EZH2 in human cardiac fibroblast (HCFB) cells. HCFB cells were transfected with *p16* overexpression (*p16*‐OE) adenovirus. (A, B) The binding levels of STAT3 or EZH2 in *NLRP3* promoter region of HCFB cells were detected by chromatin immunoprecipitation (ChIP) assay. (C) Western blots of cells extract showing p‐STAT3(Tyr705), STAT3 was the loading control. (D) Protein levels relative to negative control (NC) group were assessed by densitometric analysis. Three biological replicates were used per experiment. ^**^ *p* < .01 compared to NC group, unpaired Student's *t*‐test. (E) HCFB cells proteins were extracted for anti‐STAT3 immunoprecipitation. Western blots were used for detecting di‐methylation level and STAT3. (F) HCFB cells proteins were extracted for anti‐STAT3 immunoprecipitation. Western blots were used for detecting EZH2 and STAT3. (G, H) HCFB cells were transfected with *p16* overexpression (*p16*‐OE) adenovirus and treated with EZH2 inhibitors GSK343 or GSK503, targeting histone‐lysine N‐methyltransferase activity. HCFB cells proteins were extracted for anti‐STAT3 immunoprecipitation. Western blots were used for detecting di‐methylation level and STAT3. (I) Western blots were used for detecting NLRP3, GAPDH was the loading control. (J) Protein levels relative to NC group were assessed by densitometric analysis. Three biological replicates were used per experiment. ^**^ *p* < .01, ^***^ *p* < .001 compared to NC group; ^#^ *p* < .05 compared to *p16*‐OE group, one‐way analysis of variance (ANOVA) test. (K) Representative micrographs of hearts from 8‐week‐old and 18‐month‐old WT mice stained for EZH2 and STAT3 with immunofluorescence. (L) The percentage of cells or areas double positive for EZH2 and STAT3 relative to total cells or areas. Values are means ± standard error of the mean (SEM) of six determinations. ^***^ *p* < .001 compared to 8‐week‐old WT group, unpaired Student's *t*‐test. (M) Human myocardial tissues from healthy and dilated cardiomyopathy (DCM) patients were examined for p16 protein levels by ELISA assay. Representative micrographs of hearts from lower‐p16‐expressed people, higher‐p16‐expressed people and DCM patients stained for EZH2 and STAT3 with immunofluorescence. (N) HEK293T cells were co‐transfected with *Myc‐STAT3* plasmid, *HA‐EZH2* plasmid and/or *Flag‐p16* overexpression adenovirus. Cells' proteins were extracted for anti‐Myc immunoprecipitation. Western blots were used for detecting Myc, HA and Flag. (O) STAT3‐like sequence binding site (highlighted with yellow) in *NLRP3* promoter region. (P) The mutant plasmid (K to R mutation, K49R) of the K49 site or WT plasmid of STAT3 was constructed and transfected into HEK293T cells. *NLRP3* promoter activity was measured by a luciferase reporter gene assay. Three biological replicates were used per experiment. Values are means ± SEM of six determinations. ^***^ *p* < .001 compared to NC group; ^###^ *p* < .001 compared to *p16*‐OE group, one‐way ANOVA test. (Q) Homology analysis of STAT3 locus 49 among different species.

To clarify whether *p16* overexpression enhances STAT3 transcriptional activity, we found that compared to NC cells, *p16* overexpression promoted STAT3 binding to the *NLRP3* promoter while inhibiting EZH2 occupancy at this locus in both HCFB and MRC‐5 cells detected with chromatin immunoprecipitation (ChIP) assay (Figures 5A,B and S3A,B). To assess whether *p16* overexpression enhances STAT3 transcriptional activity via Y705 phosphorylation, we observed that compared to NC cells, *p16* overexpression elevated p‐STAT3(Y705) levels in both HCFB and MRC‐5 cells (Figures 5C,D and S3C,D).

Emerging evidence suggests that EZH2 promotes STAT3 Y705 phosphorylation through STAT3 di‐methylating.^25^ Here, using quantitative immunoprecipitation, we measured STAT3 di‐methylation levels in HCFB and MRC‐5 cells and found *p16* overexpression increased STAT3 di‐methylation compared to NC cells (Figures 5E and S3E). To observe whether *p16* overexpression enhances EZH2‐STAT3 interaction for STAT3 di‐methylation, we found *p16* overexpression increased EZH2‐STAT3 binding (Figures 5F and S3F). Inhibiting EZH2 histone‐lysine N‐methyltransferase activity with GSK343 or GSK503 abolished *p16* overexpression‐induced STAT3 di‐methylation and NLRP3 expression, indicating that *p16* overexpression enhances EZH2‐mediated STAT3 di‐methylation to promote NLRP3 expression (Figure 5G–J). Compared with 8‐week‐old WT mice, a significant increase of EZH2 and STAT3 double positive areas or cells was shown in 18‐month‐old WT mice (Figure 5K,L). Compared with higher‐p16‐expressed people, a significant decrease was shown in the EZH2 and STAT3 double positive areas or cells in lower‐p16‐expressed people, however, a significant increase of EZH2 and STAT3 double positive areas or cells was shown in DCM patients (Figure 5M,N). In HEK293T cells, *p16* overexpression could also enhance the interaction of exotic EZH2 and STAT3 (Figure 5O).

Prior research demonstrated that EZH2‐mediated STAT3 di‐methylation occurs at the K49 residue.^26^ To assess whether p16‐regulated STAT3 transcriptional activity requires the K49 residue, we generated a K49R mutant (lysine to arginine substitution) and transfected it into HEK293T cells for luciferase reporter assays. *P16* overexpression upregulated *NLRP3* luciferase activity compared to the NC group. STAT3‐K49R treatment downregulated *NLRP3* luciferase activity in *p16*‐overexpressed cells compared to the *p16* overexpression group (Figure 5P–Q). Additionally, cross‐species alignment revealed high conservation of the STAT3 K49 residue (Figure 5R). However, EZH2‐STAT3 binding was unaffected by the K49 mutation. The STAT3 K49 mutation did not disrupt p16‐STAT3 interaction (Figure S4A,B).

### 3.7 *P16* overexpression enhances *NLRP3* transcription by modulating histone modifications and disrupting Bmi‐1‐EZH2/BCL‐6 complex formation at the *NLRP3* promoter in fibroblasts

**FIGURE 6:**
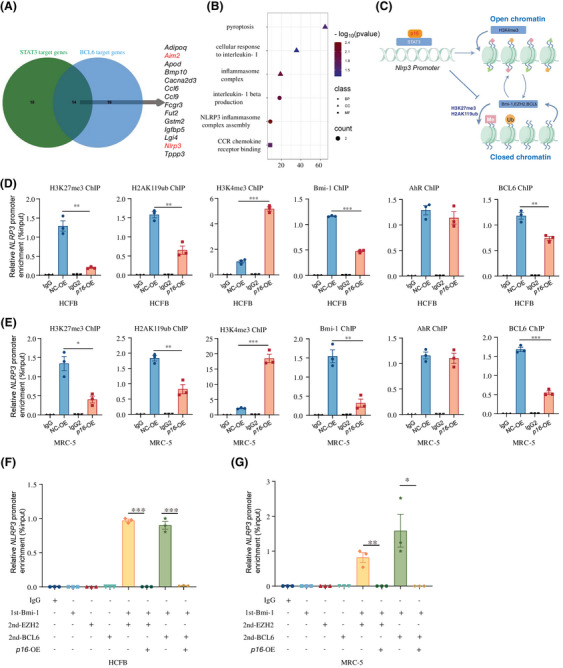
*P16* overexpression enhances *NLRP3* transcription by modulating histone modifications and disrupting Bmi‐1‐EZH2/BCL6 complex formation at the *NLRP3* promoter in fibroblasts. Following myocardial infarction (MI), *p16^f/f^POSTN‐iCre* and *p16^f/f^* mice were humanely euthanised via intraperitoneal injection of pentobarbital sodium (100 mg/kg). (A) The primary fibroblasts from the infarcted areas in hearts of these mice were detected with RNA‐seq, and differentially expressed genes (DEGs) were processed to analyse the target gene numbers of transcriptional factor STAT3 or BCL6 with Venn diagram. (B) KEGG enrichment analysis of co‐targeted gene by STAT3 and BCL6. Human cardiac fibroblast (HCFB) and MRC‐5 cells were transfected with *p16* overexpression (*p16*‐OE) adenovirus. (C) Diagram showing that the binding level of related proteins about the histone modification to the *NLRP3* promoter region for regulating chromatin open or closed. (D, E) The histone modification level of H3K27me3, H2AK119ub and H3K4me3 and the binding levels of Bmi‐1, AhR and BCL6 in *NLRP3* promoter region and in HCFB and MRC‐5 cells were detected by chromatin immunoprecipitation (ChIP) assay. Three biological replicates were used per experiment. ^*^ *p* < .05, ^**^ *p* < .01, ^***^ *p* < .001 compared to negative control (NC) group, unpaired Student's *t*‐test. (F, G) The binding levels of Bmi‐1‐EZH2 complex or Bmi‐1‐BCL6 complex in the promoter regions of *NLRP3* in HCFB and MRC‐5 cells were detected by Re‐ChIP assay. Three biological replicates were used per experiment. ^*^ *p* < .05, ^**^ *p* < .01, ^***^ *p* < .001, one‐way analysis of variance (ANOVA) test.

STAT3 is known to activate downstream targets by directly binding their promoters, or indirectly through disrupting histone‐modifying protein interactions at these regulatory regions.^31^ The primary fibroblasts from *p16^f/f^POSTN‐iCre* and *p16^f/f^* mice following MI were detected with RNA‐seq (Figure 3A), and DEGs were processed to analyse the target genes of transcriptional factor STAT3 or BCL6. Results showed that 14 genes were regulated by STAT3 and BCL6 simultaneously including *Nlrp3* and *Aim2* and so forth and NLRP3 inflammasome signalling was enriched (Figure 6A,B). To determine whether the complex of p16 and STAT3 could affect the histone modification of *NLRP3* promoter region, our results showed that *p16* overexpression could increase the combined active H3K4me3 and decrease the combined repressive H3K27me3 and H2AK119ub to *NLRP3* promoter region compared with NC group (Figure 6C–E). Furthermore, the binding level of related proteins about the above histone modification to the *NLRP3* promoter region was detected and found that *p16* overexpression could significantly weaken the binding of Bmi‐1 and BCL6 to *NLRP3* promoter region compared to NC group (Figure 6C–E).

To determine whether *p16* could affect the formation of Bmi‐1‐EZH2 or Bmi‐1‐BCL6 complex in the *NLRP3* promoter region, HCFB and MRC‐5 cells were transduced with a *p16*‐overexpressing adenovirus vector, followed by Re‐ChIP analysis to assess protein–DNA interactions. Results showed that *p16* overexpression could interfere the formation of Bmi‐1‐EZH2 or Bmi‐1‐BCL6 complex in the *NLRP3* promoter region (Figure 6F,G).

### 3.8 P16 modulates STAT3‐regulated senescence‐associated genes in fibroblasts

To evaluate p16's modulation of STAT3‐regulated senescence pathways, we examined five STAT3 transcriptional targets^32^ implicated in cellular senescence: *TNFRSF1*, *Myc*, *BIRC3*, *NAMPT* and *NNMT*. Our results showed that p16 could promote the binding level of STAT3 in *NNMT* promoter and decrease the binding level of *NAMPT*, but had no significant effect on *TNFRSF1*, *Myc* and *BIRC3* in HCFB cells; p16 could decrease the binding level of *Myc*, *BIRC3* and *NAMPT*, but had no significant effect on *TNFRSF1* and *NNMT* in MRC‐5 cells (Figure S5A,C).

To determine whether *p16* could affect the formation of Bmi‐1‐EZH2 or Bmi‐1‐BCL6 complex in the *TNFRSF1*, *Myc*, *BIRC3*, *NAMPT* or *NNMT* promoter region, HCFB and MRC‐5 cells were transfected with *p16* overexpression adenovirus and conducted Re‐ChIP assay. In HCFB cells, *p16* overexpression could interfere the formation of Bmi‐1‐EZH2 or Bmi‐1‐BCL6 complex in the *TNFRSF1*, *Myc* and *NNMT* promoter region. In MRC‐5 cells, *p16* overexpression could interfere the formation of Bmi‐1‐EZH2 or Bmi‐1‐BCL6 complex in the *TNFRSF1*, *BIRC3*, *NAMPT* or *NNMT* promoter region (Figure S5B,D).

### 3.9 P16‐accumulated ageing cardiac fibroblasts or *p16* overexpression adenovirus injection exacerbated NLRP3 signalling activation, fibrosis and inflammation in the MI area

**FIGURE 7:**
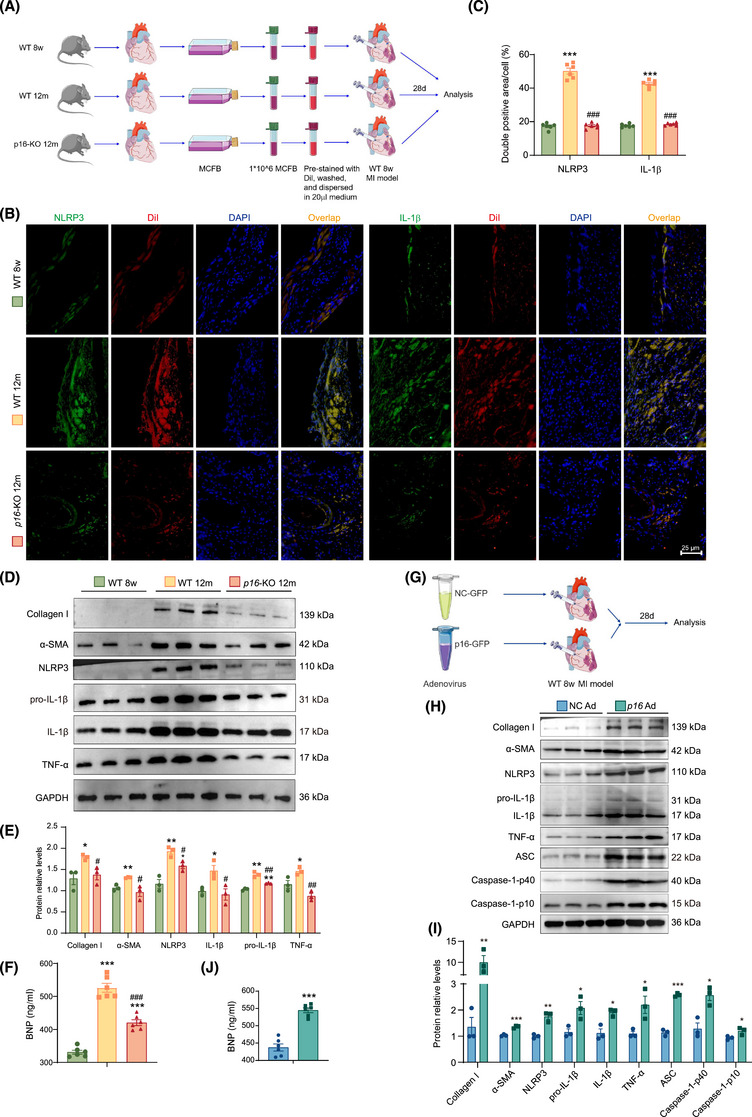
P16‐accumulated ageing cardiac fibroblasts or *p16* overexpression adenovirus injection exacerbated NLRP3 signalling activation, fibrosis and inflammation in the myocardial infarction (MI) area. The 8‐week‐old WT mice, 12‐month‐old *p16*‐KO and 12‐month‐old WT mice were euthanised with pentobarbital sodium (100 mg/kg) intraperitoneally. (A) Mouse cardiac fibroblasts (MCFBs) were isolated from them, labelled with DiI Stain, and then transplanted into the infarcted and peripheral areas of 8‐week‐old MI mice. Four weeks later, the levels of profibrotic and proinflammatory factors were detected. (B) Representative micrographs of hearts for NLRP3 and IL‐1β with immunofluorescence merged with DiI Stain. (C) The percentage of cells or areas for NLRP3 and DiI or IL‐1β and DiI double positive relative to total cells or areas. (D) Western blots of tissues extract showing Collagen I, α‐SMA, NLRP3, pro‐IL‐1β, IL‐1β and TNF‐α in infarcted area of mice. GAPDH was the loading control. (E) Protein levels were assessed by densitometric analysis. (F) Serum examined for BNP protein levels (ng/mL) by ELISA assay. The experiments were performed with six mice per group. ^*^ *p* < .05, ^**^ *p* < .01, ^***^ *p* < .001 compared to MCFB from 8‐week‐old WT mice; ^#^ *p* < .05, ^##^ *p* < .01, ^###^ *p* < .001 compared to MCFB from 12‐month‐old WT mice, one‐way analysis of variance (ANOVA) test. (G) *P16* overexpression (*p16* Ad) or negative control adenovirus (NC Ad) was injected into the infarcted and peripheral areas of 8‐week‐old MI mice. Four weeks later, the levels of profibrotic and proinflammatory factors were detected. (H) Western blots of the myocardial tissue extract in the infarcted area showing Collagen I, α‐SMA, NLRP3, pro‐IL‐1β, IL‐1β, TNF‐α, ASC, Caspase‐1‐p40 and Caspase‐1‐p10 in infarcted area of mice. GAPDH was the loading control. (I) Protein levels were assessed by densitometric analysis. (J) Serum examined for BNP protein levels (ng/mL) by ELISA assay. The experiments were performed with six mice per group. ^*^ *p* < .05, ^**^ *p* < .01, ^***^ *p* < .001 compared to NC‐adenovirus treatment group, unpaired Student's *t*‐test.

To elucidate the critical role of p16‐accumulated ageing cardiac fibroblasts in ventricular remodelling following MI, mouse cardiac fibroblasts (MCFBs) were isolated from 8‐week‐old WT, 12‐month‐old *p16*‐KO and WT mice, labelled with DiI stain, and then transplanted into the infarcted and peripheral areas of 8‐week‐old MI mice (Figure 7A). Four weeks later, the levels of profibrotic and proinflammatory factors were detected. Compared to MCFB from 8‐week‐old WT mice and 12‐month‐old *p16*‐KO mice, MCFB from 12‐month‐old WT mice were transplanted and produced higher levels of NLRP3 and IL‐1β in the infarcted and peripheral areas of 8‐week‐old MI mice. Obviously elevated protein levels of Collagen I, α‐SMA, NLRP3, pro‐IL‐1β, IL‐1β and TNF‐α were observed in both the infarcted myocardial tissue and peri‐infarct regions, and in the secreted protein levels of BNP in serum in MCFB from 12‐month‐old WT mice in comparison with MCFB from 8‐week‐old WT mice and 12‐month‐old *p16*‐KO mice (Figure 7B–F).

To further confirm the effect of p16 accumulation on proinflammation and profibrosis in MI area, *p16* overexpression adenovirus was injected into the infarcted and peripheral areas of 8‐week‐old MI mice (Figure 7G). Four weeks later, the levels of profibrotic and proinflammatory factors were detected. Compared to NC adenovirus treatment group, in the *p16*‐overexpressing adenovirus‐treated group, we observed significantly elevated protein expression of Collagen I, α‐SMA, NLRP3, pro‐IL‐1β, IL‐1β, TNF‐α, ASC, Caspase‐1‐p40 and Caspase‐1‐p10 within the infarct zone, accompanied by marked upregulation of serum BNP secretion (Figure 7H–J).

To observe whether *p16* overexpression could aggravate NLRP3 signalling activation in MI mice, our results showed that *p16* overexpression obviously increased the positive areas or cells of NLRP3 and IL‐1β. To further investigate whether p16 accumulation could exacerbate the NLRP3 signalling activation in human, our results showed that compared with higher‐p16‐expressed people, a significant reduction in NLRP3‐ and IL‐1β‐positive areas/cells was observed in individuals with low p16 expression; conversely, a marked elevation of these inflammatory markers was detected in DCM patients (Figure S6A–D).

### 3.10 FNLM‐*p16*‐siRNA: Characterisation and in vitro targeting efficacy

To achieve p16 targeting in post‐MI cardiac fibroblasts, we developed FNLM‐*p16*‐siRNA based on established protocols.^29^ First, calcium silicate‐based MSNs were synthesised to encapsulate *p16*‐siRNA. Liposomes were prepared via thin‐film hydration and coated onto MSNs‐*p16*‐siRNA through sequential extrusion using decreasing pore‐size membranes. FH peptide was conjugated to the polyethylene glycol (PEG) terminus of lipids for Tenascin‐C targeting. NMPs were purified using an established protocol^33^ and incorporated prior to extrusion at a 1:4 protein‐to‐lipid ratio (Figure S7A). The successful encapsulation of FNLM‐*p16*‐siRNA was verified via transmission electron microscopy, which revealed a distinct lipid bilayer surrounding the dark magnetic silica nanocore (Figure S7B). The formulation demonstrated colloidal stability in phosphate‐buffered saline (PBS) for a minimum duration of 1 week. Complementary characterisation through zeta potential measurements showed a significant surface charge reversal post‐cationic lipid coating, confirming effective nanoparticle surface modification. For assessing siRNA release kinetics, the nanocarrier was cultured in PBS containing 10% foetal bovine serum at 37°C over defined intervals. Gradual release of *p16*‐siRNA was observed in a time‐dependent manner. Dynamic light scattering analysis further corroborated successful lipid coating by demonstrating increased hydrodynamic diameter of nanoparticles, exhibiting a mean diameter of 175 nm (Figure S7C). To further clarify the targeting of the nanoparticle to cardiac fibroblasts, cardiac fibroblasts were incubated with FNLM‐*p16*‐siRNA after 24 h of hypoxia. Immunofluorescence showed that with the help of FH peptide, FNLM‐*p16*‐siRNA could successfully enter cardiac fibroblasts (Figure S7D).

### 3.11 In vivo biodistribution of FNLM‐*p16*‐siRNA in post‐MI murine models

To further determine the targeting and localisation of FNLM‐*p16*‐siRNA in vivo, FNLM‐*p16*‐siRNA was injected into 12‐month‐old mice by tail vein once a week for 4 weeks following MI. Then, immunofluorescence staining was used to position the FNLM‐*p16*‐siRNA in organs of 13‐month‐old mice and the result showed there was a large amount of nanoparticle in the infarcted area, while no significant nanoparticles infiltration was found in the non‐infarcted area of the heart (Figure S8A). At the same time, we also detected the localisation of the nanoparticles in the liver, lung, kidney and spleen of mice by immunofluorescence staining. Results revealed preferential nanoparticle accumulation in the liver and kidneys, potentially reflecting in vivo metabolic processing (Figure S8B).

To further evaluate the toxic effects of nanoparticles both in vitro, we incubated cardiac fibroblasts and cardiomyocytes with nanoparticles. FNLM‐NC‐siRNA or FNLM‐*p16*‐siRNA treatment had no significant effect on the growth of MCFBs when observed under microscope (Figure S9A). Cell proliferation of cardiac fibroblasts and cardiomyocytes was detected by Cell Counting Kit‐8. Cellular proliferation assays demonstrated that treatment with either FNLM‐NC‐siRNA or FNLM‐*p16*‐siRNA did not significantly alter cell growth dynamics across all evaluated time points (24, 48 and 72 h) compared to the untreated control group (Figure S9B). In vivo, histopathological examination using haematoxylin and eosin (H&E) staining revealed no significant pathological alterations or inflammatory infiltration in the liver, lung, kidney and spleen tissues of mice administered with FNLM‐NC‐siRNA or FNLM‐*p16*‐siRNA, comparable to the control group (Figure S9C). Biochemical analysis revealed that serum concentrations of alanine transaminase (ALT) and aspartate transaminase (AST) in murine models administered with FNLM‐NC‐siRNA or FNLM‐*p16*‐siRNA remained comparable to those in the control group, with no significant fluctuations observed. These findings indicate an absence of substantial hepatotoxicity in vivo for both treatment groups (Figure S9D,E). At the same time, the serum urea and creatinine levels in FNLM‐NC‐siRNA or FNLM‐*p16*‐siRNA treatment groups also exhibits no significant changes compared with the control group, suggesting that they had no obvious nephrotoxicity in vivo (Figure S9F,G). The results showed that we developed a post‐MI cardiac fibroblast‐targeting nanoparticle with demonstrated biocompatibility across in vitro and in vivo experimental models.

### 3.12 FNLM‐*p16*‐siRNA attenuates post‐MI myocardial fibrosis and inflammation

**FIGURE 8:**
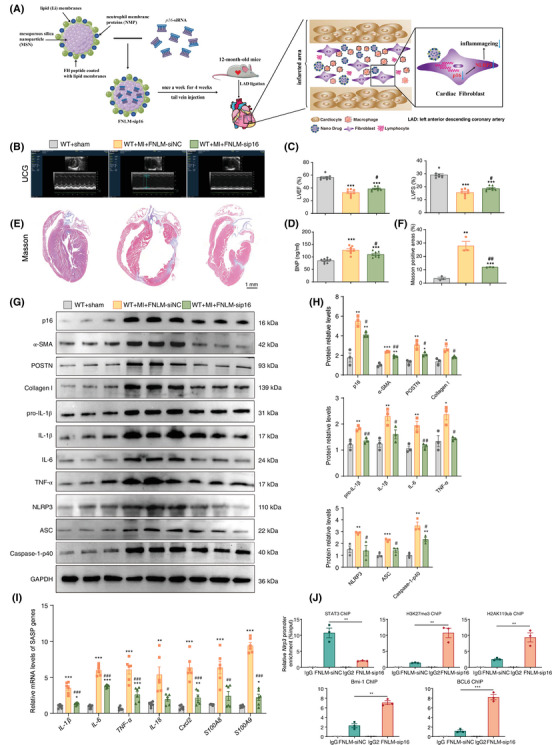
FNLM‐*p16*‐siRNA attenuates post‐myocardial infarction (MI) myocardial fibrosis and inflammation. Following MI, 12‐month‐old mice received weekly tail vein injections of FNLM‐*p16*‐siRNA for 4 consecutive weeks. Subsequent analyses were performed using 13‐month‐old MI mice at the experimental endpoint. (A) Diagram showing that the nanoparticles FNLM‐*p16*‐siRNA (FNLM‐sip16) or FNLM‐NC‐siRNA (FNLM‐siNC) were established and injected into them through tail vein once a week for 4 weeks. (B) Mice were anaesthetised by inhaling isoflurane at a 1:1 concentration with oxygen (4% induction concentration, 1.5% maintenance concentration) and detected by Color Doppler echocardiography. (C) Left ventricular ejection fraction (LVEF) and left ventricular shortening fraction (LVFS). (D) Level of BNP (ng/mL) in serum. (E) Representative micrographs of paraffin‐embedded heart ventricular wall sections of mice stained for Masson's trichrome (Masson) staining. (F) The percentage of cells or areas positive for Masson's trichrome‐labelled interstitial fibres. (G, H) Protein levels and statistical analysis of p16, α‐SMA, POSTN, Collagen I, pro‐IL‐1β, IL‐1β, IL‐6, TNF‐α, NLRP3, ASC and Caspase‐1‐p40, in the myocardial tissue of the infarcted area by Western blots detection. GAPDH was the loading control. Eight mice per group were used for experiments. (I) Real‐time quantitative polymerase chain reaction (RT‐qPCR) of myocardial tissue extract of the infarcted area showing *IL‐1β*, *IL‐6*, *TNF‐α*, *IL‐18*, *Cxcl2*, *S100A8* and *S100A9*, and *Gapdh* was the loading control. Values are means ± standard error of the mean (SEM) of six determinations. ^*^ *p* < .05, ^**^ *p* < .01, ^***^ *p* < .001 compared with WT + sham mice, ^#^ *p* < .05, ^##^ *p* < .01, ^###^ *p* < .001 compared with WT + MI + FNLM‐siNC mice, one‐way analysis of variance (ANOVA) test. (J) The binding levels of STAT3, H2AK119ub, H3K27me3, Bmi‐1 and BCL6 in the *Nlrp3* promoter region in hearts from FNLM‐siNC‐treated mice and FNLM‐sip16‐treated mice were detected by chromatin immunoprecipitation (ChIP). Three biological replicates were used per experiment. ^**^ *p* < .01, ^***^ *p* < .001 compared with FNLM‐siNC‐treated mice, unpaired Student's *t*‐test.

FNLM‐*p16*‐siRNA was administered via tail vein injection to 12‐month‐old mice once weekly for 4 weeks post‐MI, with subsequent functional and histological assessments performed at 13 months (Figure 8A). Treatment significantly improved cardiac function, as evidenced by elevated LVEF and LVFS compared to FNLM‐NC‐siRNA controls (Figure 8B,C). Circulating BNP levels, a biomarker of heart failure, were correspondingly reduced in FNLM‐*p16*‐siRNA‐treated animals (Figure 8D). Histological analysis revealed attenuated myocardial fibrosis in treated mice, with reduced Masson's trichrome‐positive areas (Figure 8E,F). Western blot and immunohistochemical staining confirmed effective *p16* knockdown in the infarct zone, demonstrating decreased p16 protein expression (Figure 8G,H) and reduced p16‐positive cell populations (Figure S10A,B). Collectively, these findings establish FNLM‐*p16*‐siRNA as a promising therapeutic intervention for post‐MI cardiac dysfunction and fibrosis.

To evaluate the impact of FNLM‐*p16*‐siRNA on post‐MI ventricular remodelling, we quantified fibrotic markers α‐SMA, POSTN and Collagen I via Western blot. FNLM‐*p16*‐siRNA‐treated mice exhibited significantly reduced protein levels of these markers compared to controls (Figure 8G,H). Consistent results were observed in immunohistochemical analyses of α‐SMA, POSTN and Collagen I deposition (Figure S10C,D), with corresponding decreases in *Col1α1* and *Postn* mRNA expression (Figure S10E). Collectively, these data demonstrate that FNLM‐*p16*‐siRNA treatment attenuates myocardial fibrosis in the infarct zone following MI.

To assess inflammageing attenuation in the infarcted myocardium of FNLM‐*p16*‐siRNA‐treated mice, Western blot or RT‐qPCR analysis was performed to quantify SASP components. FNLM‐*p16*‐siRNA significantly reduced mRNA levels and protein levels of IL‐1β, IL‐6, TNF‐α and mRNA levels of *IL‐18*, *Cxcl2*, *S100A8* and *S100A9* compared to FNLM‐NC‐siRNA controls (Figure 8G–I). Consistently, immunohistochemistry revealed diminished IL‐1β, IL‐6, TNF‐α and phosphorylated p65(S536)‐positive areas in the FNLM‐*p16*‐siRNA‐treated mice (Figure S10F,G). To investigate the FNLM‐*p16*‐siRNA‐mediated attenuation of ventricular remodelling post‐MI, we focused on the NLRP3 inflammasome signalling pathway. RT‐qPCR analysis revealed significant downregulation of *Nlrp3* mRNA levels following FNLM‐*p16*‐siRNA treatment (Figure S10E). Western blotting further demonstrated reduced expression levels of NLRP3, ASC and Caspase‐1‐p40 proteins in the intervention group compared to NC controls (Figure 8G,H). Immunohistochemical analysis of infarcted myocardium confirmed suppression of NLRP3 pathway activation with FNLM‐*p16*‐siRNA intervention (Figure S10H,I).

To assess whether p16 enhances *NLRP3* transcription by suppressing Bmi‐1‐BCL6 complex association with the *NLRP3* promoter, we performed targeted ChIP assays. In MI regions, FNLM‐*p16*‐siRNA treatment significantly reduced STAT3 occupancy at the *NLRP3* promoter while elevating repressive histone marks H2AK119ub and H3K27me3. Concurrently, siRNA‐mediated *p16* knockdown augmented Bmi‐1‐BCL6 complex recruitment, as evidenced by increased Bmi‐1 and BCL6 binding at the *NLRP3* promoter locus (Figure 8J). These results suggested that FNLM‐*p16*‐siRNA treatment enhances the binding of Bmi‐1‐BCL6 complex to *NLRP3* promoter region.

## 4 DISCUSSION

Senescent cell accumulation and SASP secretion correlate with age‐related cardiac pathologies (heart failure, myocardial ischaemia, infarction).^34^ Cardiac fibroblasts, activated by endogenous/exogenous stressors (oxidative radicals, DAMPs, chronic inflammation), differentiate into myofibroblasts that secrete profibrotic proteins (Collagen I, α‐SMA), driving fibrosis.^13^, ^35^, ^36^, ^37^ Chronic inflammation, in particular, is a pivotal activator of this fibrotic phenotype.^38^ IL‐1β antagonism represents a critical therapeutic strategy in cardiovascular disease.^39^ In non‐ST‐segment elevation MI patients, IL‐1β antagonism emerges as a tractable therapeutic target for mitigating inflammatory biomarker dysregulation.^40^ Emerging evidence has shifted focus from the cellular origins of inflammation to senescent cell‐derived cytokines as critical mediators of chronic inflammation in age‐related pathologies, with recent studies highlighting their pathogenic role through SASP modulation.^11^, ^12^, ^41^ Despite its established role as a critical senescence regulator, the functional involvement of p16 protein in modulating cardiac fibroblast SASP following MI remains poorly characterised. Our clinical findings demonstrate a positive correlation between p16‐driven inflammageing and the extent of ventricular remodelling following MI in patients. *POSTN*‐*iCre* was utilised to target myofibroblast‐specific functions during post‐infarction ventricular remodelling, given that POSTN‐positive fibroblasts constitute a distinct cardiac fibroblast subset.^42^, ^43^, ^44^ In this study, *POSTN*‐driven *p16* deletion was found to enhance cardiac function and attenuate post‐infarction ventricular remodelling in MI mice, accompanied by suppressed myocardial inflammation and NLRP3 signalling activation.

Subsequent investigations focused on p16's regulation of NLRP3 signalling revealed that p16 significantly enhances NLRP3 activation through STAT3 modulation, driving fibroblast senescence. Mechanistically, this regulation requires direct interaction between p16 and the STAT3 SH2 domain. Y705 phosphorylation, a canonical modification enabling STAT3 dimerisation, facilitates its polymerisation and enhances binding to target promoters.^45^ We demonstrate that p16 amplifies STAT3 Y705 phosphorylation. Recent work shows EZH2 promotes this phosphorylation through STAT3 di‐methylation, while stabilising STAT3 via K49 di‐methylation.^25^, ^26^ Our findings reveal that p16 enhances EZH2‐STAT3 interaction and elevates STAT3 K49 di‐methylation, with p16‐mediated STAT3 transcriptional activity on *NLRP3* being K49‐dependent. The SH2 domain of STAT3 is essential for both STAT3 polymerisation and phosphorylation at tyrosine residue 705 (Y705).^46^ We found p16 binds STAT3 via the SH2 domain to enhance Y705 phosphorylation. We propose that p16 may augment SH2 domain phosphorylation by modulating EZH2‐STAT3 interaction, thereby increasing STAT3 transcriptional activity and *NLRP3* expression. While the SH2 domain is essential for STAT3 polymerisation,^47^ whether p16‐enhanced SH2 phosphorylation affects polymerisation warrants further investigation.

STAT3 activates transcription by directly binding target promoters or indirectly disrupting histone‐modified protein interactions.^31^ To assess p16‐STAT3 complex effects on *NLRP3* promoter histone modifications, we analysed modification levels and found p16 decreased repressive H3K27me3 and H2AK119 ubiquitination while increasing active H3K4me3, indicating p16 modulates *NLRP3* promoter chromatin state to determine STAT3 transcriptional activity. As core catalytic components of the PRC family, the Bmi‐1‐EZH2 heterodimer, a well‐characterised histone‐modifying complex, orchestrates epigenetic gene silencing through site‐specific trimethylation of histone H3 at lysine 27.^48^, ^49^ While AhR^50^ and BCL6^51^ are established transcription factors regulating *NLRP3* promoter activity, our study reveals a novel mechanism where the p16‐STAT3 complex selectively disrupts BCL6 binding without affecting AhR occupancy. Specifically, p16 impedes the assembly of Bmi‐1‐containing complexes (Bmi‐1‐EZH2 and Bmi‐1‐BCL6) at the *NLRP3* promoter. Given that BCL6 normally forms a BCL6‐PRC1‐PRC2 repressive complex with Bmi‐1 and EZH2 to silence target genes,^52^ these findings extend the understanding of how p16‐STAT3 interactions reprogram the chromatin landscape to activate proinflammatory pathways in cardiac fibroblasts.

As a master transcription factor, STAT3 orchestrates senescence‐associated transcriptional reprogramming affecting apoptosis, metabolism and proliferation pathways.^53^ Our RNA‐seq analysis revealed that p16‐STAT3 complex formation significantly altered the expression of STAT3‐regulated metabolic genes (NAMPT, NNMT) during senescence. Notably, the NNMT/NAMPT imbalance may disrupt NADH/NADPH homeostasis, exacerbating oxidative stress and senescence.^54^, ^55^ These findings suggest p16 extends its regulatory repertoire beyond cell cycle control through STAT3‐dependent metabolic reprogramming. Accumulated p16 stably associates with STAT3, potentially reshaping genome‐wide STAT3 transcriptional output and chromatin landscapes. IL‐6, a key STAT3 upstream activator, critically drives inflammageing through undefined mechanisms despite its established role in promoting cellular senescence.^45^, ^56^, ^57^ Further epigenomic profiling of STAT3 targets under p16 influence is warranted to fully elucidate IL‐6's role in inflammageing.

Our findings demonstrated p16's detrimental role in post‐MI ventricular remodelling and inflammation via *NLRP3* transcriptional dysregulation, establishing p16 as a promising therapeutic target. However, clinical translation is hindered by the absence of specific p16 inhibitors. To address this, we engineered FH peptide‐modified nanoparticles for cardiac fibroblast‐specific delivery of *p16*‐siRNA. This nanotherapeutics effectively silenced p16 expression, attenuated myocardial fibrosis and restored cardiac function post‐MI without cytotoxic effects. Mechanistically, p16 knockdown suppressed NLRP3 inflammasome activation and SASP cytokine secretion (IL‐1β, IL‐6, TNF‐α), mitigating inflammageing in aged mice. Epigenetic profiling revealed that p16 silencing enhanced repressive H3K27me3/H2AK119ub marks at the *NLRP3* promoter by disrupting STAT3 binding and promoting Bmi‐1‐BCL6 complex formation. These data confirm the therapeutic utility of p16‐targeted nanomedicine in ageing‐related cardiovascular pathologies.

To enhance clinical relevance, we employed 12–14‐month‐old aged mice, given that most MI patients are elderly. This age selection aligns with reports demonstrating a significant increase in p16 protein levels after 12 months, attributed to senescent cell accumulation.^58^ While a prior study reported p16 upregulation in young mice post‐MI,^59^ our analysis of human samples revealed distinct age‐dependent expression patterns: p16 was low in young patients but significantly elevated in elderly MI cohorts. This disparity underscores the superiority of aged mice for modelling human MI pathophysiology. The conflicting results between the prior young mouse study and our aged model likely stem from inherent age‐related differences in basal p16 expression. Mechanistically, earlier work in neonatal mice established p16's canonical role in regulating cardiac fibroblast cell cycles, a classic mechanism of senescence induction. However, human and aged murine fibroblasts exhibit marked proliferative deficits compared to neonatal cells due to cumulative ageing effects. This limits the translational relevance of neonatal models. Our study addressed this critical gap by investigating aged mice and primary human/aged murine fibroblasts. We identified a novel p16‐driven mechanism in ageing, wherein p16 enhances *NLRP3* transcription via modulating STAT3 K49 di‐methylation and Bmi‐1‐BCL6 complex formation, thereby exacerbating inflammageing and post‐MI ventricular remodelling. This work not only overcomes prior limitations but also provides clinically actionable insights into age‐related MI progression.

In summary, in ageing cardiac fibroblasts, p16 accumulation enhances EZH2‐mediated STAT3 K49 di‐methylation, which disrupts Bmi‐1‐BCL6 complex assembly at the *NLRP3* promoter. This epigenetic reprogramming upregulates STAT3‐dependent *NLRP3* transcription, driving inflammageing‐associated ventricular remodelling post‐MI. These findings identify dual therapeutic strategies: (1) inhibiting p16 accumulation or (2) disrupting p16‐STAT3 interaction using small molecules. Notably, the novel nanomaterial FNLM‐*p16*‐siRNA shows promise as a targeted therapeutic candidate for MI management in aged populations.

## AUTHOR CONTRIBUTIONS

Conceptualisation, Xin Gu, Jianliang Jin and Fang Wang; Methodology, Xin Gu, Yingqiang Du, Jin'ge Zhang, Jiyu Li, Haiyun Chen, Yujie Lin, Yue Wang, Chunli Zhang, Shiyu Lin, Nannan Hao, Chengyi Peng, Jiacheng Ge, Jin Liu, Yan Liang, Yongjie Zhang, Xiaoyan Wang, Fang Wang and Jianliang Jin; Software, Xin Gu, Yingqiang Du, Jin'ge Zhang, Jiyu Li, Yongjie Zhang, Fang Wang and Jianliang Jin; Validation, Xin Gu, Yingqiang Du, Jin'ge Zhang, Jiyu Li, Haiyun Chen, Yujie Lin, Yue Wang, Chunli Zhang, Shiyu Lin, Nannan Hao, Chengyi Peng, Xiaoyan Wang, Jiacheng Ge and Jianliang Jin; Collection of Human Heart Tissues, Xin Gu, Yingqiang Du, Yongjie Zhang, Xiaoyan Wang, Fang Wang and Jianliang Jin; Data Analysis, Xin Gu, Yingqiang Du, Fang Wang and Jianliang Jin; Writing – Original Draft, Xin Gu and Yingqiang Du, with help from the other authors; Writing – Review & Editing, Jianliang Jin and Fang Wang, with help from the other authors; Project Administration and Supervision, Xin Gu, Jianliang Jin and Fang Wang; Funding Acquisition, Jianliang Jin, Fang Wang, Xiaoyan Wang and Yingqiang Du.

## CONFLICT OF INTEREST STATEMENT

The authors declare no conflicts of interests.

## ETHICS STATEMENT

Please find them in Materials and Methods section.

## Supporting information



SI1: Figures S1–S10

SI2: Figures S1–S10 Legends

SI3: Graphical Abstract

SI4: Graphical Abstract Legend

SI5: Complete Materials and Methods

SI6: Basic information of coronary artery disease and MI patients

SI7: Basic information of MI patients of different ages

SI8: Differentially expressed genes from the cardiac fibroblasts between p16‐CKO and control mice following MI

SI9: Tables S1‐S3

SI10: Original blots

## Data Availability

All datasets and methodological materials underlying this study are made publicly accessible to enable research reproducibility and methodological extension. The RNA‐seq datasets generated from this study are publicly archived under BioProject accession code PRJNA1100178. The dataset is titled ‘The mRNA sequencing of primary fibroblasts from the infarcted area in hearts of *p16^f/f^POSTN‐iCre* and *p16^f/f^* mice following myocardial infarction’. Full data access is provided through the NCBI BioProject repository via the following URL: https://www.ncbi.nlm.nih.gov/bioproject/PRJNA1100178/.
