# Supplementary material for: Fibroblasts‐specific p16INK4a exacerbates inflammageing‐mediated post‐infarction ventricular remodelling through interacting with STAT3 to regulate NLRP3 transcription
Source: Clin Transl Med. 2025 Jun 3;15(6):e70344. doi: 10.1002/ctm2.70344 (PMC12134396; doi:10.1002/ctm2.70344)
Supplement: Supplementary file 1 — SI1: Figures S1–S10 [file CTM2-15-e70344-s009.pdf]

Figure S1

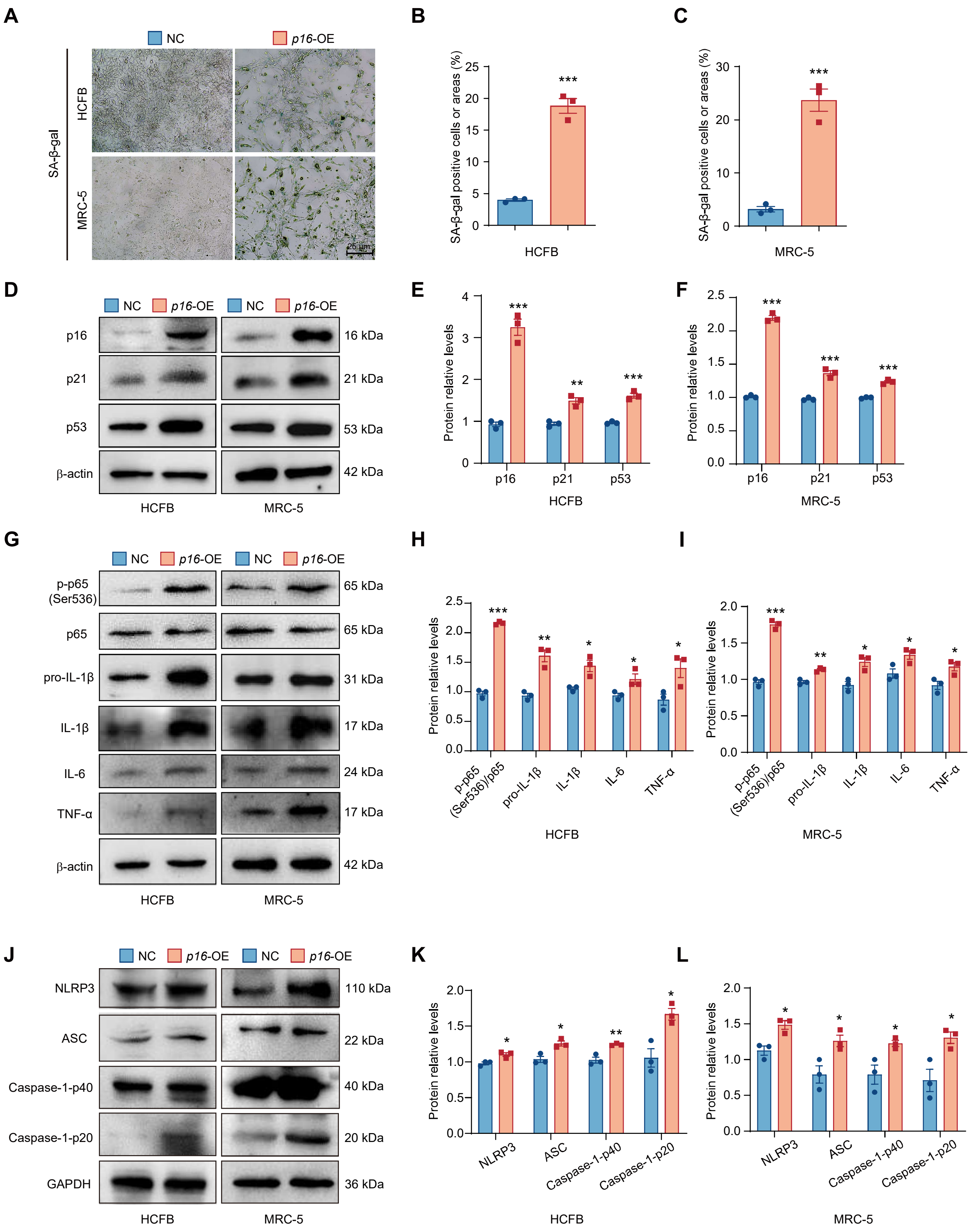

A
NC
p16-OE
SA-β-gal
HCFB
MRC-5
B
SA-β-gal positive cells or areas (%)
***
HCFB
C
***
MRC-5
D
p16
p21
p53
β-actin
16 kDa
21 kDa
53 kDa
42 kDa
HCFB
MRC-5
E
Protein relative levels
F
G
p-p65 (Ser536)
p65
pro-IL-1β
IL-1β
IL-6
TNF-α
65 kDa
31 kDa
17 kDa
24 kDa
H
p-p65 (Ser536)/p65
I
J
NLRP3
ASC
Caspase-1-p40
Caspase-1-p20
GAPDH
110 kDa
22 kDa
40 kDa
20 kDa
36 kDa
K
L

Figure S2

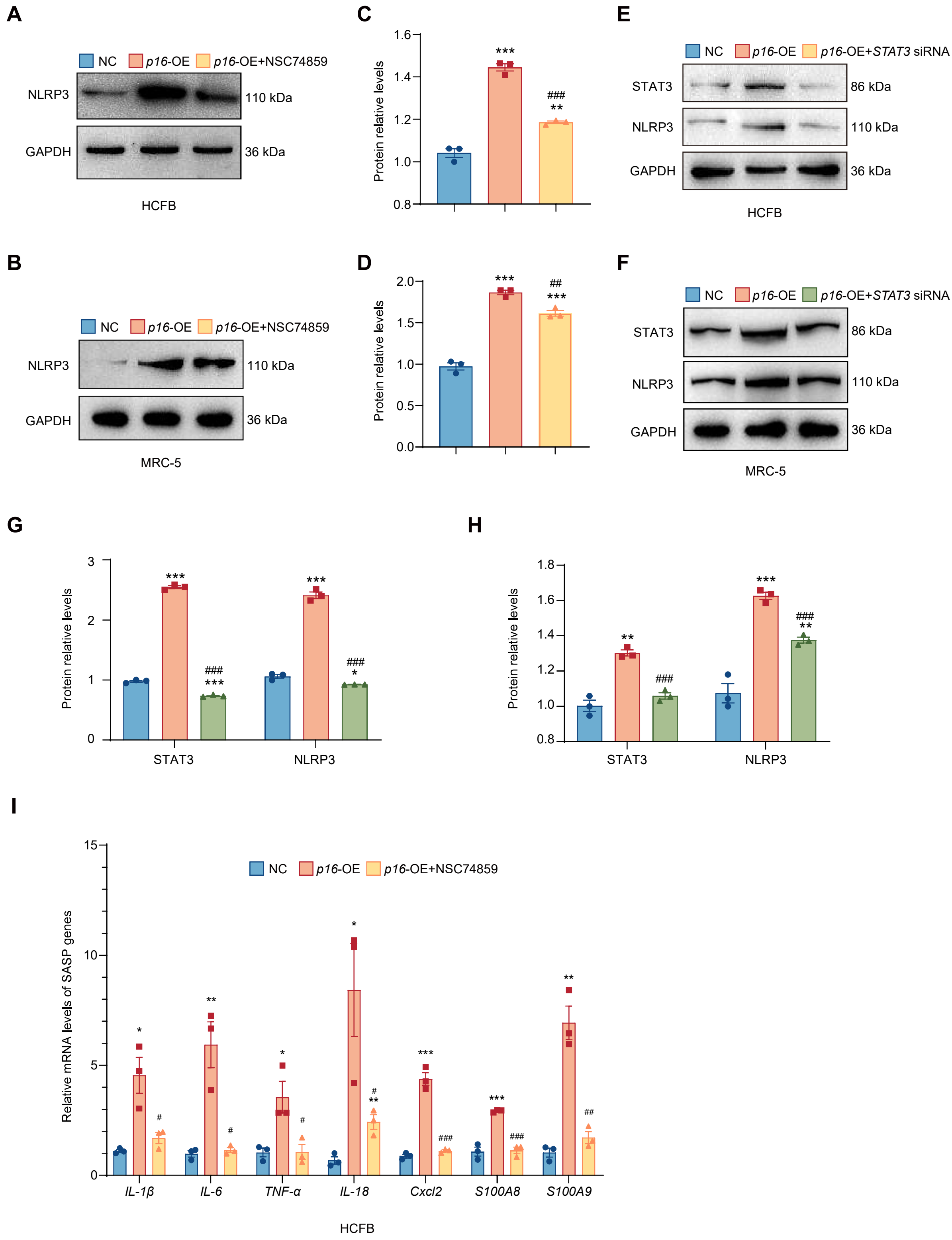
A
NC
p16-OE
p16-OE+NSC74859
NLRP3
110 kDa
GAPDH
36 kDa
HCFB
B
NC
p16-OE
p16-OE+NSC74859
NLRP3
110 kDa
GAPDH
36 kDa
MRC-5
C
Protein relative levels
D
Protein relative levels
E
NC
p16-OE
p16-OE+STAT3 siRNA
STAT3
86 kDa
NLRP3
110 kDa
GAPDH
36 kDa
HCFB
F
NC
p16-OE
p16-OE+STAT3 siRNA
STAT3
86 kDa
NLRP3
110 kDa
GAPDH
36 kDa
MRC-5
G
Protein relative levels
STAT3
NLRP3
H
Protein relative levels
STAT3
NLRP3
I
NC
p16-OE
p16-OE+NSC74859
Relative mRNA levels of SASP genes
IL-1β
IL-6
TNF-α
IL-18
Cxcl2
S100A8
S100A9
HCFB

Figure S3

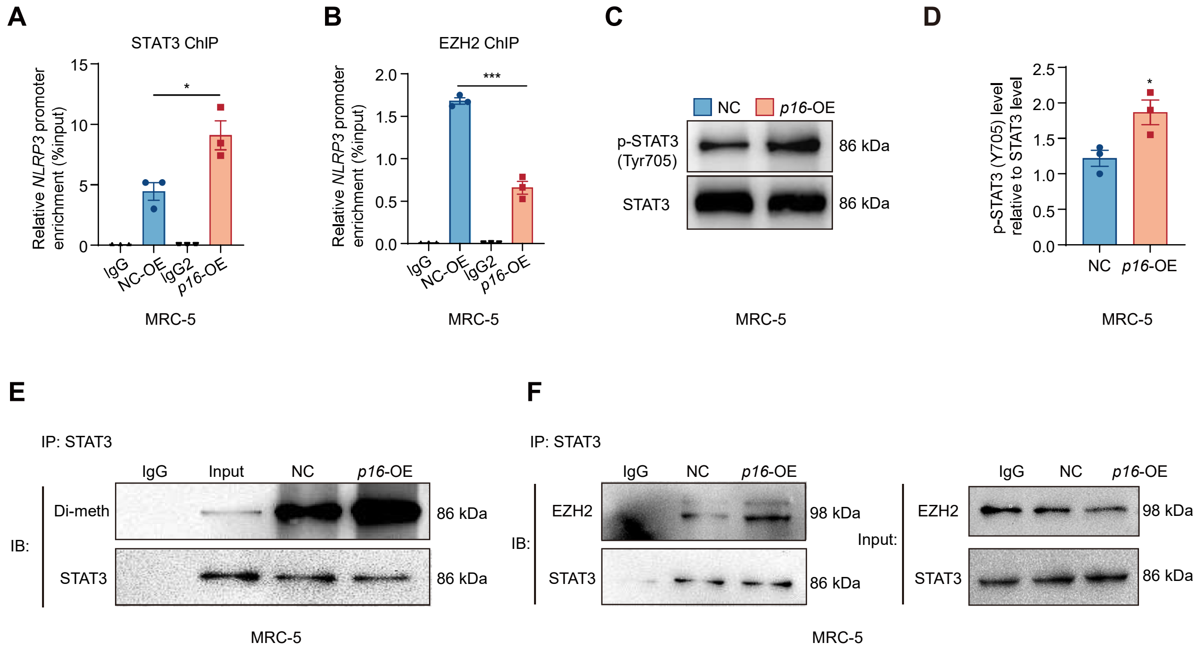

A
STAT3 ChIP
Relative NLRP3 promoter enrichment (%input)
*
IgG
NC-OE
IgG2
p16-OE
MRC-5
B
EZH2 ChIP
Relative NLRP3 promoter enrichment (%input)
***
IgG
NC-OE
IgG2
p16-OE
MRC-5
C
NC
p16-OE
p-STAT3 (Tyr705)
86 kDa
STAT3
86 kDa
MRC-5
D
p-STAT3 (Y705) level relative to STAT3 level
*
NC
p16-OE
MRC-5
E
IP: STAT3
IgG
Input
NC
p16-OE
IB:
Di-meth
86 kDa
STAT3
86 kDa
MRC-5
F
IP: STAT3
IgG
NC
p16-OE
IB:
EZH2
98 kDa
STAT3
86 kDa
Input:
IgG
NC
p16-OE
EZH2
98 kDa
STAT3
86 kDa
MRC-5

Figure S4

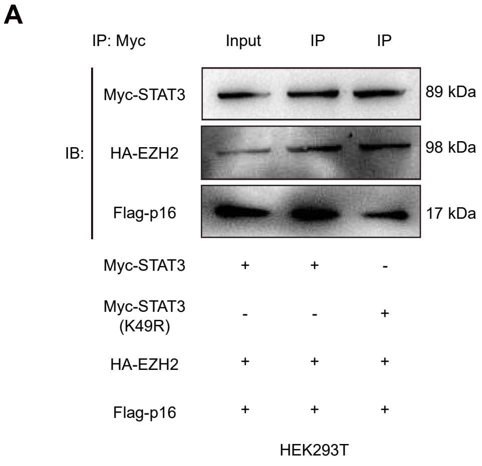
A
IP: Myc
Input
IP
IP
IB:
Myc-STAT3
HA-EZH2
Flag-p16
89 kDa
98 kDa
17 kDa
Myc-STAT3 + + -
Myc-STAT3 (K49R) - - +
HA-EZH2 + + +
Flag-p16 + + +
HEK293T

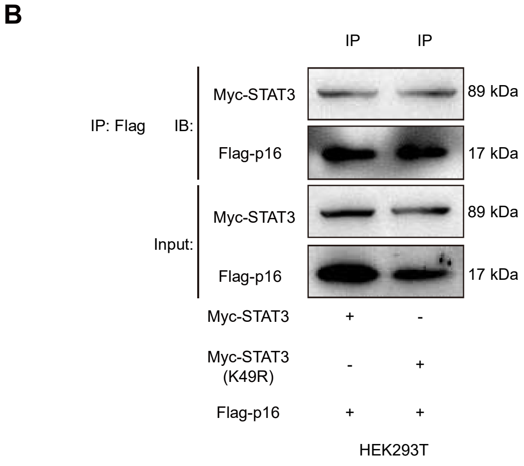
B
IP
IP
IP: Flag
IB:
Myc-STAT3
Flag-p16
89 kDa
17 kDa
Input:
Myc-STAT3
Flag-p16
89 kDa
17 kDa
Myc-STAT3 + -
Myc-STAT3 (K49R) - +
Flag-p16 + +
HEK293T

Figure S5-1

A

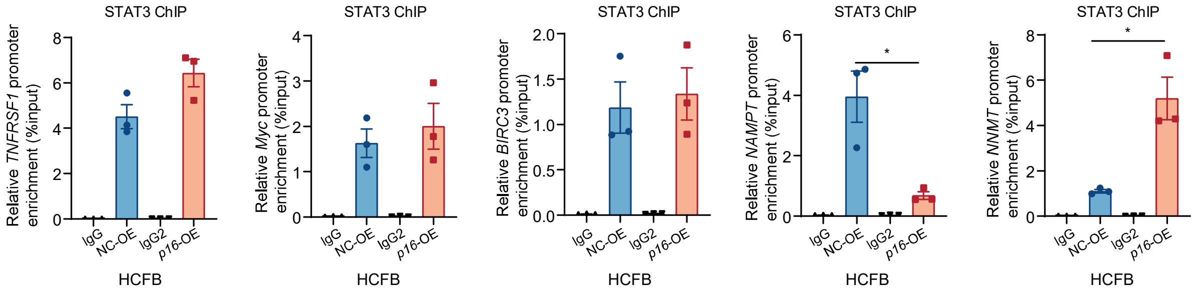

B

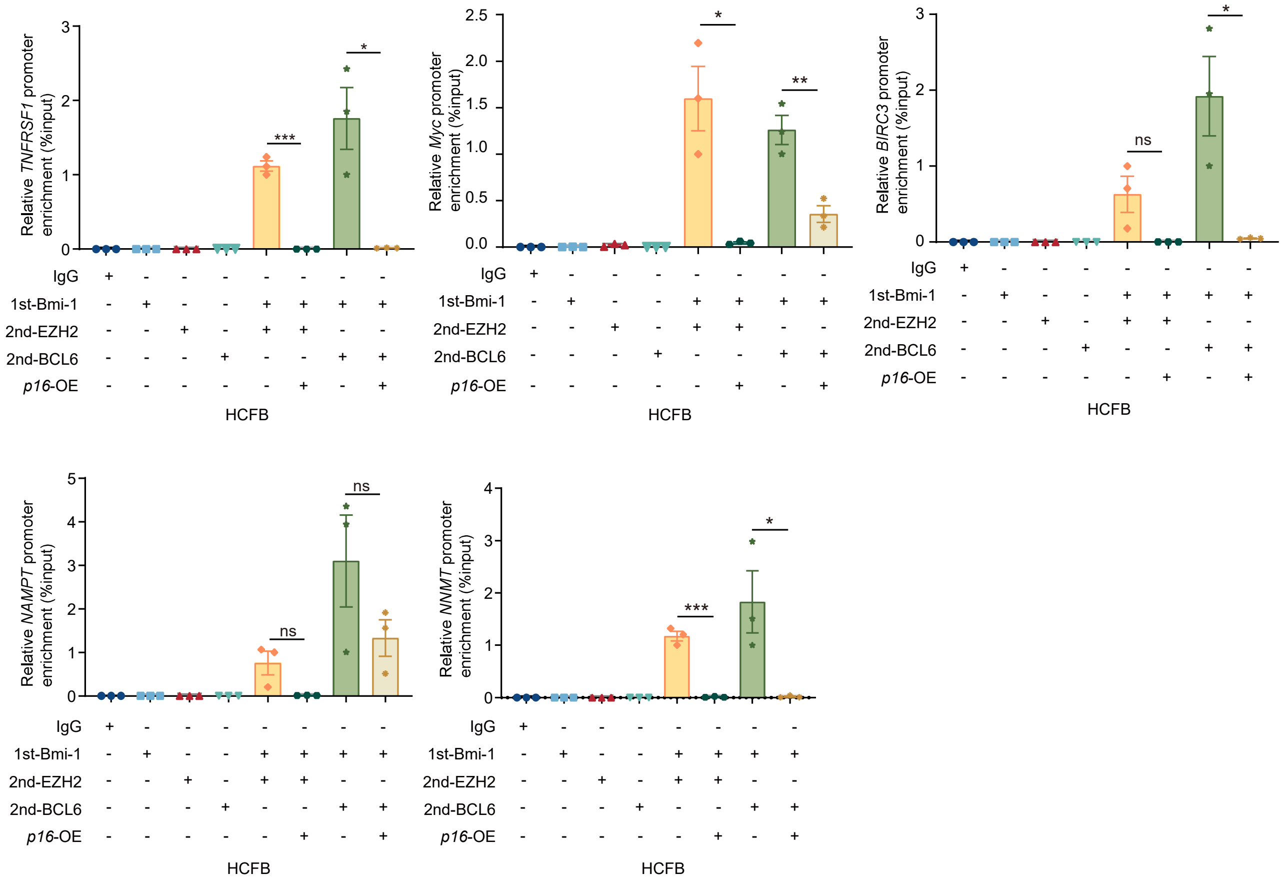

Figure S5-2

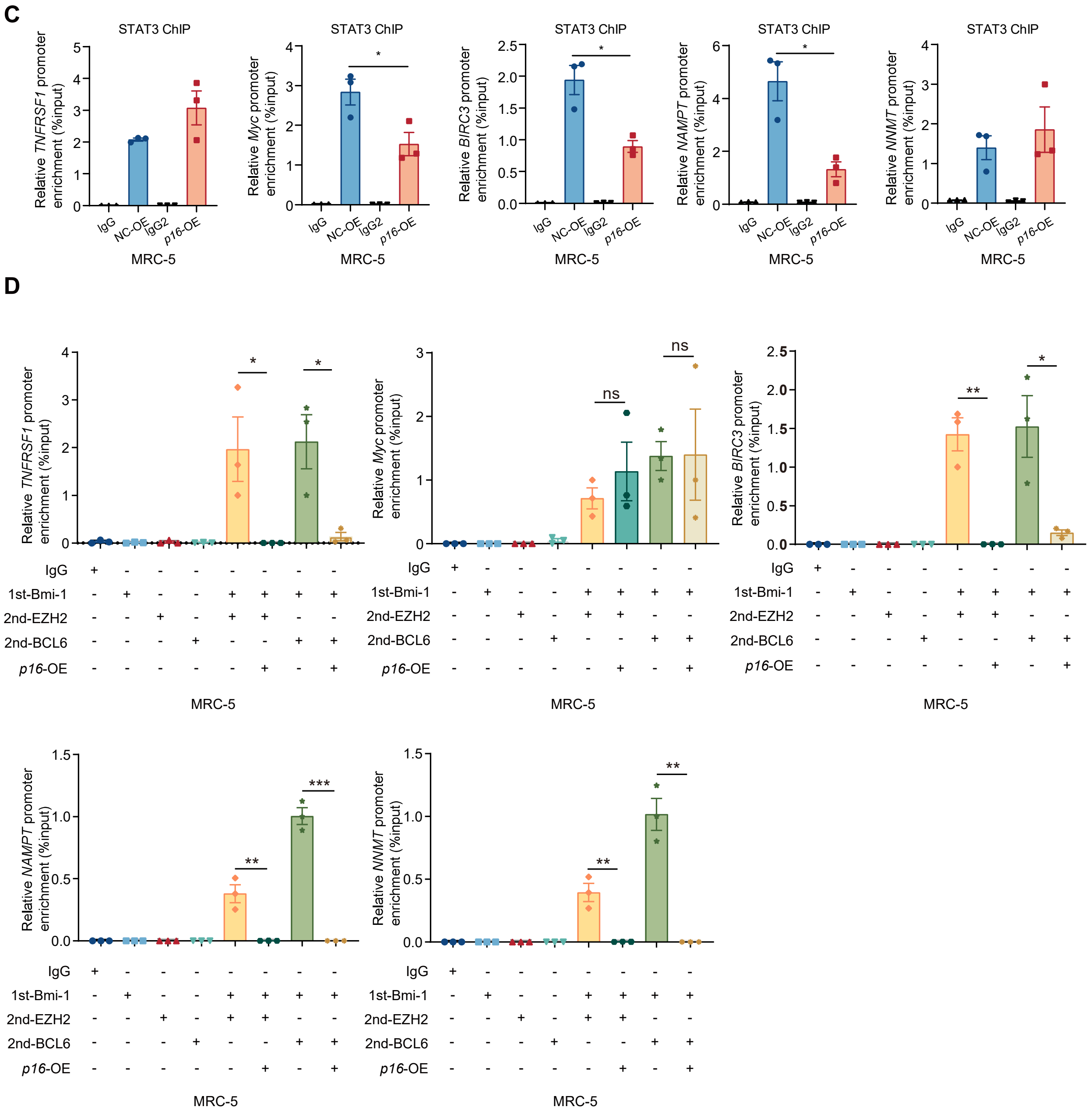

C
STAT3 ChIP
Relative TNFRSF1 promoter enrichment (%input)
Relative Myc promoter enrichment (%input)
Relative BIRC3 promoter enrichment (%input)
Relative NAMPT promoter enrichment (%input)
Relative NNMT promoter enrichment (%input)
IgG
NC-OE
IgG2
p16-OE
MRC-5
D
Relative TNFRSF1 promoter enrichment (%input)
Relative Myc promoter enrichment (%input)
Relative BIRC3 promoter enrichment (%input)
Relative NAMPT promoter enrichment (%input)
Relative NNMT promoter enrichment (%input)
ns
IgG
1st-Bmi-1
2nd-EZH2
2nd-BCL6
p16-OE
MRC-5

Figure S6

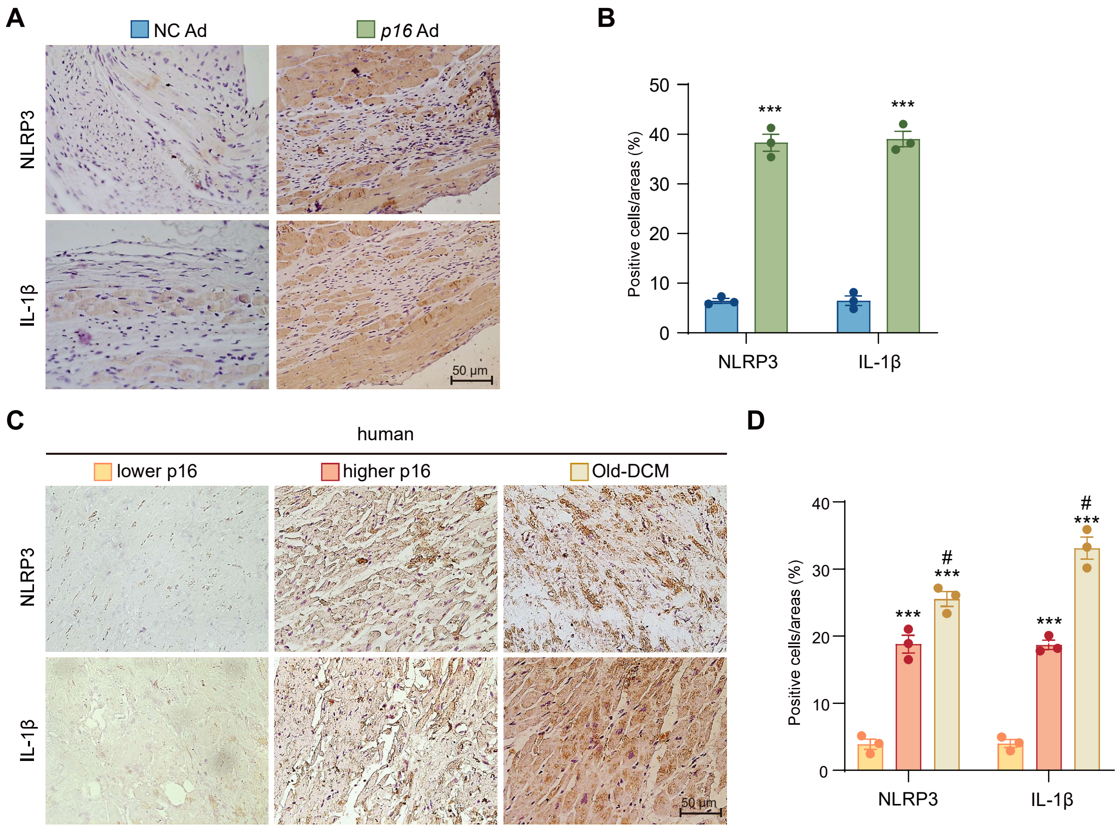

A
NC Ad
p16 Ad
NLRP3
IL-1β
50 μm
B
Positive cells/areas (%)
50
40
30
20
10
0
***
***
NLRP3
IL-1β
C
human
lower p16
higher p16
Old-DCM
NLRP3
IL-1β
50 μm
D
Positive cells/areas (%)
40
30
20
10
0
***
***
#
***
***
#
***
NLRP3
IL-1β

Figure S7

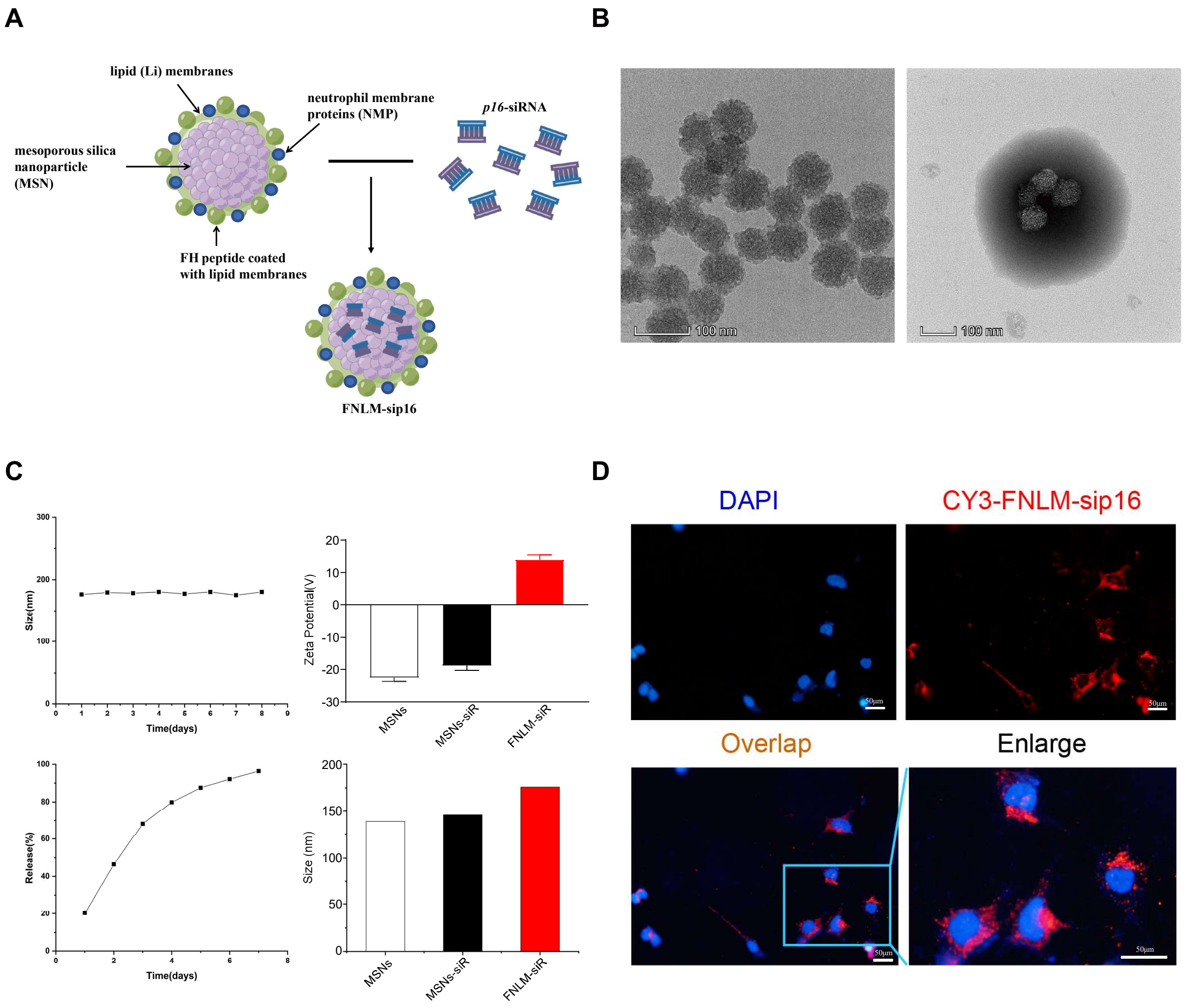
A
lipid (Li) membranes
neutrophil membrane proteins (NMP)
p16-siRNA
mesoporous silica nanoparticle (MSN)
FH peptide coated with lipid membranes
FNLM-sip16
B
100 nm
100 nm
C
Size(nm)
Time(days)
Zeta Potential(V)
MSNs
MSNs-siR
FNLM-siR
Release(%)
Time(days)
Size (nm)
MSNs
MSNs-siR
FNLM-siR
D
DAPI
CY3-FNLM-sip16
Overlap
Enlarge
50μm

Figure S8

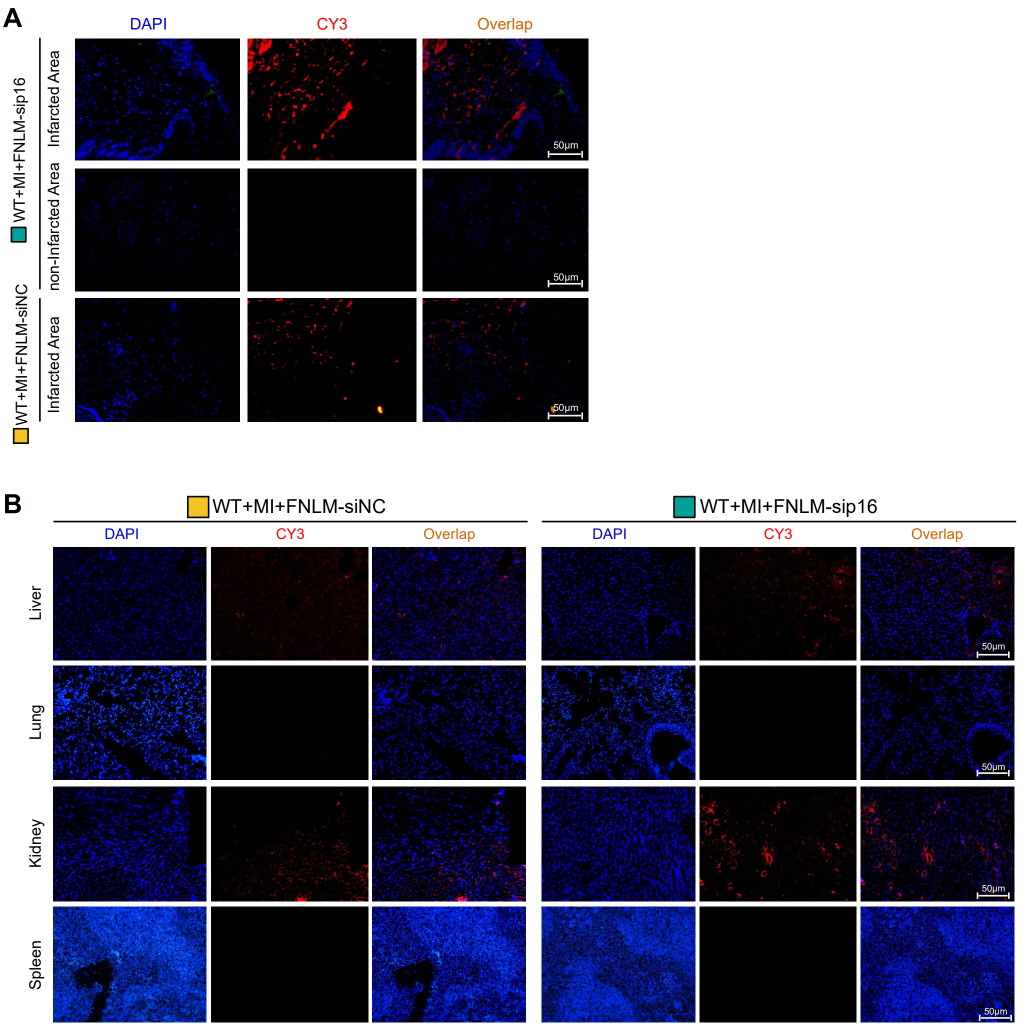
A
DAPI
CY3
Overlap
WT+MI+FNLM-sip16
Infarcted Area
non-Infarcted Area
WT+MI+FNLM-siNC
Infarcted Area
50μm
50μm
50μm
B
WT+MI+FNLM-siNC
WT+MI+FNLM-sip16
DAPI
CY3
Overlap
DAPI
CY3
Overlap
Liver
Lung
Kidney
Spleen
50μm
50μm
50μm
50μm

Figure S9

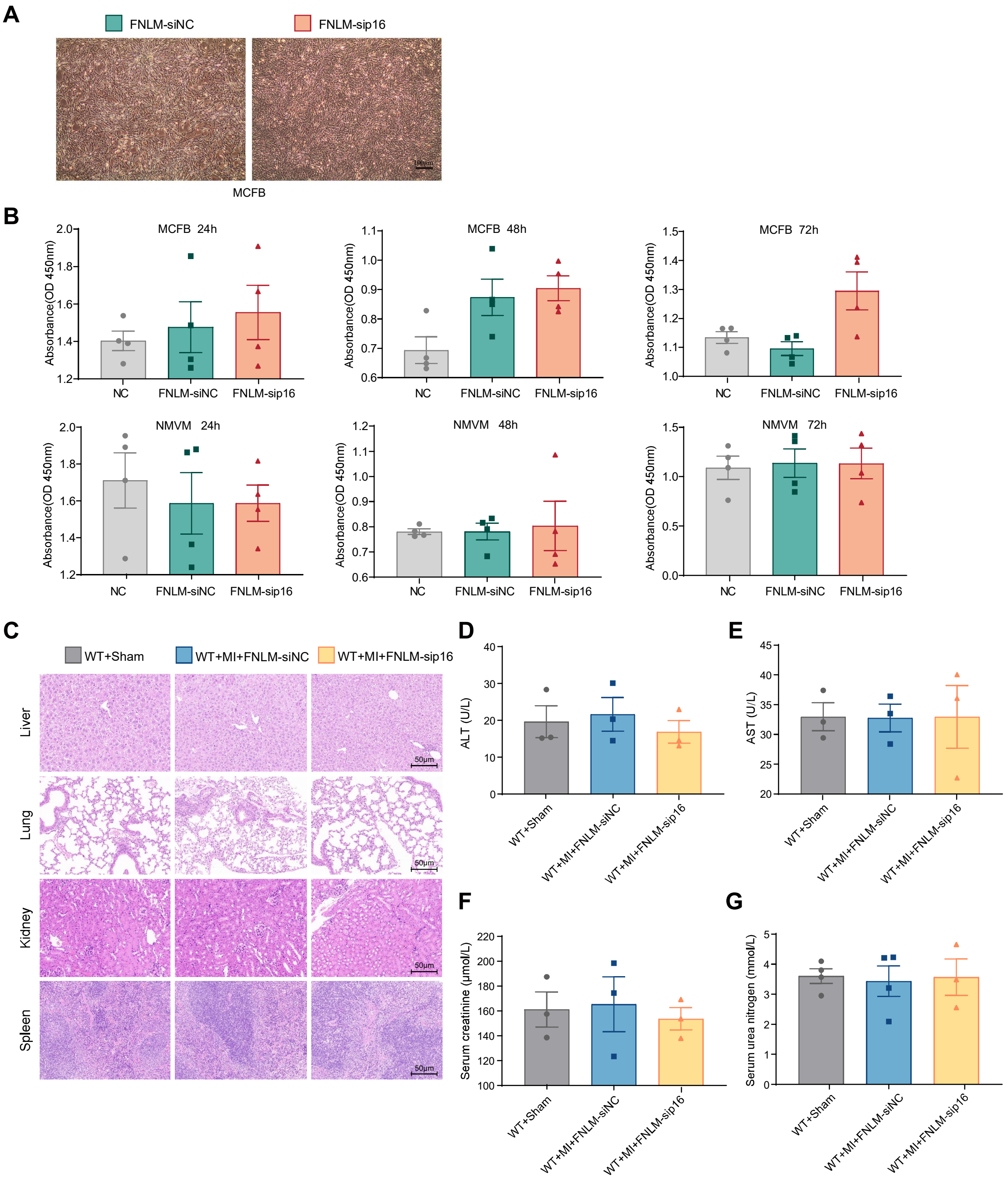

A
FNLM-siNC
FNLM-sip16
MCFB
B
MCFB 24h
MCFB 48h
MCFB 72h
NMVM 24h
NMVM 48h
NMVM 72h
Absorbance(OD 450nm)
NC
FNLM-siNC
FNLM-sip16
C
WT+Sham
WT+MI+FNLM-siNC
WT+MI+FNLM-sip16
Liver
Lung
Kidney
Spleen
50μm
D
ALT (U/L)
E
AST (U/L)
F
Serum creatinine (μmol/L)
G
Serum urea nitrogen (mmol/L)

Figure S10

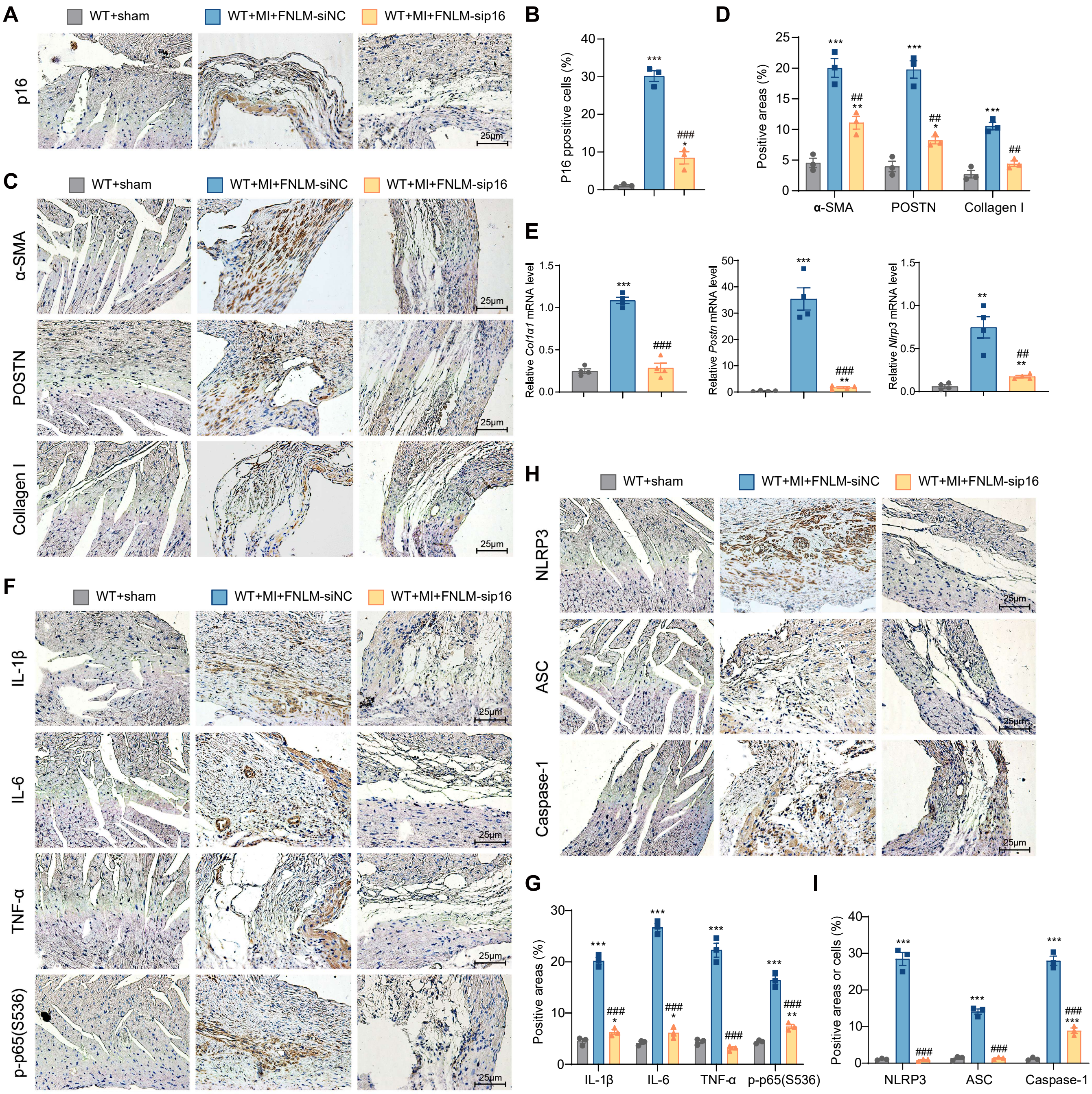
A
WT+sham
WT+MI+FNLM-siNC
WT+MI+FNLM-sip16
p16
25μm
B
P16 ppositive cells (%)
C
α-SMA
POSTN
Collagen I
D
Positive areas (%)
E
Relative Col1a1 mRNA level
Relative Postn mRNA level
Relative Nlrp3 mRNA level
F
IL-1β
IL-6
TNF-α
p-p65(S536)
G
H
NLRP3
ASC
Caspase-1
I
Positive areas or cells (%)
