# Supplementary material for: Fibroblasts‐specific p16INK4a exacerbates inflammageing‐mediated post‐infarction ventricular remodelling through interacting with STAT3 to regulate NLRP3 transcription
Source: Clin Transl Med. 2025 Jun 3;15(6):e70344. doi: 10.1002/ctm2.70344 (PMC12134396; doi:10.1002/ctm2.70344)
Supplement: Supplementary file 2 — SI2: Figures S1–S10 Legends [file CTM2-15-e70344-s008.docx]

**SI1:** **Figures S1-S10 and Legends**

**Figure S1 *P16* overexpression induces inflammaging and NLRP3 signaling activation in fibroblasts.**

Human cardiac fibroblasts (HCFB cells) and human fetal lung fibroblast (MRC-5 cells) were transduced with adenovirus vectors encoding either *p16* overexpression (*p16*-OE) or negative control (NC) constructs. (A) Representative micrographs of HCFB and MRC-5 cells stained for senescence associated-β-galactosidase (SA-β-gal). (B-C) Percentage of HCFB or MRC-5 cells' positive area for SA-β-gal. (D) Western blots of cells extracts showing p16, p21 and p53, β-actin was the loading control. (E-F) Protein levels relative to NC group were assessed by densitometric analysis. (G) Western blots of cells extract showing p-p65(Ser536), p65, pro-IL-1β, IL-1β, IL-6 and TNF-α, β-actin was the loading control. (H-I) Protein levels relative to NC group were assessed by densitometric analysis. (J) Western blots of cells extract showing NLRP3, ASC and Caspase-1-p40/p20, GAPDH was the loading control. (K-L) Protein levels relative to NC group were assessed by densitometric analysis. Three biological replicates were used per experiment. ^*^P<0.05, ^**^P<0.01, ^***^P<0.001 compared to NC group, unpaired Student’s *t*-test.

**Figure S2 *P16* overexpression upregulates NLRP3 expression mediated by STAT3 in fibroblasts.**

After transfection with *p16* overexpression (*p16*-OE) adenovirus and NC, HCFB and MRC-5 cells were treated with a transcriptional inhibitor of STAT3, NSC74859, and examined for NLRP3 expression. (A-B) NLRP3 Western blots of HCFB and MRC-5 cell extract after treatment with NSC74859; GAPDH was the loading control. (C-D) Protein levels were assessed by densitometric analysis. (E-F) Western blots of HCFB and MRC-5 cell extracts, showing STAT3 and NLRP3 after treatment with STAT3 siRNA; GAPDH was the loading control. (G-H) Protein levels were assessed by densitometric analysis. (I) RT-qPCR of HCFB cell extract showing *IL-1β*, *IL-6*, *TNF-α*, *IL-18*, *Cxcl2*, *S100A8* and *S100A9*, and *Gapdh* was the loading control. Values are means ± SEM of six determinations. ^*^P<0.05, ^**^P<0.01, ^***^P<0.001 compared with the NC group; ^#^P<0.05, ^##^P<0.01, ^###^P<0.001 compared with the *p16*-OE group, one-way ANOVA test.

**Figure S3 P16 promotes the interaction between EZH2 and STAT3, and increases the K49 di-methylation and Y705 phosphorylation of STAT3 by EZH2 in MRC-5 cells.**

MRC-5 cells were transfected with *p16* overexpression (*p16*-OE) adenovirus. (A-B) The binding levels of STAT3 or EZH2 in the *NLRP3* promoter region in MRC-5 cells were detected by ChIP. (C) Western blots of cell extracts showing p-STAT3(Tyr705); STAT3 was the loading control. (D) Protein levels relative to those in the NC group were assessed by densitometric analysis. Three biological replicates were used per experiment. ^*^P<0.05 compared with the NC group, unpaired Student’s *t*-test. (E) MRC-5 cell proteins were extracted for anti-STAT3 immunoprecipitation. Western blots were used to detect di-methylation levels and STAT3. (F) MRC-5 cell proteins were extracted for anti-STAT3 immunoprecipitation. Western blots were used to detect EZH2 and STAT3.

**Figure S4 Mutation of K49 of STAT3 did not interfere the interaction between STAT3 and EZH2 or p16.**

(A) HEK293T cells were co-transfected with *Myc-STAT3* plasmid and/or *Myc-STAT3*(*K49R*) plasmid, *HA-EZH2* plasmid and *Flag-p16* overexpression adenovirus. Cell’s proteins were extracted for anti-Myc immunoprecipitation. Western blots were used to detect Myc, HA and Flag. (B) HEK293T cells were co-transfected with *Myc-STAT3* plasmid and/or *Myc-STAT3*(*K49R*) plasmid and *Flag-p16* overexpression adenovirus. Cell’s proteins were extracted for anti-Flag immunoprecipitation. Western blots were used to detect Myc and Flag.

**Figure S5 P16 affects the senescence associated key downstream target genes regulated by STAT3.**

HCFB cells were transfected with *p16* overexpression (*p16*-OE) adenovirus. (A) The binding levels of STAT3 in the promoter regions of *TNFRSF1*, *Myc*, *BIRC3*, *NAMPT* and *NNMT* in HCFB cells were detected by ChIP assay. Three biological replicates were used per experiment. ^*^P<0.05 compared to NC group, unpaired Student’s *t*-test. (B) The binding levels of Bmi-1-EZH2 complex or Bmi-1-BCL6 complex in the promoter regions of *TNFRSF1*, *Myc*, *BIRC3*, *NAMPT* and *NNMT* in HCFB cells were detected by Re-ChIP assay. Three biological replicates were used per experiment. ^*^P<0.05, ^**^P<0.01, ^***^P<0.001, one-way ANOVA test. (C) MRC-5 cells were transfected with *p16* overexpression (*p16*-OE) adenovirus. The binding levels of STAT3 in the promoter regions of *TNFRSF1*, *Myc*, *BIRC3*, *NAMPT* and *NNMT* in MRC-5 cells were detected by ChIP assay. Three biological replicates were used per experiment. ^*^P<0.05 compared to NC group, unpaired Student’s *t*-test. (D) The binding levels of Bmi-1-EZH2 complex or Bmi-1-BCL6 complex in the promoter regions of *TNFRSF1*, *Myc*, *BIRC3*, *NAMPT* and *NNMT* in MRC-5 cells were detected by Re-ChIP assay. Three biological replicates were used per experiment. ^*^P<0.05, ^**^P<0.01, ^***^P<0.001, one-way ANOVA test.

**Figure S6 P16 aggravates NLRP3 signaling activation.**

Eight-week-old MI mice were anesthetized via intraperitoneal injection of pentobarbital sodium (50 mg/kg) prior to receiving either *p16* overexpression adenovirus (*p16* Ad) or negative control adenovirus (NC Ad) delivered to both infarcted and peri-infarct regions. Four weeks later, the levels of NLRP3 and IL-1β were detected with immunohistochemistry. (A) Representative micrographs of hearts for NLRP3 and IL-1β. (B) The percentage of cells or areas for NLRP3 or IL-1β positive relative to total cells or areas. Three biological replicates were used per experiment. ^***^P<0.001 compared with the NC adenovirus treatment group, unpaired Student’s *t*-test. (C) Human myocardial tissues from healthy and patients with DCM were examined for p16 protein levels by ELISA. Representative micrographs of hearts from people with low p16 expression, people with high p16 expression and patients with DCM, stained for NLRP3 and IL-1β with immunohistochemistry. (D) The percentage of cells or areas positive for NLRP3 or IL-1β relative to total cells or areas. Three biological replicates were used per experiment. ^***^P<0.001 compared with the lower-p16-expressed group; ^#^P<0.05 compared with higher-p16-expressing group, one-way ANOVA test.

**Figure S7 Construction of nanoparticles targeting cardiac fibroblasts.**

(A) Construction mode diagram of FNLM-*p16*-siRNA. (B) Representative transmission electron scope images of FNLM-siRNA. (C) Stability of FNLM-siRNA over time in PBS; Zeta potential of MSNs, MSNs-siRNA and FNLM-siRNA; Release profile of siRNA from FNLM-siRNA in PBS with 10% FBS over time; Diameter of MSNs, MSNs-siRNA and FNLM-siRNA. (D) Representative images of fluorescence labeled FNLM-siRNA after incubation for 30 min with cardiac fibroblasts.

**Figure S8 *In vivo* targeting profile of nanoparticles in MI mice.**

Following MI, 12-month-old mice received weekly tail vein injections of FNLM-*p16*-siRNA for 4 consecutive weeks. At 13 months of age, animals were humanely euthanized via intraperitoneal administration of pentobarbital sodium (100 mg/kg), and hearts along with major organs (liver, spleen, lung, kidney) were harvested and fixed in 4% paraformaldehyde. (A) Immunofluorescent images of heart sections showing FNLM-*p16*-siRNA (FNLM-sip16) or FNLM-NC-siRNA (FNLM-siNC) targeted accumulation in infarcted area and non-infarcted area. (B) Immunofluorescent images of sections showing FNLM-siNC or FNLM-sip16 targeted accumulation in liver, lung, kidney and spleen.

**Figure S9 *In vivo* safety of nanoparticles.**

(A) Image of cell growth of mouse cardiac fibroblasts (MCFB) at 24 h after treatment with FNLM-NC-siRNA (FNLM-siNC) or FNLM-*p16*-siRNA (FNLM-sip16). (B) *In vitro* toxicity measurement of FNLM-siNC or FNLM-sip16 treating MCFB and neonatal murine ventricular myocytes (NMVMs) for different time was analyzed using Cell Counting Kit-8 assay. Twelve-month-old WT mice were anesthetized via intraperitoneal injection of pentobarbital sodium (50 mg/kg) and subjected to MI model induction. Following this, nanoparticles carrying either FNLM-*p16*-siRNA (FNLM-sip16) or FNLM-NC-siRNA (FNLM-siNC) were administered via caudal vein injection once weekly for 4 consecutive weeks. (C) H&E staining was applied to histological sections of major organs including liver, lung, kidney, and spleen. Levels of ALT (U/L) (D), AST (U/L) (E), creatinine (μmol/L) (F) and urea nitrogen (mmol/L) (G) in serum. Three biological replicates were used per experiment, one-way ANOVA test.

**Figure S10 FNLM-*p16*-siRNA ameliorates myocardial fibrosis and proinflammatory response after MI.**

Twelve-month-old WT mice were anesthetized via intraperitoneal injection of pentobarbital sodium (50 mg/kg) and subjected to MI model induction. Following this, nanoparticles carrying either FNLM-*p16*-siRNA (FNLM-sip16) or FNLM-NC-siRNA (FNLM-siNC) were administered via caudal vein injection once weekly for 4 consecutive weeks. (A-B) Representative image and statistical figure of paraffin-embedded heart ventricular wall sections of mice stained for p16. (C) Representative micrographs of paraffin-embedded heart ventricular wall sections of mice stained for α-SMA, Collagen I and POSTN. (D) The percentage of areas positive for α-SMA, Collagen I and POSTN relative to total areas. (E) The mRNA levels of *Col1α1*, *Postn*, and *Nlrp3* in hearts from FNLM-siNC-treated mice and FNLM-sip16-treated mice. (F) Representative micrographs of paraffin-embedded heart ventricular wall sections of mice stained for IL-1β, IL-6, TNF-α and p-p65(S536). (G) Statistical figure of paraffin-embedded heart ventricular wall sections of mice stained for IL-1β, IL-6, TNF-α and p-p65(S536). (H) Representative micrographs of paraffin-embedded heart ventricular wall sections of mice stained for NLRP3, ASC and Caspase-1. (I) Statistical figure of paraffin-embedded heart ventricular wall sections of mice stained for NLRP3, ASC and Caspase-1. Three or four biological replicates were used per experiment. ^*^P<0.05, ^**^P<0.01, ^***^P<0.001 compared with WT + sham group. ^##^P<0.01, ^###^P<0.001 compared with WT+MI+ FNLM-siNC group, one-way ANOVA test.
