# Supplementary figures and images for: Fibroblasts‐specific p16INK4a exacerbates inflammageing‐mediated post‐infarction ventricular remodelling through interacting with STAT3 to regulate NLRP3 transcription
Source: Clin Transl Med. 2025 Jun 3;15(6):e70344. doi: 10.1002/ctm2.70344 (PMC12134396; doi:10.1002/ctm2.70344)

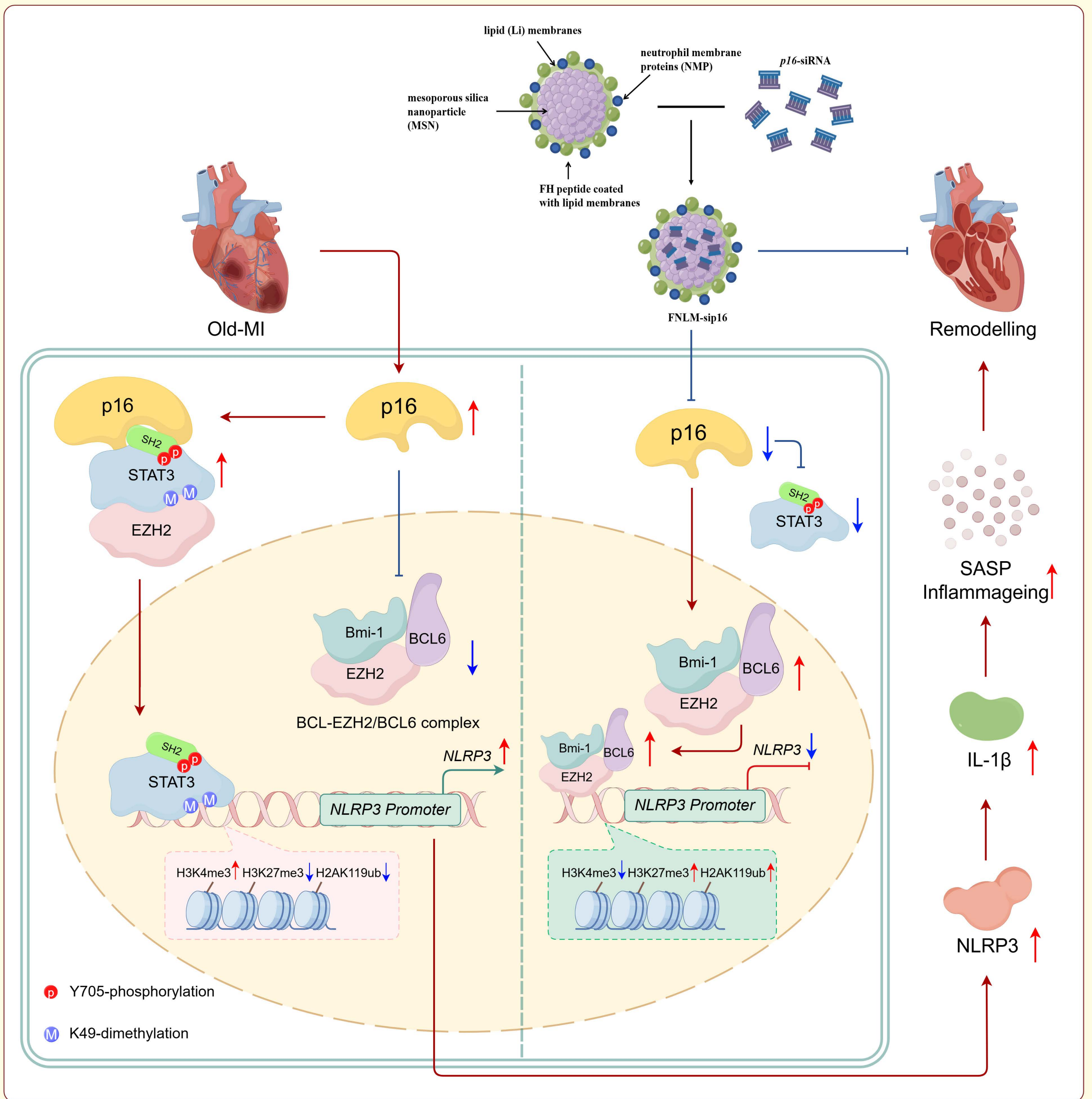

Supplement: Supplementary file 3 — SI3: Graphical Abstract [file CTM2-15-e70344-s010.pdf]

**Figure S11**

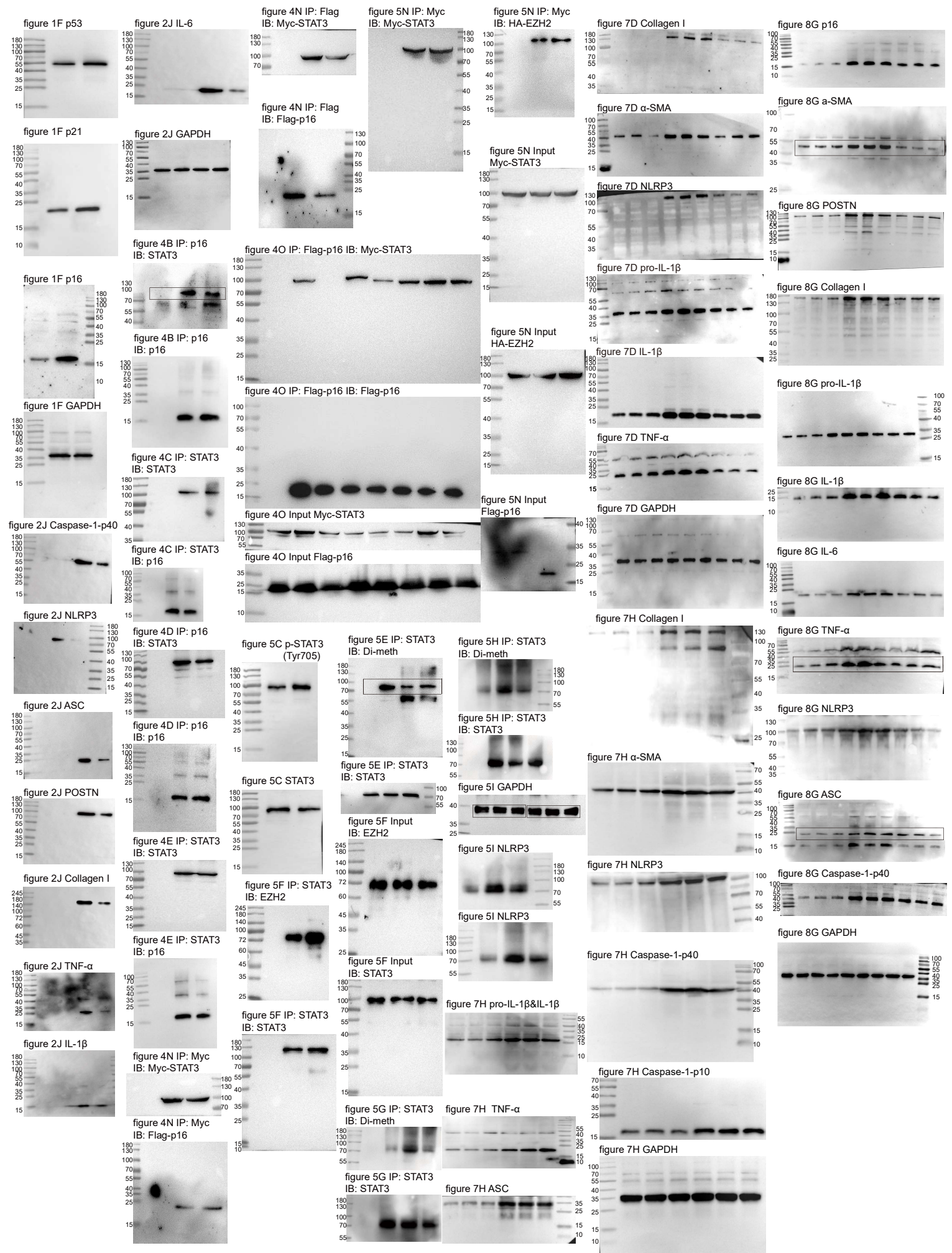

### Figure S12

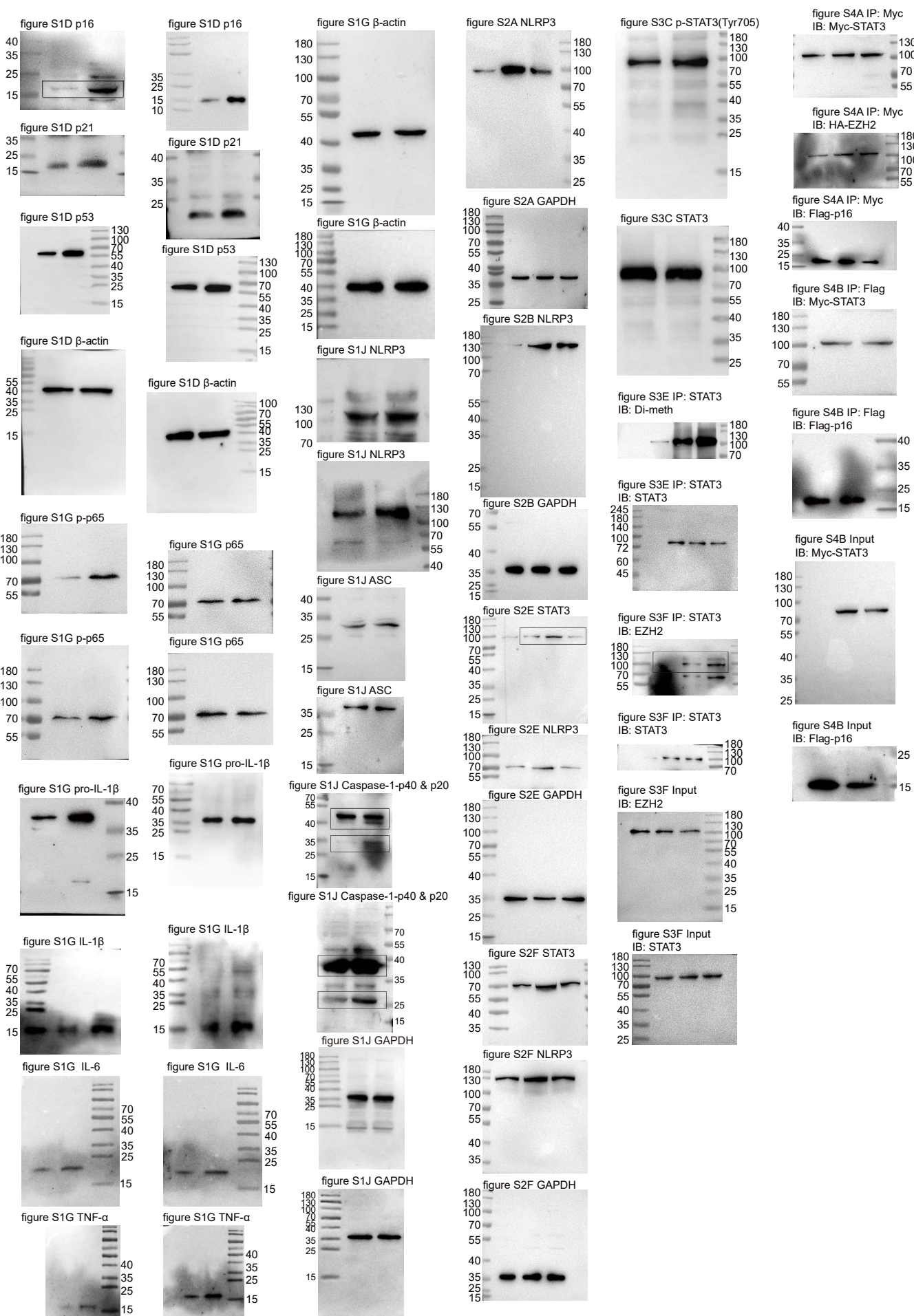

Supplement: Supplementary file 10 — SI10: Original blots [file CTM2-15-e70344-s005.pdf]
