# Supplementary material for: Fibroblasts‐specific p16INK4a exacerbates inflammageing‐mediated post‐infarction ventricular remodelling through interacting with STAT3 to regulate NLRP3 transcription
Source: Clin Transl Med. 2025 Jun 3;15(6):e70344. doi: 10.1002/ctm2.70344 (PMC12134396; doi:10.1002/ctm2.70344)
Supplement: Supplementary file 4 — SI4: Graphical Abstract Legend [file CTM2-15-e70344-s002.docx]

**SI4: Graphical Abstract Legend**

1. In ageing fibroblasts, p16^INK4a^ concurrently enhanced STAT3 di-methylation mediated by EZH2 and suppressed the assembly of Bmi-1-EZH2/BCL-6 complexes at the *NLRP3* promoter region.
2. These dual regulatory actions synergistically upregulated STAT3-dependent *NLRP3* transcription, thereby exacerbating inflammageing-driven ventricular remodelling following myocardial infarction.
3. FNLM-nanocaged *p16^INK4a^*-siRNA prevents post-infarction ventricular remodelling through inhibiting *NLRP3* transcription in targeted cardiac fibroblasts.
