# Supplementary material for: Fibroblasts‐specific p16INK4a exacerbates inflammageing‐mediated post‐infarction ventricular remodelling through interacting with STAT3 to regulate NLRP3 transcription
Source: Clin Transl Med. 2025 Jun 3;15(6):e70344. doi: 10.1002/ctm2.70344 (PMC12134396; doi:10.1002/ctm2.70344)
Supplement: Supplementary file 5 — SI5: Complete Materials and Methods [file CTM2-15-e70344-s003.docx]

**SI5: Complete Materials and Methods**

**Mice and genotyping**

C57BL/6Smoc-*Postn^em(2A-Cre)1Smoc^* inducible Cre (hereafter *POSTN-iCre*) mice (No. NM-KI-225034) with C57BL/6 background were obtained from Shanghai Model Organisms (China). *POSTN-iCre* genotyping employed primers 5’- GGAGCAATGGTCACTTTTGACA-3’ (forward) and 5’- AGGTTCTGCGGGAAACCATT-3’ (reverse).

The *p16* (*Cdkn2a*) gene was modified using CRISPR/Cas9 technology. Briefly, the CRISPR/Cas9 system and donor template were delivered via microinjection into fertilized C57BL/6J mouse oocytes. Successfully edited F0 founders were screened via PCR and Sanger sequencing, followed by breeding with wild-type C57BL/6JGpt counterparts to establish a stable F1 generation knockout model. The *p16* conditional knockout (*p16^f/f^*) murine model with C57BL/6J genetic background was generated by GemPharmatech Co., Ltd in Nanjing of China. *Loxp1* allele was detected via forward primer 5’-CTCAGGGATGACCTGTGTTATCC-3’ and reverse primer 5’-TGGACTACCAGAATACGCTGGAG-3’; *Loxp2* allele was detected using primer below: Forward: 5’-CAGCTCTTGCGTAAGCAGATTTG-3’ and Reverse: 5’-CACAACGGGTTCTTCTGTTAGTCC-3’. The *p16^f/f^POSTN-iCre* mice were generated through sequential mating of *p16^f/f^* mice with *POSTN-iCre* transgenic mice.

One week prior to acute myocardial infarction (MI) induction, the *p16^f/f^POSTN-iCre* mice received tamoxifen (50 mg/kg, T5648, Sigma, USA) dissolved in corn oil via daily intraperitoneal injection (i.p.) for five consecutive days to trigger genetic recombination.

Twelve-month-old *p16* homozygous (*p16*-KO) male mice and their wild-type (WT) male littermates were generated and genotyped as our described previously ^1, 2^[.](#_ENREF_2)

All animal procedures strictly complied with NIH guidelines (Publication No. 8, revised 2011) and were approved by Institutional Animal Care and Use Committee of Nanjing Medical University (IACUC-1706001).

Prior to MI model establishment, surgical anesthesia was induced in mice via intraperitoneal delivery of pentobarbital sodium (50 mg/kg body weigh). For cardiac function assessment, anesthesia was maintained using isoflurane (4% induction, 1.5% maintenance) delivered in oxygen, and Color Doppler echocardiography was performed. A humane endpoint was achieved through intraperitoneal treatment of pentobarbital sodium (100 mg/kg).

**MI model construction**

MI was surgically induced under pentobarbital sodium anesthesia (50 mg/kg, i.p.) through permanent ligation of left anterior descending (LAD) coronary artery, positioned 2-3 mm distal to the auricular appendage as established in standard protocols [^3^](#_ENREF_3). Following endotracheal intubation, artificial mechanical ventilation was maintained using a volume-controlled mode with defined parameters: inspiratory-to-expiratory ratio of 2:1, tidal volume set at 2 ml, and respiratory rate regulated at 125 breaths per minute. Surgical thoracotomy allowed visualization of the heart, and the LAD was ligated using an 8-0 nylon suture. The successful establishment of MI was verified by the presence of electrocardiographic ST-segment elevation in conjunction with echocardiographic evidence of blanching in the left ventricular anterior wall. Sham-operated controls underwent identical surgical procedures, including thoracotomy and ventilation, but without LAD ligation.

**Color Doppler echocardiography**

At 4 weeks post-MI, anesthesia was induced and maintained in mice through inhalation of isoflurane, administered in a 1:1 oxygen-to-air mixture, with induction at concentrations of 4% (v/v) and maintenance with 1.5% (v/v). Cardiac function was assessed by transthoracic echocardiography via Vevo® 2100 high-resolution ultrasound system (VisualSonics, Inc., Toronto, Canada) with 30-MHz phased-array transducer. Left ventricular ejection fraction (LVEF) and fractional shortening (LVFS) were measured from M-mode images using standardized methodologies as previously described ^3, 4^[.](#_ENREF_4)

**Patient enrollment, blood procurement, and peripheral blood mononuclear cells (PBMCs) isolation**

Isolation for blood sampling was approved by the Ethics Committee of The Affiliated Hospital of Jiangnan University (Approval No. K2022031K01), ensuring all procedures strictly adhered to ethical principles outlined in the Declaration of Helsinki. All participants had signed informed consent. PBMCs were isolated from collected blood samples following manufacturer-provided protocols. MI patients were stratified into two cohorts in accordance with p16 expression levels detected in PBMCs, with coronary artery disease patients serving as controls. Following standardized pharmacological intervention, participants underwent one-year follow-up evaluations encompassing cardiac function assessments, hematological analyses, and magnetic resonance imaging examinations. Detailed baseline demographics of coronary artery disease and MI cohorts are presented in **Supplementary Information 6**.

Additionally, we recruited patients at 6-12 months post-MI and age-matched healthy controls. Participants were stratified into two age cohorts: 20-25 years (young group) and 55-65 years (older group, including older MI patients). Following collection of peripheral blood samples, serum and PBMCs were isolated according to standardized protocols. All prior enrolled participant had signed informed consent. Detailed baseline characteristics of age-stratified MI patients are presented in **Supplementary Information 7**.

**Human myocardial tissue samples**

Human myocardial tissue samples were obtained from 25 autopsy donors through the Department of Human Anatomy of Nanjing Medical University. The study was performed in strict adherence to the ethical guidelines established by the 1975 Declaration of Helsinki. Both anatomical procedures and experimental protocols received approval from the Nanjing Medical University Ethics Committee (Approval No. 2019-902). Donors were enrolled, aged 39-94 years, without documented history of neoplasms, congenital heart defects, valvular abnormalities, autoimmune disorders, chronic infections, inflammatory conditions, tuberculosis, syphilis, acquired immunodeficiency syndrome, or other significant comorbidities prior to demise.

**Solid-phase microarray of proteome profiling on inflammatory factors of human plasma**

Plasma samples of healthy people and patients from each group were collected to detect each expression level of thirty-six pro-inflammatory factors using a solid-phase microarray platform (Shanghai Universal Biotech Co., Ltd, China) with Proteome Profiler Human Cytokine Array Kit (ARY005B, R&D Systems Inc., MN, USA) following manufacturer's protocols, as outlined in prior publications [^5^](#_ENREF_5). Briefly, the assay is based on immobilizing capture antibodies onto a membrane or glass slide, followed by sample incubation. Subsequently, a biotin-labeled detection antibody specific to the target protein is introduced. Signal detection is achieved through sequential incubation with either horseradish peroxidase (HRP)-conjugated streptavidin or fluorescein-conjugated streptavidin. For enhanced visualization, chemiluminescent substrates or HiLyte™ Fluor 555-labeled streptavidin are employed as the final detection reagents.

**Bioinformatics Analysis**

High-throughput RNA sequencing data (GSE115031) were obtained from the Gene Expression Omnibus (GEO) database (available at: <https://www.ncbi.nlm.nih.gov/geo/>), comprising three biological replicates each of control and infarct organoids. Following analysis in R (v4.0.3), pairwise gene correlations were computed using Pearson's method to generate correlation coefficients for subsequent gene set enrichment analysis (GSEA) as previously described [^6^](#_ENREF_6).

**Protein-Protein Docking Analysis**
Protein-protein docking simulations were conducted following established protocols [^7^](#_ENREF_7). The atomic coordinates of p16 and STAT3 were downloaded from the Protein Data Bank (PDB). Subsequent protein structure of protein preparation was performed utilizing Discovery Studio 2019 Client software (Dassault Systèmes BIOVIA, San Diego, CA, USA) through standardized preprocessing including hydration shell removal and hydrogen atom supplementation. Molecular docking was performed via the HDOCK server, which implements a global search algorithm utilizing fast Fourier transform for conformational sampling of potential protein-protein interfaces. Candidate complexes were refined through iterative knowledge-based scoring functions. Binding affinity and dissociation constants of the protein-peptide interactions were visualized using PyMOL software.

**High-throughput sequencing analysis**

Twelve-month-old *p16^f/f^POSTN-iCre* mice and *p16^f/f^* littermates were anesthetized via intraperitoneal injection of pentobarbital sodium (50 mg/kg), followed by induction of acute MI. One week prior to MI induction, the *p16^f/f^POSTN-iCre* mice received tamoxifen (50 mg/kg) dissolved in corn oil via daily intraperitoneal injection for five consecutive days to trigger genetic recombination. After a 4-week observation period of MI, the *p16^f/f^POSTN-iCre* and *p16^f/f^* mice were humanely sacrificed through i.p. of pentobarbital sodium at a dosage of 100 mg/kg. Primary cardiac fibroblasts isolated from infarcted myocardial regions underwent mRNA amplification-based transcriptome sequencing. DEGs were identified using the “FindAllMarkers” algorithm (|log₂FC| > 1, *p* < 0.05) in R (v4.0.3), followed by bioinformatics analyses, including Kyoto Encyclopedia of Genes and Genomes (KEGG) pathway mapping, Gene Ontology (GO) enrichment, GSEA, and transcriptional factor binding site network construction, as previously described ^4, 8^[. Sequencing and analytical procedures were performed by APPLIED PROTEIN TECHNOLOGY Co., Ltd. (Shanghai, China). Complete DEG datasets are provided in](#_ENREF_8)**Supplemental Information 8.**

**Mouse cardiac fibroblasts culture**

Mice were humanely sacrificed through intraperitoneal administration of pentobarbital sodium at a dosage of 100 mg/kg, and hearts were aseptically harvested using blunt dissection followed by three washes in antibiotic-supplemented phosphate-buffered saline (PBS; 0.01 mM phosphate, pH 7.4). As previously described [^9^](#_ENREF_9), hearts were excised, minced into 1-mm³ fragments, and subjected to collagenase type II/trypsin mixture (0.4 mg/ml Collagenase type II; 0.25% trypsin) (Gibco, USA) under agitation (9 cycles × 7 min, 250 rpm, 37℃). Cycle supernatants were neutralized with prewarmed horse serum, and pooled cell suspensions were centrifuged (600 ×g, 10 s) before resuspension and plating on poly-L-lysine-coated culture ware. Following 1-hour incubation (37°C, 5% CO₂), non-adherent cardiomyocytes were eliminated via media exchange. The fibroblast cultures were propagated in DMEM supplemented with 10% FBS and penicillin/streptomycin (all from Gibco) under standard culture conditions (37℃, 5% CO₂).

**Neonatal murine ventricular myocytes (NMVMs)** **culture**

Neonatal mice (P1-3) were anesthetized by hypothermia before cardiac excision and ventricular tissue isolation. Ventricular tissues were mechanically dissociated in 2 ml collagenase D (1mg/ml) solution (Roche Diagnostics GmbH, Mannheim, Germany), 2% FBS (v/v), and DMEM/F12(Gibco) as previously described [^4^](#_ENREF_4). Digestion proceeded at 37℃ (30 min) with constant agitation (175 rpm). Post-enzymatic dissociation, supernatants were discarded, and cell pellets were washed thrice in PBS prior to resuspension in complete medium (10% FBS, penicillin/streptomycin in DMEM-F12). The cells were plated in 6-well culture dishes and incubated in a humidified atmosphere (5% CO₂, 37°C) for 90 min. Suspended cardiomyocytes were transferred to fresh wells, and spontaneous contractions were confirmed. Primary cardiomyocytes were harvested after 4-6 days in culture, with first-passage NMVMs utilized for experimental assays.

**Cell lines**

Human cardiac fibroblasts (HCFB; #ZQ6300) and human fetal lung fibroblasts (MRC-5; #ZQ0006) were procured from Shanghai Zhong Qiao Xin Zhou Biotechnology Co., Ltd. (Shanghai, China), while HEK293T cell line was sourced from Genechem Co., Ltd. (Shanghai, China). All cell lines were propagated according to the manufacturers’ recommended protocols.

**Nanoparticle preparation**

Human peripheral blood neutrophils were sourced from Zenbio (Chengdu Zhengneng Biotechnology Co., Ltd., China), and their plasma membranes were isolated following previously described methods ^10, 11^[. Mesoporous silicon nanoparticles (MSNs) were synthesized by dissolving hexadecyl trimethyl ammonium bromide (CTAB) and triethanolamine in distilled water, incubating at 60](#_ENREF_11)℃ for 1 hour, adding cyclohexane and tetraethyl orthosilicate (TEOS), and stirring for 24 hours. The products were collected via centrifugation, redispersed in ethanol, and purified by stirring with ammonium nitrate to eliminate residual CTAB. The final MSNs were dispersed in distilled water at 20 mg/ml. *P16*-siRNA was complexed with MSNs in the presence of calcium chloride under ultrasonication for 15 minutes. For lipid coating preparation, 1,2-dimyristoyl-sn-glycero-3-phosphocholine (DMPC), 1,2-distearoyl-sn-glycero-3-phosphoethanolamine-N- [methoxy (polyethylene glycol)-2000] (DSPE-PEG-NHS), and 1,2-dioleoyl-3-trimethylammonium-propane (DOTAP) (all from Avanti Polar Lipids, USA) were dissolved in chloroform at a molar ratio of 76.2:3.8:20, and lipid films were formed by solvent evaporation. FH peptide (sequence: FHKHKSPALSPV; China Peptide Co., Ltd.) was conjugated to DSPE-PEG-NHS at a 2:1 molar ratio and stirred overnight at room temperature to form DSPE-PEG-FH. This was mixed with neutrophil membrane protein, briefly sonicated, and incubated at room temperature for 6 hours to embed the membrane protein. The lipid-coated MSNs-*p16* siRNA were hydrated with an aqueous suspension, sonicated for 30 minutes, and sequentially extruded through polycarbonate membranes (1000, 400, 200 nm). FH-NMP-LiMSNs-*p16* siRNA (FNLM-sip16) was obtained by centrifugation to remove excess components and resuspended in PBS.

**Nanoparticle characterization**

To evaluate the stability of FNLM-siRNA nanoparticles, samples were incubated in DMEM containing 10% FBS at ambient temperature (25℃) over defined intervals. Hydrodynamic diameters were measured at sequential time points using dynamic light scattering (DLS).

For siRNA release assessment, nanoparticles were resuspended in PBS with 10% bovine serum albumin (BSA) and maintained at physiological temperature (37℃). Supernatants were collected via centrifugation at predetermined intervals to quantify siRNA liberation kinetics.

***In vivo* or *in vitro* targeting profile of FNLM-siRNA nanoparticles**

***In vivo Biodistribution Analysis***
To evaluate the in vivo targeting efficiency of FNLM-*p16*-siRNA nanoparticles, twelve-month-old male C57BL/6 mice were selected as experimental subjects and received weekly tail vein injections (100 μl) for 4 consecutive weeks post-MI. At 13 months of age, animals were euthanized via i.p. of pentobarbital sodium (100 mg/kg). Hearts and major organs (liver, spleen, lung, kidney) were harvested, fixed in 4% paraformaldehyde (PFA), and processed for ex vivo imaging using a ZEISS LSM confocal microscopy system.

***In vitro Endosomal Escape Assessment***
For intracellular trafficking analysis, primary cardiac fibroblasts were cultured on glass-bottom confocal dishes and treated with FNLM-siRNA nanoparticles for 24 hours. Following PBS washes, cells were counterstained with DAPI for nuclear visualization. Confocal microscopy was employed to monitor nanoparticle internalization and endosomal escape dynamics.

***Supplementary Data***
Sequences of *p16*-targeting siRNA, negative control siRNA, and corresponding mRNA targets are detailed in **Supplemental Information 9** **Table S1**.

**Safety assessment of FNLM-siRNA nanoparticles**

***In vitro cytotoxicity evaluation***

Cellular viability was quantified with the Cell Counting Kit-8 (CCK-8) (Biosharp, China) according to standard protocols. Cardiac fibroblasts and primary cardiomyocytes derived from C57BL/6 mice (2×10^3^ cells/well in 96-well plates) were exposed to FNLM-siRNA nanoparticles during 24-, 48-, or 72-hours incubation. Following CCK-8 reagent addition (10 μl/well), samples underwent 2h incubation (37℃) prior to 450 nm absorbance measurement using a microplate reader.

***In vivo biosafety analysis***For in vivo safety validation, 12-month-old C57BL/6 mice were subjected to myocardial infarction (MI) induction and intravenously injected with 200 μl PBS, FNLM, or FNLM-siRNA every two days for one week. Blood samples were collected on days 1 and 28 post-injection to assess serum biomarkers: alanine aminotransferase (ALT) and aspartate aminotransferase (AST) and creatinine (Cr) and urea nitrogen (BUN) (Nanjing Jiancheng Bioengineering Institute, China). Following euthanasia, major organs were collected from the mice, fixed in 4% paraformaldehyde solution, following paraffin embedding, sectioning, and hematoxylin and eosin (H&E) staining.

**Histology**

Euthanasia was performed via intraperitoneal administration of pentobarbital sodium (100 mg/kg). Both murine and human cardiac tissues were fixed in periodate-lysine-paraformaldehyde solution and placed at 4℃ [^2^](#_ENREF_2). Murine hearts were paraffin-embedded, coronally sectioned at 5 μm via Leica RM2255 rotary microtome, and processed through xylene deparaffinization followed by rehydration through a descending ethanol gradient series (100%, 95%, 70%) down to distilled water (dH₂O) prior to staining procedures. Human or mouse frozen cardiac tissues were embedded in Tissue-Tek® O.C.T. Compound and sectioned at 8 μm using Thermo Scientific Cryotome FSE cryostat [^2-4^](#_ENREF_2).

**Masson’s trichrome staining**

Paraffin sections were deparaffinized, rehydrated, and stained using a Masson's trichrome kit (#KGMST-8003, KeyGen Biotech, China) according to manufacturer's protocol [^2^](#_ENREF_2).

**Immunohistochemical staining**

Paraffin sections underwent deparaffinization, rehydration, and microwave-based antigen retrieval in 10 mM sodium citrate buffer (pH 6.0) for 20 min. After blocking, tissue sections were incubated with primary antibodies at cold room (4℃) for 8h followed by established staining protocols as previously detailed ^2, 4^[.](#_ENREF_4) Primary antibodies were against collagen I (#1310-08, Southern Biotech, AL, USA), α-SMA (ab28052), IL-1β (ab9722) from Abcam (MA, USA). IL-6 (A0286, ABclonal Biotechnology Co., Ltd., MA, USA), TNF-α (GTX110520, GeneTex Inc., CA, USA), RANTES (GTX31207, GeneTex Inc., USA), NLRP3 (ab263899, Abcam, USA) and ASC (ab155970, Abcam, USA).

**Immunofluorescent staining**

Cardiac tissues from distinct experimental groups were processed by embedding in Tissue-Tek® O.C.T. Compound (#4583, Sakura Finetek) and sectioning at 8-μm thickness. Fibroblasts cultured on glass coverslips were fixed with PLP post-treatment. Primary antibodies against p16 (ab108349, Abcam, USA) (reactivity: Human), p16 (ab211542, Abcam, USA) (reactivity: Mouse), STAT3 (#9139, Cell Signaling Technology, MA, USA), EZH2 (#5246, Cell Signaling Technology, USA), NLRP3 (ab263899, Abcam, USA) and IL-1β (ab9722, Abcam, USA), and affinity-purified Alexa Fluor 488-conjugated and 594-conjugated secondary antibodies (Servicebio, Hubei, China) were employed for fluorescence detection. Cellular nuclei were counterstained with DAPI (Sigma, USA) to visualize nuclear morphology, and samples were mounted using antifade medium (Vector Laboratories Inc., CA, USA) to preserve fluorescence signal integrity, as previously described ^2, 4^[.](#_ENREF_4)

**Enzyme-linked immunosorbent assay (ELISA)**

Levels of IL-1β (EK101B), IL-6 (EK106/2), IL-17A (EK117/2), and TNF-α (EK182) in human serum were measured using ELISA kits from MULTISCIENCES (Zhejiang, China). Cardiac biomarkers BNP (ab193694, Abcam, USA) and sST2 (E-EL-H6082, Elabscience Biotechnology Co.,Ltd., Wuhan, China) were quantified with ELISA kits, following standardized protocols provided by respective manufacturers. Human myocardial tissue concentrations of p16 (H01273) were measured using ELISA kits (Yifeixue Biotechnology, Nanjing, China). In murine models, serum brain natriuretic peptide (BNP) (YFXEM00760) levels were determined at 4 weeks following MI using ELISA kits (Yifeixue Biotechnology) following standardized protocols. The protein levels of GSSG (oxidized glutathione) and GSH (reduced glutathione) (A061-2-1), nicotinamide adenine dinucleotide including NAD⁺ (oxidized form) and NADH (reduced form) (A114-1-1) were measured with ELISA kits (Nanjing Jiancheng Bioengineering Institute, China) following manufacturer's protocols, as previously described [^1^](#_ENREF_1).

**Senescence-associated-β-gal staining**

SA-β-gal activity was quantitatively evaluated via Senescence β-Galactosidase Staining Kit (#C0602, Beyotime) following manufacturer's instructions as previously described ^12, 13^[.](#_ENREF_13)

**Co-immunoprecipitation**

Co-immunoprecipitation (Co-IP) assays were conducted via Pierce® Co-Immunoprecipitation Kits (#26149, Pierce Biotechnology, Rockford, IL, USA) as previously described ^4, 14^[. Cell lysates derived from HCFB, MRC-5, or HEK293T cells were incubated in cold room (4℃) for 8h with either IgG control antibodies or target-specific precipitation antibodies, including anti-p16 (ab211542, Abcam, USA), anti-STAT3 (#9139, Cell Signaling Technology, USA), anti-Myc Tag (#2276, Cell Signaling Technology, USA), or anti-DYKDDDDK Tag (epitope-matched to Sigma's Anti-FLAG M2 antibody, #14793, Cell Signaling Technology, USA). Resulting co-precipitates and products were measured by Western blot.](#_ENREF_14)

**Western blots**

Myocardial tissue from the infarcted anterior wall and HCFB, MRC-5, or HEK293T cells were lysed in RIPA buffer (#P0013B, Beyotime Biotechnology, China) supplemented with protease/phosphatase inhibitor cocktail (#4906845001, Roche) and phenylmethanesulfonyl fluoride (PMSF, #ST506, Beyotime) to isolate total protein. Western blot analyses were conducted following established protocols as described ^13, 15^[. Primary antibodies were against p53 (#60283-2-Ig](#_ENREF_15), Proteintech, IL, USA), p21 (#10355-1-AP, Proteintech, USA), p16 (ab211542, Abcam, USA), Collagen 1 (#1310-08, Southern Biotech, USA), α-SMA (ab28052, Abcam, USA), IL-1β (ab9722, Abcam, USA) (labeling pro-IL-1β and IL-1β), Cleaved-IL-1β (Asp116) (#AF4006, Affinity Biosciences, USA) (labeling IL-1β), IL-1β (#16806-1-AP, Proteintech, USA) (labeling pro-IL-1β), IL-6 (A0286, ABclonal Biotechnology Co., Ltd., USA), TNF-α (GTX110520, GeneTex Inc., USA), Periostin (POSTN) (#66491-1-1g, Proteintech, USA), Monoclonal antibodyNLRP3 (ab263899, Abcam, USA), ASC (ab155970, Abcam, USA), Caspase-1 (ab207802, Abcam, USA), NF-κB-p65 (#8242, Cell Signaling Technology, USA), p-p65(Ser536) (#3033, Cell Signaling Technology, USA), STAT3 (#9139, Cell Signaling Technology, USA), phosphor-STAT3(Tyr705) (#9145, Cell Signaling Technology, USA), di-methyllysine (Di-meth) (PTM-606, PTM Biolab, Zhejiang, China), EZH2 (#5246, Cell Signaling Technology, USA), Myc Tag (#2276, Cell Signaling Technology, USA), DYKDDDDK Tag (binds the same epitope as Sigma's Anti-FLAG M2 antibody, #14793, Cell Signaling Technology, USA), and HA Tag (#2367, Cell Signaling Technology, USA). β-actin (AP0060, Bioworld Technology Inc., MN, USA) or GAPDH (ab181602, Abcam, USA) was control.

**Chromatin immunoprecipitation (ChIP) assay**

ChIP assays were carried out using the Magna ChIP^TM^ Chromatin Immunoprecipitation A Kit (Millipore) as previously described, following the manufacturer’s protocol [^13^](#_ENREF_13).

Chromatin samples were incubated with antibody against STAT3 (#9139), EZH2 (#5246), H3K27me3 (#4909), H2AK119ub (#8240), Bmi-1 (#6964), AhR (#83200) or BCL6 (Ab241549). The antibodies (STAT3, EZH2, H3K27me3, H2AK119ub, Bmi-1and AhR) were purchased from Cell Signaling Technology, and BCL6 were from Abcam. The antibody-bound DNA samples were assessed via real-time quantitative PCR (RT-qPCR).

**Re-ChIP assay**

Re-ChIP assay was performed using the Re-ChIP kit (#53016, Active Motif, Shanghai, China) following the manufacturer’s instruction. Cell processing followed the identical procedure as ChIP assay up to the antibody addition step. Primary antibody was pre-bound to Protein A agarose beads in BSA (0.5%) -PBS at 4℃ for 8h with shaking and washed twice with 1x IP Buffer before chromatin incubation at 4℃ overnight. Beads were sequentially washed with low/high-salt, LiCl, and TE buffers in cold room. Immunoprecipitates were eluted at 37℃ for 30 min via elution buffer (1% SDS, 0.1 M NaHCO_3_, 10 mM DTT). Eluate (100 μl) was diluted 20-fold in 1X IP Buffer and incubated at 4℃ for 8h with secondary antibody pre-coupled to Protein A beads. Post-wash elution at 65℃ for 30 min was followed by cross-link reversal at 65℃ overnight via proteinase K (20 mg/ml) and RNase A (10 mg/ml) (Sigma, USA). DNA was purified, dissolved in DEPC water and stored at -20℃ for RT-qPCR. Antibodies were consistent between ChIP and Re-ChIP assays.

**RNA Isolation and RT-qPCR Analyses**

Total RNA was isolated from myocardial infarct (anterior wall) tissue and HCFB, MRC-5, and HEK293T cells via TRIzol® Reagent (Invitrogen). cDNA was synthesized with SuperScript™ III First-Strand Synthesis SuperMix (Invitrogen). RT-qPCR and ChIP-qPCR were performed using SYBR® Green Master Mix (Yeasen Biotech) on Real-Time PCR System (Applied Biosystems). Relative mRNA expression was normalized to *Gapdh* via the 2⁻^ΔΔCt^ approach. Sequences of all primers for RT-qPCR and ChIP-qPCR are listed in **Supplemental Information 9_Table S2**.

**Plasmid engineering and adenoviral transfection**

A Flag-tagged human *p16* overexpression adenovirus was constructed in Genechem Co., Ltd. (Shanghai, China).

Guided by structural analyses of human STAT3, we generated six mutant fragments including N-terminal domain-deleted mutant (ΔN-D), coiled-coil domain-deleted mutant (ΔCCD), DNA-binding domain-deleted mutant (ΔDBD), linker region-deleted mutant (ΔLinker), SH2 domain-deleted mutant (ΔSH2), and C-terminal domain-deleted mutant (ΔC-D), and a full-length fragment overexpression plasmid with His-tag cloned in pcDNA3.1 vector plasmid. All plasmids synthesis was performed by TranSheep Bio Co., Ltd. (Shanghai, China).

HEK293T cells were transfected with Flag-*p16* (human) overexpression adenovirus in complete medium at 40%-50% confluency for 8 hours. Subsequently, plasmids were transfected via Lipofectamine 2000 (#11668-019, Invitrogen Inc., USA) following manufacturer's recommended protocol, when cells reached 60-70% confluency. Transfection complexes remained in complete medium for 6 hours, after which cells were maintained in fresh medium and harvested 48 hours post-transfection. All procedures adhered to standard transfection protocols as previously described [^14^](#_ENREF_14).

**Dual luciferase assay**

A mutant plasmid (K to R mutation, K49R) of STAT3 was generated by site-directed mutagenesis at the K49 residue and transfected into HEK293T cells. *NLRP3* promoter activity was evaluated using a luciferase reporter assay as previously described [^16^](#_ENREF_16). The human *STAT3* coding sequence was cloned into pcDNA3.1 vector (TranSheep Bio, China), while *NLRP3* promoter reporter constructs were engineered in pGL4.1-basic vector (TranSheep Bio, China) by inserting luciferase upstream of 5′-flanking region of human *NLRP3* gene.

The Flag-*p16* (human) overexpression adenovirus was introduced into HEK293T cells, which were cultured in medium at 40-50% confluence, for 8 hours. Twenty-four hours post-transduction, *STAT3*-WT or K49R-mutant overexpression plasmids (pcDNA3.1) were co-transfected with pGL4.1-NLRP3 promoter reporter and dual-luciferase constructs (Fireﬂy luciferase/Renilla luciferase) into HEK293T cells using Lipofectamine 2000 (Invitrogen, #11668-019, USA). Luciferase activity was detected 48 hours post-transfection via commercial detection kit (Promega Corporation, WI, USA) as previously described [^16^](#_ENREF_16).

**Small interference RNA-mediated knockdown of human *STAT3***

RNA primers specific to human STAT3 were synthesized by Ribobio Co. Ltd. (China) for siRNA experiments. HCFB and MRC-5 cells were cultured in antibiotic-free DMEM at number of 2 × 10^5^ cells per well in 6-well plates and incubated until reaching 50% confluence. One day prior to transfection, *STAT3* siRNA (50 nM) and Lipofectamine 2000 (#11668-019, Invitrogen, USA) were independently diluted in Opti-MEM, then combined and incubated. At 6 h post-transfection, culture medium was exchanged for DMEM with 10% FBS, followed by 72 h incubation. Cells were subsequently collected for protein extraction. Sequences of siRNAs and targeted mRNAs are in **Supplemental Information 9 Table S3**.

**NSC74859 treatment**

HCFB and MRC-5 cells were treated with NSC74859 (S1155, Selleck Chemicals, TX, USA) at 50μM for 24 h as previously described [^17^](#_ENREF_17).

**GSK343 or GSK503** **treatment**

HCFB cells were treated with GSK343 (S7164, Selleck Chemicals, USA) at 5μM or GSK503 (S7804, Selleck Chemicals, USA) at 10μM for 24 h.

**Statistical analysis**

Quantitative data are reported as mean ± standard error of the mean (SEM) from ≥3 independent experiments. Statistical analyses were conducted using SPSS v22.0 and GraphPad Prism v7.0. Intergroup comparisons utilized unpaired Student's *t*-tests, while one-way ANOVA was employed for multiple group analyses. Statistical significance was defined as P<0.05 (two-sided). Correlation analyses applied Pearson's coefficient for normal distributions and Spearman's for non-normal ones, as previously described [^2^](#_ENREF_2).

**References**

1. Gu X, Meng H, Peng C*, et al.* Inflammasome activation and metabolic remodelling in p16-positive aging cells aggravates high-fat diet-induced lung fibrosis by inhibiting NEDD4L-mediated K48-polyubiquitin-dependent degradation of SGK1. *Clin Transl Med* 2023; **13**: e1308.

2. Chen H, Chen H, Liang J*, et al.* TGF-beta1/IL-11/MEK/ERK signaling mediates senescence-associated pulmonary fibrosis in a stress-induced premature senescence model of Bmi-1 deficiency. *Experimental & molecular medicine* 2020; **52**: 130-151.

3. Jin J, Zhao Y, Tan X, Guo C, Yang Z, Miao D. An improved transplantation strategy for mouse mesenchymal stem cells in an acute myocardial infarction model. *PLoS One* 2011; **6**: e21005.

4. Chen H, Zhou J, Chen H*, et al.* Bmi-1-RING1B prevents GATA4-dependent senescence-associated pathological cardiac hypertrophy by promoting autophagic degradation of GATA4. *Clin Transl Med* 2022; **12**: e574.

5. Li L, Wang D, Wang X*, et al.* N-Butyrylated hyaluronic acid ameliorates gout and hyperuricemia in animal models. *Pharmaceutical biology* 2019; **57**: 717-728.

6. Chen H, Wang Q, Li J*, et al.* IFNgamma Transcribed by IRF1 in CD4+ Effector Memory T Cells Promotes Senescence-Associated Pulmonary Fibrosis. *Aging Dis* 2023; **14**: 2215-2237.

7. Jifu C, Lu L, Ding J*, et al.* USP18 Is Associated with PD-L1 Antitumor Immunity and Improved Prognosis in Colorectal Cancer. *Biomolecules* 2024; **14**: 1191.

8. Fleck JS, Jansen SMJ, Wollny D*, et al.* Inferring and perturbing cell fate regulomes in human brain organoids. *Nature* 2023; **621**: 365-372.

9. Kumar S, Nagesh D, Ramasubbu V, Prabhashankar AB, Sundaresan NR. Isolation and Culture of Primary Fibroblasts from Neonatal Murine Hearts to Study Cardiac Fibrosis. *Bio Protoc* 2023; **13**: e4616.

10. Fang RH, Hu CM, Luk BT*, et al.* Cancer cell membrane-coated nanoparticles for anticancer vaccination and drug delivery. *Nano Lett* 2014; **14**: 2181-2188.

11. Wang Q, Song Y, Chen J*, et al.* Direct in vivo reprogramming with non-viral sequential targeting nanoparticles promotes cardiac regeneration. *Biomaterials* 2021; **276**: 121028.

12. Chen B, Zhang W, Gao J*, et al.* Downregulation of ribosomal protein S6 inhibits the growth of non-small cell lung cancer by inducing cell cycle arrest, rather than apoptosis. *Cancer Lett* 2014; **354**: 378-389.

13. Zhou J, Chen H, Wang Q*, et al.* Sirt1 overexpression improves senescence-associated pulmonary fibrosis induced by vitamin D deficiency through downregulating IL-11 transcription. *Aging cell* 2022; **21**: e13680.

14. Zhao J, Chen A, Wang R*, et al.* Bmi-1 Epigenetically Orchestrates Osteogenic and Adipogenic Differentiation of Bone Marrow Mesenchymal Stem Cells to Delay Bone Aging. *Adv Sci (Weinh)* 2024: e2404518.

15. Wang Q, Zhao J, Chen H*, et al.* Bmi-1 Overexpression Improves Sarcopenia Induced by 1,25(OH)(2) D(3) Deficiency and Downregulates GATA4-Dependent Rela Transcription. *J Bone Miner Res* 2023; **38**: 427-442.

16. Sun H, Qiao W, Cui M*, et al.* The Polycomb Protein Bmi1 Plays a Crucial Role in the Prevention of 1,25(OH)2 D Deficiency-Induced Bone Loss. *J Bone Miner Res* 2020; **35**: 583-595.

17. Lin L, Amin R, Gallicano GI*, et al.* The STAT3 inhibitor NSC 74859 is effective in hepatocellular cancers with disrupted TGF-beta signaling. *Oncogene* 2009; **28**: 961-972.
