# Supplementary material for: Fibroblasts‐specific p16INK4a exacerbates inflammageing‐mediated post‐infarction ventricular remodelling through interacting with STAT3 to regulate NLRP3 transcription
Source: Clin Transl Med. 2025 Jun 3;15(6):e70344. doi: 10.1002/ctm2.70344 (PMC12134396; doi:10.1002/ctm2.70344)
Supplement: Supplementary file 6 — SI6: Basic information of coronary artery disease and MI patients [file CTM2-15-e70344-s001.docx]

|  | CAD (n=20) | MI (n=20) | *P* value |
| --- | --- | --- | --- |
| Male (%) | 12 (60%) | 13 (65%) |  |
| Age | 65.05±11.88 | 61.10±11.18 | 0.5961 |
| Hypertension (%) | 15 (75%) | 13 (65%) |  |
| Diabetes (%) | 7 (35%) | 10 (50%) |  |
| Systolic blood pressure (mmHg) | 135.3±16.70 | 138.3±19.40 | 0.8619 |
| Diastolic pressure (mmHg) | 82.00±13.16 | 78.85±15.96 | 0.5001 |
| Creatinine (μmol/L) | 67.78±32.19 | 75.55±33.57 | 0.4596 |
| Heart Reat (bpm/min) | 95.60±17.64 | 89.15±13.81 | 0.2057 |
| eGFR (ml/min·1.73m^2^) | 94.70±16.37 | 97.25±21.29 | 0.6735 |
| AST (U/L) | 35.20±15.57 | 37.35±15.99 | 0.6691 |
| ALT (U/L) | 33.25±11.01 | 35.15±11.27 | 0.5929 |
| Triglycerides Level (mmol/L) | 2.290±0.9284 | 2.460±0.9939 | 0.5794 |
| Low density lipoprotein (mmol/L) | 2.461±1.010 | 2.605±1.226 | 0.6875 |
| Blood Glucose | 7.405±2.562 | 8.840±3.590 | 0.1538 |
| Anti-Hypertension Drugs (%) | 12 (60%) | 13 (65%) |  |
| Anti-Platelet Drugs (%) | 20 (100%) | 20 (100%) |  |
| Hemoglobin (g/L) | 125.5±13.50 | 128.80±15.15 | 0.4648 |
| Blood platelet (*10^9^/L) | 226.90±72.43 | 212.9±60.27 | 0.5119 |

**SI6: Basic information of CAD and MI patients**
