# Supplementary material for: Fibroblasts‐specific p16INK4a exacerbates inflammageing‐mediated post‐infarction ventricular remodelling through interacting with STAT3 to regulate NLRP3 transcription
Source: Clin Transl Med. 2025 Jun 3;15(6):e70344. doi: 10.1002/ctm2.70344 (PMC12134396; doi:10.1002/ctm2.70344)
Supplement: Supplementary file 7 — SI7: Basic information of MI patients of different ages [file CTM2-15-e70344-s006.docx]

|  | MI (Age<65) | MI (Age>75) | *P* value |
| --- | --- | --- | --- |
| Sex (%) | 14 (70%) | 11 (55%) |  |
| Age | 58.35±2.033 | 78.40±2.437 | <0.0001 |
| Hypertension (%) | 14 (70%) | 16 (80%) |  |
| Diabetes (%) | 7 (35%) | 12 (60%) |  |
| Systolic blood pressure (mmHg) | 127.9±20.34 | 131.0±17.66 | 0.6155 |
| Diastolic pressure (mmHg) | 81.95±8.982 | 83.25±12.11 | 0.7019 |
| Creatinine (μmol/L) | 94.95±18.02 | 106.9±23.89 | 0.0833 |
| Heart Reat (bpm/min) | 79.15±13.33 | 87.4±13.45 | 0.0588 |
| eGFR (ml/min·1.73m^2^) | 96.4±16.96 | 88.35±19.26 | 0.1688 |
| Serum AST (U/L) | 29.15±9.582 | 29.6±10.04 | 0.8855 |
| Serum ALT (U/L) | 35.8±16.1 | 35.55±16.64 | 0.9617 |
| Triglycerides Level (mmol/L) | 2.52±1.033 | 2.57±1.263 | 0.8917 |
| Low density lipoprotein (mmol/L) | 2.663±0.8086 | 2.82±0.9216 | 0.5690 |
| Blood Glucose | 6.74±0.9287 | 7.67±2.191 | 0.0855 |
| Anti-Hypertension Drugs (%) | 14 (70%) | 16 (80%) |  |
| Anti-Platelet Drugs (%) | 20 (100%) | 20 (100%) |  |
| Hb (g/L) | 135.6±13.62 | 128.7±14.86 | 0.1341 |
| PTL (*10^9^/L) | 244.3±47.08 | 239.1±66.43 | 0.7767 |

**SI7: Basic information of MI patients of different ages**
