# Supplementary material for: Fibroblasts‐specific p16INK4a exacerbates inflammageing‐mediated post‐infarction ventricular remodelling through interacting with STAT3 to regulate NLRP3 transcription
Source: Clin Transl Med. 2025 Jun 3;15(6):e70344. doi: 10.1002/ctm2.70344 (PMC12134396; doi:10.1002/ctm2.70344)
Supplement: Supplementary file 9 — SI9: Tables S1‐S3 [file CTM2-15-e70344-s004.docx]

**SI9 Tables S1-S2**

**Table S1 siRNAs against Mouse p16**

| Name | S/AS | Sequence | Target mRNA Sequences |
| --- | --- | --- | --- |
| siRNA2  Negative control siRNA | S  AS  S  AS | 5′-CAUCAAGACAUCGUGCGAUAUdTdT-3′  5′-AUAUCGCACGAUGUCUUGAUGdTdT-3′  5′-UUCUCCGAACGUGUCACGUdTdT-3′  5′-ACGUGACACGUUCGGAGAAdTdT-3′ | TCAAGACATCGTGCGATATTT  — |

S, sense; AS, antisense

**Table S2 Primers for RT-qPCR or ChIP-qPCR**

| Name | S/AS | Sequence | Species | Tm  (°C) | Length  (bp) |
| --- | --- | --- | --- | --- | --- |
| *CDKN2A*  *S100A8*  *S100A9*  *CXCL2*  *COL1A1*  *BIRC3*  *NNMT*  *NAMPT*  *TNFRSF1A*  *Myc*  *NLRP3*  *Cxcl2*  *S100a8*  *S100a9*  *Nlrp3*  *Ccl6*  *Ccl9*  *Aim2*  *Ccr7*  *Hmgb2*  *IL-18*  *Gpx3*  *Gstp2*  *Gstm1*  *Gstp1g*  *Gclc*  *Gclm*  *Slc7a11*  *Col1α1*  *Acta2*  *Postn*  *Fn1*  *Gstm6*  *IL-1β*  *IL-6*  *TNF-α*  *Gapdh* | S  AS  S  AS  S  AS  S  AS  S  AS  S  AS  S  AS  S  AS  S  AS  S  AS  S  AS  S  AS  S  AS  S  AS  S  AS  S  AS  S  AS  S  AS  S  AS  S  AS  S  AS  S  AS  S  AS  S  AS  S  AS  S  AS  S  AS  S  AS  S  AS  S  AS  S  AS  S  AS  S  AS  S  AS  S  AS  S  AS  S  AS | 5'-ATGGAGCCTTCGGCTGACT-3'  5'-GTAACTATTCGGTGCGTTGGG-3'  5′-ATGCCGTCTACAGGGATGAC-3′  5′-ACTGAGGACACTCGGTCTCTA-3′  5′-GGTCATAGAACACATCATGGAGG-3′  5′-GGCCTGGCTTATGGTGGTG-3′  5′-ATGTCTTTCTTGTAAGGCATACTG-3′  5′-CGAAACCTCTCTGCTCTAACAC-3′  5′-GAGGGCCAAGACGAAGACATC-3′  5′-CAGATCACGTCATCGCACAAC-3′  5'-TCCTCTGACCCACGAGCAAT-3'  5'-CTCTTAATCAGTGGCCTGGCA-3'  5'-TACCCCTACCACCGGACAAT-3′  5'-AAAAGCCAGGATCAGGACGG-3'  5′-CTGCCCCCAGATAGAATGAGAC-3′  5′-TCATGCGTGGTTGCGTTTTT-3′  5′-AGTCAGGGCAGAAAACGAGG-3′  5′-GCGTCCGACACATGATAGGT-3′  5′-GTGCATGACCGCATTTCCAA-3′  5′-CGGACTTCCTAAAAGGGGCA-3′  5′-CGAATGACACCTTTCACCTGG-3′  5′-GAGAGGCATCTAGTCCTGCTT-3′  5′-CCAACCACCAGGCTACAGG-3′  5′-GCGTCACACTCAAGCTCTG-3′  5′-AAATCACCATGCCCTCTACAAG-3′  5′-CCCACTTTTATCACCATCGCAA-3′  5′-ATACTCTAGGAAGGAAGGACACC-3′  5′-TCCATGATGTCATTTATGAGGGC-3′  5′-ATTACCCGCCCGAGAAAGG-3′  5′-TCGCAGCAAAGATCCACACAG-3′  5′-GCTGGCCTCATACAAGAAATGG-3′  5′-GCTTAGGCACCTCTGAACTCTC-3′  5′-CCCTCTCCTTCCTCATTCTTACA-3′  5′-AGTCTTGAAAGCCCATGTGAAA-3′  5′-GTCACCAGTTCCTCAGTTGTG-3′  5′-CACCTCCATTGTCCCTGTTTTAT-3′  5′-TGTACGAGTCGGTGTGCTTC-3′  5′-GGTAGGTATCCGTCATGGTCTTG-3′  5′-GGTAAGGGTGACCCCATCAAG-3′  5′-CGACGAATTGGGATGCTTCTT-3′  5′-GACTCTTGCGTCAACTTCAAGG-3′  5′-CAGGCTGTCTTTTGTCAACGA-3′  5′-CCTTTTAAGCAGTATGCAGGCA-3′  5′-CAAGCCAAATGGCCCAAGTT-3′  5′-ACTTCTCTCTGCACAGCAGCC-3′  5′-GCCCATACAGACAAGTGGGC-3′  5′-ATACTGGGATACTGGAACGTCC-3′  5′-AGTCAGGGTTGTAACAGAGCAT-3′  5′-ATGCCACCATACACCATTGTC-3′  5′-GGGAGCTGCCCATACAGAC-3′  5′-GGGGTGACGAGGTGGAGTA-3′  5′-GTTGGGGTTTGTCCTCTCCC-3′  5′-AGGAGCTTCGGGACTGTATCC-3′  5′-GGGACATGGTGCATTCCAAAA-3′  5′-GGCACCGTCATCGGATCAG-3′  5′-CTCCACAGGCAGACCAGAAAA-3′  5′-TAAGGGTCCCCAATGGTGAGA-3′  5′-GGGTCCCTCGACTCCTACAT-3′  5′-GGCACCACTGAACCCTAAGG-3′  5′-ACAATACCAGTTGTACGTCCAGA-3′  5′-TGGTATCAAGGTGCTATCTGCG-3′  5′-AATGCCCAGCGTGCCATAA-3′  5′-ATGTGGACCCCTCCTGATAGT-3′  5′-GCCCAGTGATTTCAGCAAAGG-3′  5′-TTGGAGAACAGGGTCATGGAC-3′  5′-GGGTTCAAACATTCGATGCTGA-3′  5′-TGCCACCTTTTGACAGTGATG-3′  5′-TGATGTGCTGCTGCGAGATT-3′  5′-CCCCAATTTCCAATGCTCTCC-3′  5′-CGCACTAGGTTTGCCGAGTA-3′  5′-GATCGGTCCCCAAAGGGATG-3′  5′-TTTGCTACGACGTGGGCTAC-3′  5′-CATTTCACTCAAGGTTGTCAGC-3′  5′-ATCATACTTGGCAGGTTTCTCC-3′ | human  human  human  human  human  human  human  human  human  human  human  mouse  mouse  mouse  mouse  mouse  mouse  mouse  mouse  mouse  mouse  mouse  mouse  mouse  mouse  mouse  mouse  mouse  mouse  mouse  mouse  mouse  mouse  mouse  mouse  mouse  mouse | 60  58  60  58  60  60  60  60  60  60  60  60  60  60  60  60  60  58  60  60  60  58  60  60  60  60  60  60  60  60  60  60  60  60  60  60  60 | 108  54  155  81  140  127  366  187  155  167  75  108  165  129  141  246  141  213  162  102  169  120  175  349  161  125  105  100  203  135  135  124  219  138  141  129  346 |

S, sense; AS, antisense, sequence; Tm, annealing temperature; length, amplicon

**Table S3 siRNAs against Human STAT3**

| Name | S/AS | Sequence | Target mRNA Sequences |
| --- | --- | --- | --- |
| siRNA 1  Negative control siRNA | S  AS  S  AS | 5′-CCACUUUGGUGUUUCAUAA dTdT-3′  5′-dTdT CTCAGAGGATCCCGGAAATTT-3′  5′-UUCUCCGAACGUGUCACGUTTdTdT-3′  5′-dTdTACGUGACACGUUCGGAGAATT-3′ | TCTCAGAGGATCCCGGAAATTT  — |

S, sense; AS, antisense
